# Supramolecular Macrocyclic Iodine Adsorbents Enable Photothermally Stable Perovskite Solar Cells

**DOI:** 10.1002/advs.202516964

**Published:** 2025-10-30

**Authors:** Yue Wu, Wen Li, Shengzhong Li, Yan‐Fei Niu, Cuihong Wang, Hai‐Bo Yang, Xiao‐Li Zhao, Xiaodong Li, Junfeng Fang, Xueliang Shi

**Affiliations:** ^1^ State Key Laboratory of Petroleum Molecular & Process Engineering Shanghai Key Laboratory of Green Chemistry and Chemical Processes School of Chemistry and Molecular Engineering East China Normal University Shanghai 200062 China; ^2^ School of Physics and Electronic Science Engineering Research Center of Nanophotonics & Advanced Instrument Ministry of Education East China Normal University Shanghai 200062 China

**Keywords:** host–guest chemistry, iodine adsorption, macrocycle, perovskite solar cells, stability

## Abstract

In this study, the design and synthesis of two novel diazapentacene‐based macrocycles (**M3** and **M4**) is reported via a one‐pot Yamamoto coupling reaction. These macrocycles are constructed by π‐extension of a dihydrophenazine core, maintaining its excellent redox activity while offering enlarged cavities and enhanced electron‐donating properties. As a result, **M3** and **M4** exhibit strong electron‐rich characteristics and well‐defined cavities, enabling their use as efficient iodine adsorbents to mitigate photo‐thermal‐induced iodine loss and perovskite degradation in solar cells. The macrocycles demonstrate dual‐mode iodine capture: physical adsorption through cavity confinement and chemical adsorption via charge‐transfer interactions, both of which show excellent reversibility. Addressing the critical issue of operational instability in perovskite solar cells (PSCs), caused by iodine escape and Pb⁰ defect formation, these macrocycles effectively trap volatile iodine species and suppress defect generation. Notably, PSCs incorporating macrocycle **M4** achieve a high efficiency of 26.13% and outstanding operational stability, retaining ≈95.85% of their initial efficiency after 1000 h of maximum power point (MPP) tracking at 85  °C under the International Summit on Organic Photovoltaic Stability‐Light‐Soaking Test at 65/85 °C (ISOS‐L‐2) protocol.

## Graphical abstract

Two novel diazapentacene‐based macrocycles are synthesized with expanded cavities that enable dual‐mode iodine adsorption through both physical and chemical interactions. When applied as iodine adsorbents in solar cells, PSCs incorporating **M4** achieve 26.13% efficiency with 95.85% retention after 1000 h at 85 °C.

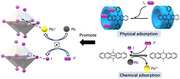

## 1 Introduction

Supramolecular macrocyclic hosts, such as cyclodextrins (CD),^[^ ^1^ ^]^ calix[n]arenes (CA[n]),^[^ ^2^ ^]^ cucurbit[n]urils (CB[n]),^[^ ^3^ ^]^ pillar[n]arenes (P[n]),^[^ ^4^ ^]^ and others,^[^ ^5^ ^]^ have been widely applied in host‐guest chemistry due to their well‐defined cavities and versatile binding sites, making them excellent molecular containers. These macrocycles have also been extensively explored as advanced adsorbents for the removal of various hazardous substances, including organic micropollutants,^[^ ^6^ ^]^ metal ions,^[^ ^7^ ^]^ perfluoroalkyl substances,^[^ ^8^ ^]^ iodine species^[^ ^9^, ^10^ ^]^ and so on.^[^ ^11^, ^12^ ^]^ Among these, radioactive iodine isotopes (e.g., ^129^I and ^131^I) are of particular concern due to their high volatility, environmental mobility, and significant radiotoxicity. For capturing electron‐deficient iodine species, electron‐rich macrocycles,^[^ ^13^ ^]^ especially those containing nitrogen atoms, offer strong binding affinity.^[^ ^14^ ^]^ Nitrogen‐embedded macrocycles, such as pyridine‐based bis‐urea macrocycles,^[^ ^15^ ^]^ Schiff base macrocycles,^[^ ^16^, ^17^ ^]^ Tröger's base‐based macrocycles,^[^ ^18^, ^19^ ^]^ aniline‐based macrocycles,^[^ ^20^ ^]^ and ‐containing macrocycle,^[^ ^21^ ^]^ have demonstrated excellent iodine uptake, underscoring the crucial role of nitrogen‐rich motifs in adsorption performance. Beyond macrocycles, supramolecular cages,^[^ ^22^, ^23^ ^]^ metal‐organic frameworks (MOFs),^[^ ^24^, ^25^ ^]^ and covalent organic frameworks (COFs)^[^ ^26^, ^27^ ^]^ have also emerged as effective platforms for iodine capture. Therefore, the development of novel nitrogen‐rich macrocyclic hosts with good iodine adsorption capability and expanded functional applications remains a key challenge and opportunity in supramolecular chemistry and materials science.

**Figure 1:**
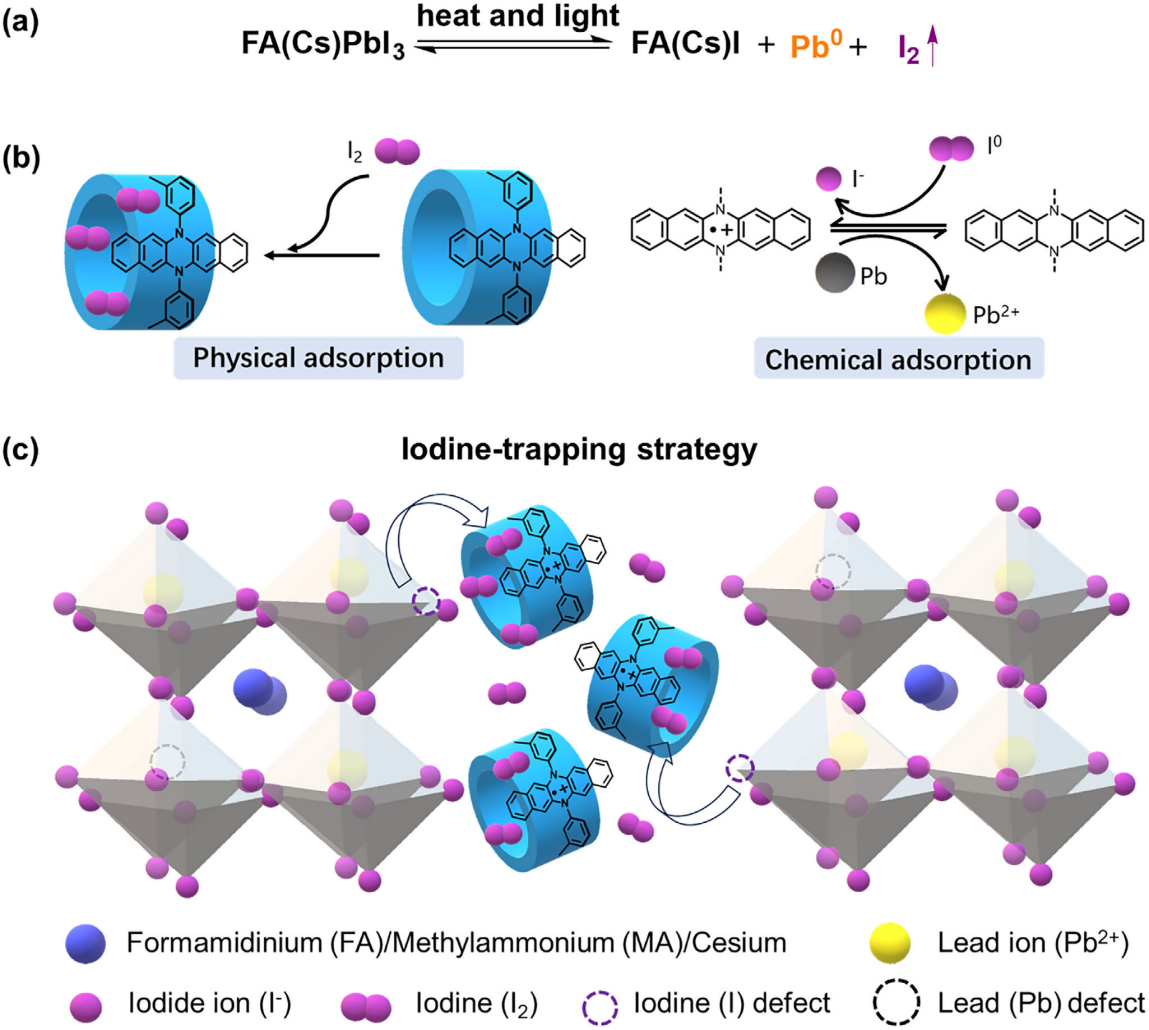
Supramolecular macrocyclic iodine‐trapping strategy to improve the long‐term stability of perovskite solar cells (PSCs). a) I_2_ and Pb^0^ generation during perovskite degradation under heat and light conditions. b) Illustration of iodine capture with diazapentacene based redox‐active macrocycles. c) The iodine‐trapping and regeneration strategy of macrocycles in the perovskite.

Beyond conventional applications in environmental iodine remediation, iodine adsorbents have recently emerged as powerful tools for enhancing the intrinsic stability of perovskite materials.^[^ ^28^, ^29^, ^30^, ^31^ ^]^ Perovskites (whether hybrid perovskite^[^ ^32^, ^33^ ^]^ or inorganic perovskite^[^ ^34^ ^]^) are revolutionizing the photovoltaic industry due to their high power conversion efficiency and low manufacturing cost.^[^ ^35^, ^36^, ^37^ ^]^ However, device stability remains a critical challenge, particularly under real‐world operating conditions involving elevated temperatures and continuous illumination. Under such thermal and photonic stress, perovskite materials decompose into volatile I_2_ and non‐volatile Pb^0^. The escape of I_2_ not only leaves behind Pb^0^ defects, accelerating material degradation,^[^ ^38^ ^]^ but also corrodes metal electrodes and damages the electron transport layer.^[^ ^39^ ^]^ Therefore, suppressing I_2_ volatilization and promoting PbI_2_ regeneration are key to improving the long‐term stability of perovskite solar cells (PSCs) (**Figure** **1**). To address this, various iodine‐capturing strategies have been developed, including reversible iodine‐alkene reactions,^[^ ^28^ ^]^ iodine‐trapping or ‐confinement methods,^[^ ^29^, ^30^ ^]^ and the use of redox‐active supramolecular assemblies.^[^ ^31^ ^]^ These approaches have significantly improved both performance and durability of PSCs. Notably, our group has demonstrated that β‐cyclodextrin, a macrocyclic host, can effectively capture iodine and enable stable device operation at elevated temperatures.^[^ ^29^ ^]^ Furthermore, incorporating redox‐active supramolecular assemblies into perovskite films offers a dual function, trapping iodine and serving as redox shuttles, to facilitate iodine regeneration and defect repair.^[^ ^31^ ^]^ Based on these insights, we propose that a redox‐active supramolecular macrocycle with good iodine‐binding affinity could serve as both an iodine captor and regenerator, paving the way for highly efficient and thermally stable PSCs (Figure 1).

Herein we report the design and synthesis of a novel class of nitrogen‐embedded conjugated macrocyclic iodine adsorbents and demonstrate their application as both iodine captors and regenerators in perovskite solar cells (Figure 1). The macrocycles, **M3** and **M4**, are composed of three and four redox‐active π‐conjugated 6,13‐diphenyl‐6,13‐diazapentacene units, respectively, and are synthesized via a one‐pot Yamamoto coupling reaction. Owing to the electron‐rich character of the dihydrodiazapentacene moieties and the well‐defined cylindrical cavities of the macrocycles, **M3** and **M4** exhibit dual‐mode iodine capture: chemical adsorption via charge‐transfer interactions and physical encapsulation within the macrocyclic cavity. In addition, these macrocycles show good iodine adsorption‐desorption recyclability, enabling the release of captured iodine and promoting perovskite regeneration. As a result, PSCs incorporating **M4** achieve high power conversion efficiency of 26.13% and outstanding operational stability, retaining ≈95.85% of their initial efficiency after 1000 h of maximum power point (MPP) tracking at 85  °C under the International Summit on Organic Photovoltaic Stability‐Light‐Soaking Test at 65/85 °C (ISOS‐L‐2) protocol. While the control PSCs degraded rapidly and retained 62.97% of initial efficiency under those same conditions.

## 2 Results and Discussion

**Figure 2:**
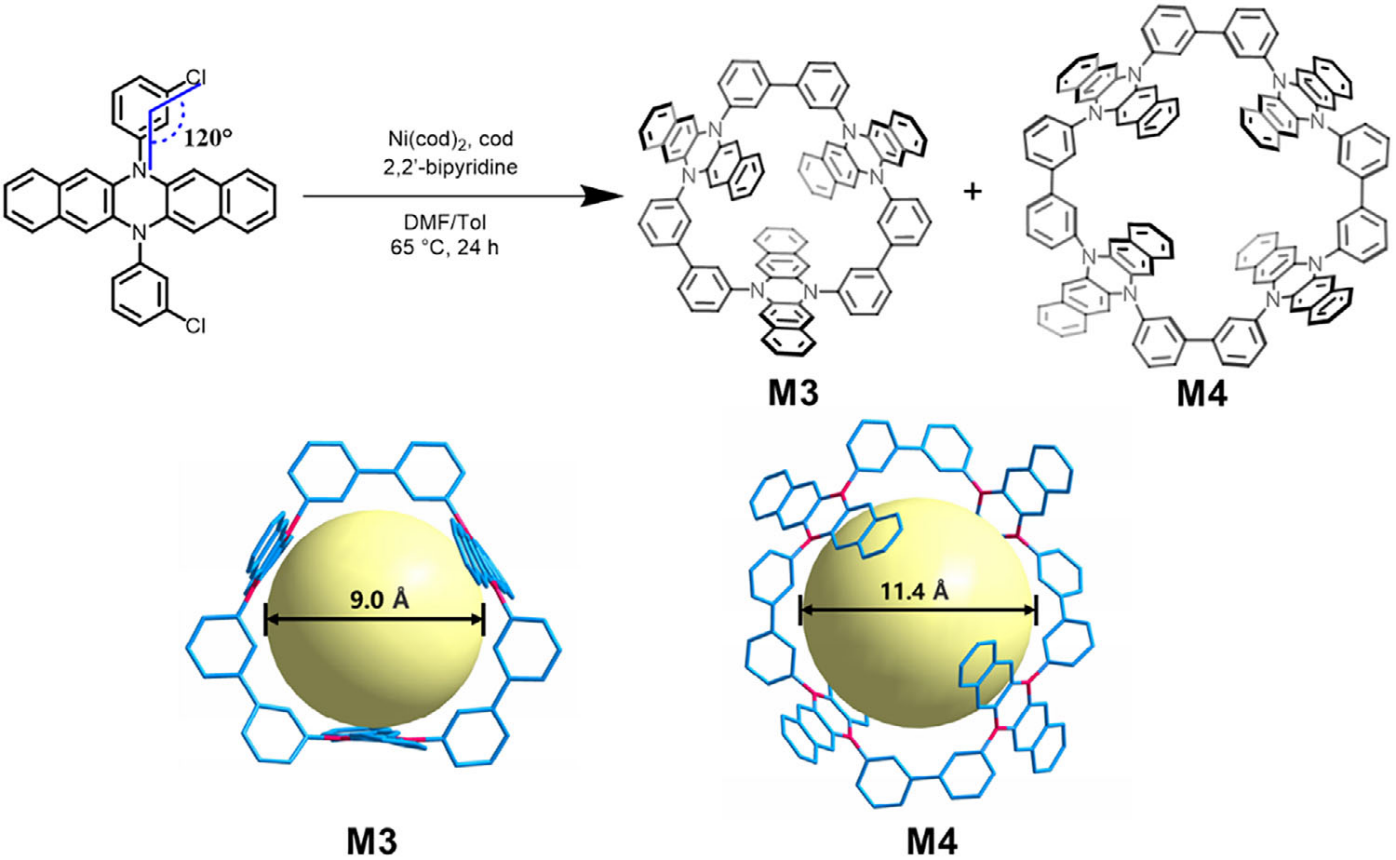
Synthesis and characterization of two macrocycles. Synthesis and X‐ray crystal structures of diazapentacene based macrocycles **M3** and **M4**. The large yellow spheres represent the intrinsic cavities of the macrocycles. C: blue; N: red. Hydrogen atoms and solvent were omitted for clarity.

In our molecular design, 6,13‐diphenyl‐6,13‐diazapentacene unit was strategically selected as the key π‐conjugated building block for the synthesis of macrocycles. By extending the π‐conjugation system through structural expansion of the dihydrophenazine framework,^[^ ^40^, ^41^, ^42^ ^]^ we can achieve simultaneous enhancement of the macrocycles' electron‐rich character and cavity dimensions. This dual‐functional modification not only amplified the electron‐donating capacity but also created an expanded cavity architecture, thereby optimizing the host‐guest interaction for iodine encapsulation. To precisely control the spatial arrangement, 6,13‐bis(3‐chlorophenyl)‐6,13‐diazapentacene was employed as a rigid angular linker with an intrinsic 120° bend angle, which proved critical for directing the formation of well‐defined macrocyclic topologies with enlarged cavities.^[^ ^43^, ^44^ ^]^ The target macrocycles **M3** and **M4** were efficiently synthesized through a one‐pot Yamamoto coupling reaction, achieving isolated yields of 18% and 15% respectively (**Figure** **2**, details see Synthesis section in Supporting Information). The isolation of macrocycles was conducted by gel column chromatography and gel permeation chromatography. Both **M3** and **M4** were characterized by nuclear magnetic resonance (NMR), mass spectrometry (MS), and single‐crystal X‐ray structure analysis (details see NMR and mass spectra section and X‐ray crystallographic analysis section in Supporting Information).

Single crystals of **M3** suitable for X‐ray crystallographic analysis were obtained by slow diffusion of methanol (MeOH) into a dichloromethane (CH_2_Cl_2_) solution. In the crystal structure, **M3** adopts a triangular conformation in which three diazapentacene panels are connected via orthogonal biphenyl linkers at their meta positions, forming a well‐defined cylindrical cavity with a diameter of ≈9.0 Å (Figure 2). Similarly, single crystals of **M4** were obtained by slow diffusion of 1,4‐dioxane into a chloroform (CHCl_3_) solution. **M4** adopts a square‐shaped conformation, with two diazapentacene panels slightly incline toward the cavity and the other two tilt outward from the cavity. As in **M3**, the four diazapentacene panels are connected through meta‐linked biphenyl spacers, resulting in a well‐defined cylindrical cavity with a diameter of 11.4 Å (Figure 2). The rigid macrocyclic frameworks and well‐defined cavities of **M3** and **M4** are expected to facilitate efficient guest binding, particularly for iodine species.

**Figure 3:**
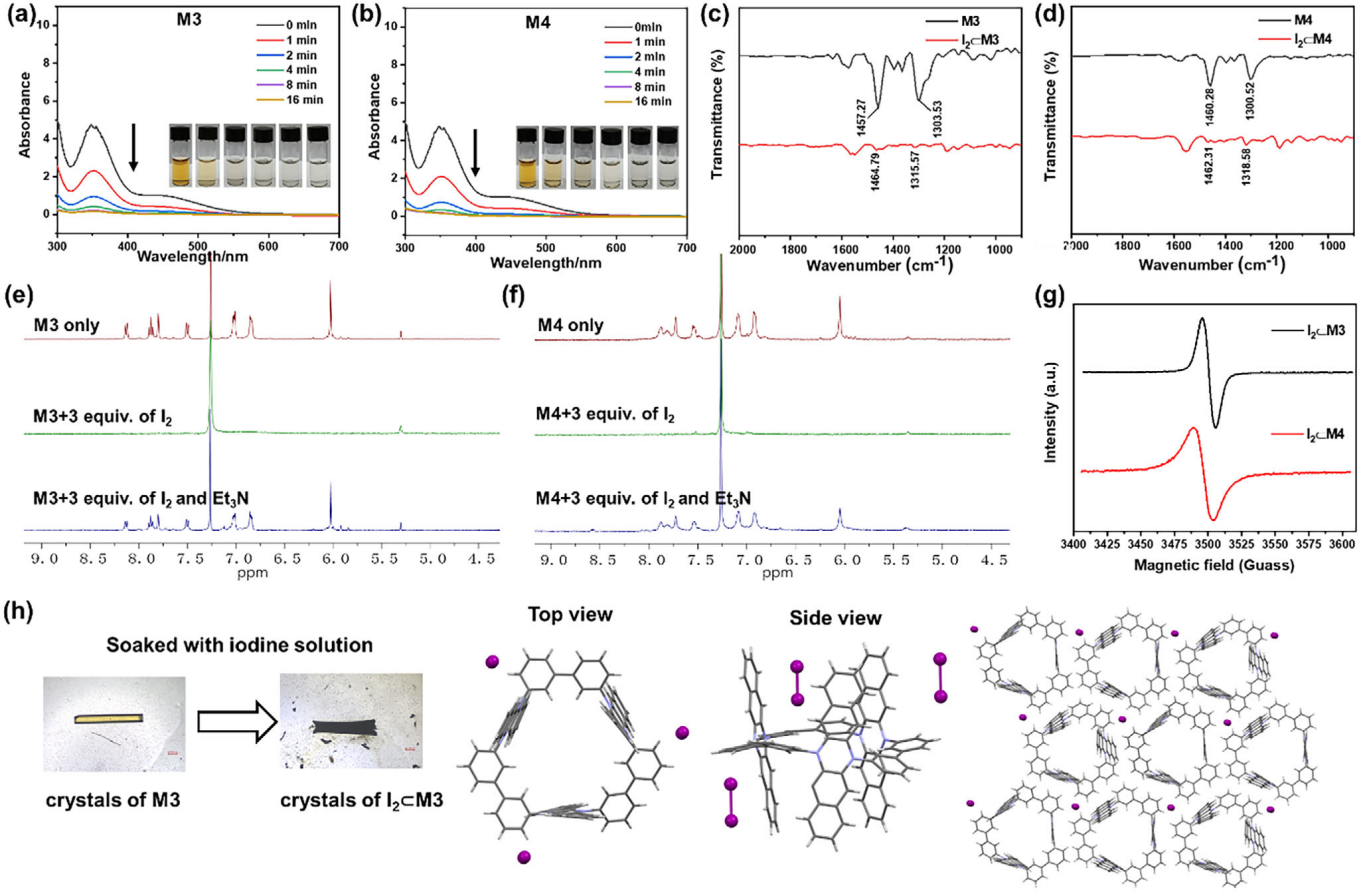
Characterization of the iodine adsorption. a) Time‐dependent UV–vis absorption spectra of saturated aqueous I_2_ solution upon addition of **M3** powder (3 mg). Insert is the color change of saturated aqueous I_2_ solution after the addition of **M3** powder. b) Time‐dependent UV–vis adsorption spectra of saturated aqueous I_2_ solution upon addition of **M4** powder (3 mg). Insert is the color change of saturated aqueous I_2_ solution after the addition of **M4** powder. c) FT‐IR spectra of **M3**, I_2_⊂**M3** and d) **M4**, I_2_⊂**M4**. e) ^1^H NMR spectra (400 MHz, CDCl_3_, 298 K) of pristine **M3**, I_2_⊂**M3** and I_2_⊂**M3** after adding triethylamine. f) ^1^H NMR spectra (400 MHz, CDCl_3_, 298 K) of pristine **M4**, I_2_⊂**M4** and I_2_⊂**M4** after adding triethylamine. g) EPR spectra of I_2_⊂**M3** and I_2_⊂**M4** in dichloromethane solution measured at room temperature. h) Side view and top view of the X‐ray crystal structures of I_2_⊂**M3** and illustration of the packing of I_2_⊂**M3** single crystals. C, gray; H, white; N, blue; I, purple. Solvent was omitted for clarity.

The iodine adsorption properties of two macrocycles were then investigated. With macrocyclic powder shaken in iodine aqueous solution, iodine aqueous solution changed from yellow to colorless within 4 min. The time‐dependent UV–vis curves of iodine in water dramatically decreased in 4 min at the presence of **M3** or **M4** (**Figure** **3**a,b; Figures S4 and S5, Supporting Information). Next, the uptake ability of iodine vapor was tested. **M3** and **M4** were placed in containers filled with iodine vapor at 25 °C respectively. After ≈150 h, the powder of **M3** and **M4** turned black with saturation adsorption capacity of 1.92 and 1.59 g g^−1^ respectively (Figure S3, Supporting Information). When the powder of I_2_⊂**M3** and I_2_⊂**M4** was immersed in methanol and stirred, the macrocycle‐adsorbed iodine exhibited a slow‐release behavior, which was monitored by UV–vis absorption spectra (Figures S6 and S7, Supporting Information). The iodine adsorption‐release experiments were repeated for 5 cycles, with no significant degradation observed in the macrocycles' adsorption capacity, indicating the exceptional recyclability (Figure S8, Supporting Information).

To further investigate the iodine adsorption mechanism of the macrocycles, we conducted a series of systematic experiments. Thermogravimetric (TG) analysis revealed that **M3** and **M4** exhibited excellent thermal stability, showing no significant mass loss when heated to 565 and 580 °C respectively (Figure S9, Supporting Information). For the iodine‐saturated samples of the macrocycles, a mass loss of 75% and 76% was observed under heating conditions (Figure S10, Supporting Information). These results ascribe to the release of iodine by weak physical adsorption in the sample.^[^ ^10^ ^]^ FT‐IR spectra of **M3** and **M4** changed greatly after adsorbing the iodine. For **M3** as an example, the C═C stretching vibration (1457 cm^−1^) in 6,13‐diazapentacene subunits disappeared after adsorbing the iodine (Figure 3c,d). These results indicate that the 6,13‐diazapentacene subunits exist oxidation states after the adsorption of iodine.^[^ ^18^, ^21^ ^]^ With the addition of iodine, the ^1^H NMR signals experienced significant paramagnetic line broadening due to the generation of radical species. Subsequently, upon the addition of triethylamine as a reducing agent, the signals returned to their original state, confirming that the radical macrocycles had reverted to their initial forms (Figure 3e,f; Figures S11 and S12, Supporting Information).^[^ ^45^, ^46^ ^]^ UV–vis absorption spectra similarly corroborated the formation of radical species upon the interaction of the macrocycles with iodine. Specifically, upon the addition of iodine, a new absorption peak at 580 nm was observed, accompanied by an EPR signal with no hyperfine splitting (Figures S14, S15, and S18, Supporting Information), indicating the potential generation of radical species (**M3** ^3•+^and **M4** ^4•+^).^[^ ^47^ ^]^ Similarly, when AgSbF_6_ was introduced as an oxidant, the same new absorption peak at 580 nm and an identical EPR signal appeared with no hyperfine splitting (Figures S16, S17, and S18, Supporting Information). This critical observation confirmed that iodine, like AgSbF_6_, effectively serves as an oxidant, generating the radical species of the macrocycles (**M3** ^3•+^and **M4** ^4•+^).^[^ ^47^ ^]^ Likewise, when excess reducing agent triethylamine was introduced, the characteristic absorption peaks of both systems at 580 nm disappeared and the solution color reverted from purple to its original yellow. These experimental results demonstrate that the macrocycles could be oxidized by both iodine and AgSbF_6_ to generate identical radical species, confirming the chemical adsorption of iodine by the macrocycles. To better demonstrate the interaction mechanism between the macrocycles and iodine, we initially attempted to grow single crystals by directly mixing the macrocycles with iodine in solution. However, this approach proved unsuccessful due to complications arising from the concomitant chemical oxidation process. Fortunately, we eventually succeeded in obtaining iodine‐adsorbed single crystals by immersing pre‐formed macrocyclic crystals in an iodine solution. Crystals of **M3** were soaked in a diethyl ether solution of iodine for a week to give complex I_2_⊂**M3** (Figure 3h).^[^ ^48^ ^]^ Specifically, upon immersion in an iodine‐ether solution, the initially bright yellow macrocyclic crystals turned black, and the width of a representative rectangular crystal increased from 200.52 to 272.41 µm, indicating a substantial incorporation of iodine or solvent molecules into the crystal lattice (Figure S19, Supporting Information). This observation suggests a potential charge‐transfer interaction or host‐guest complexation driven by the electrophilic character of iodine, leading to both chromatic and structural modifications. In the structure of I_2_⊂**M3**, iodine is preferentially accommodated within the interstitial voids of the macrocyclic crystal. This observation can be attributed to the crystallization methodology, where tight intermolecular stacking of the macrocycles sterically blocks cavity access, preventing iodine encapsulation in the cavity.

**Figure 4:**
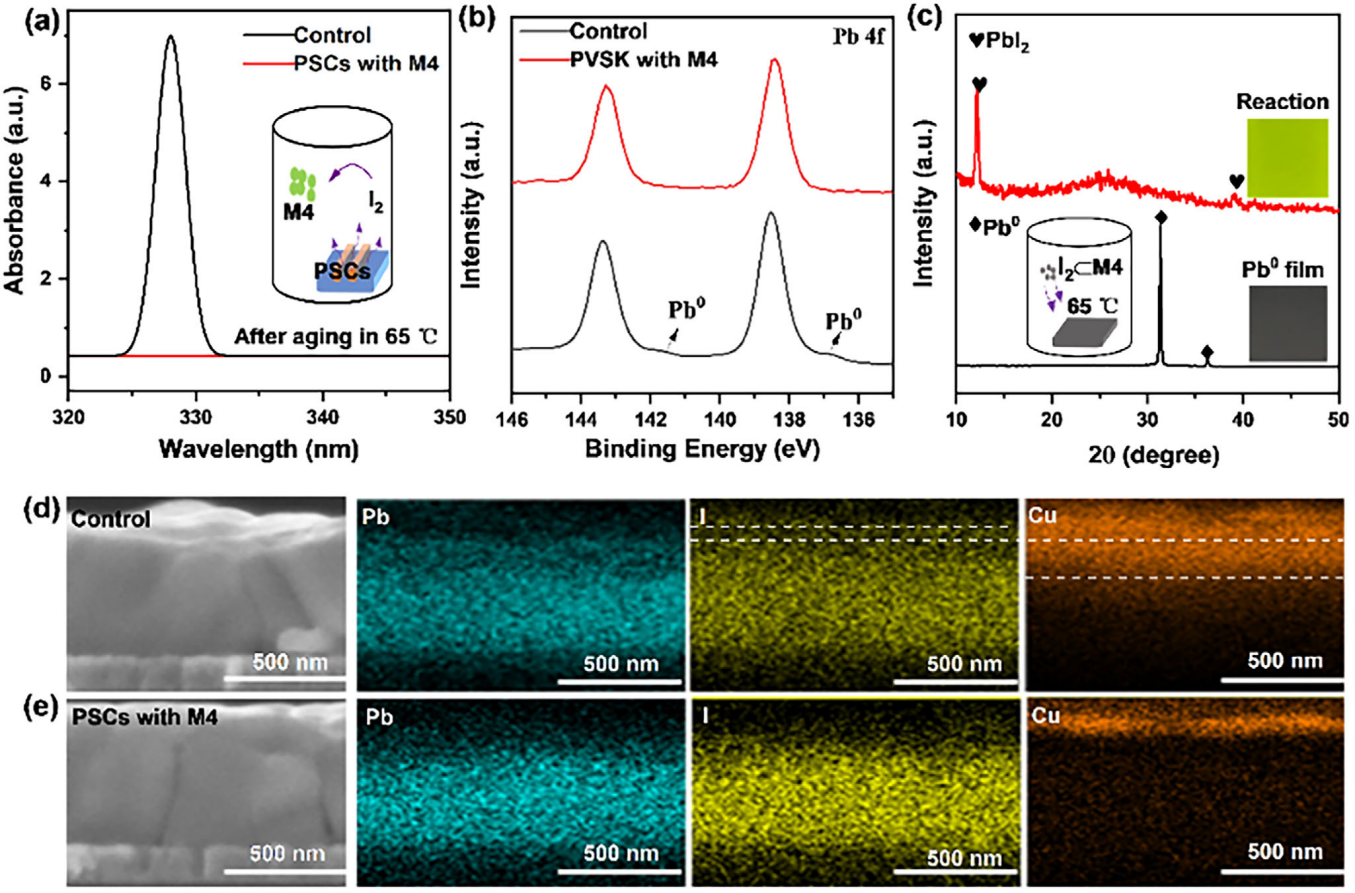
Effect of macrocyclic iodine adsorbent in perovskite. a) UV–vis absorption spectra of the resulting solution in iodine escaping experiment, where **M4** powder and real PSCs are put in sealed bottles and aged at 65 °C for 100 h. Then the powder is taken out and dissolved in dimethylformamide (DMF) to obtain the final solution. b) XPS spectra of Pb 4f in control perovskite film and perovskite film with **M4** after light‐heat aging. c) XRD patterns of metallic Pb^0^ film before and after storage with I_2_⊂**M4** at 65 °C in sealed bottles. d) Cross‐sectional EDX mapping of Pb, I, and Cu elements in aged control and e) PSCs with **M4**.

**Figure 5:**
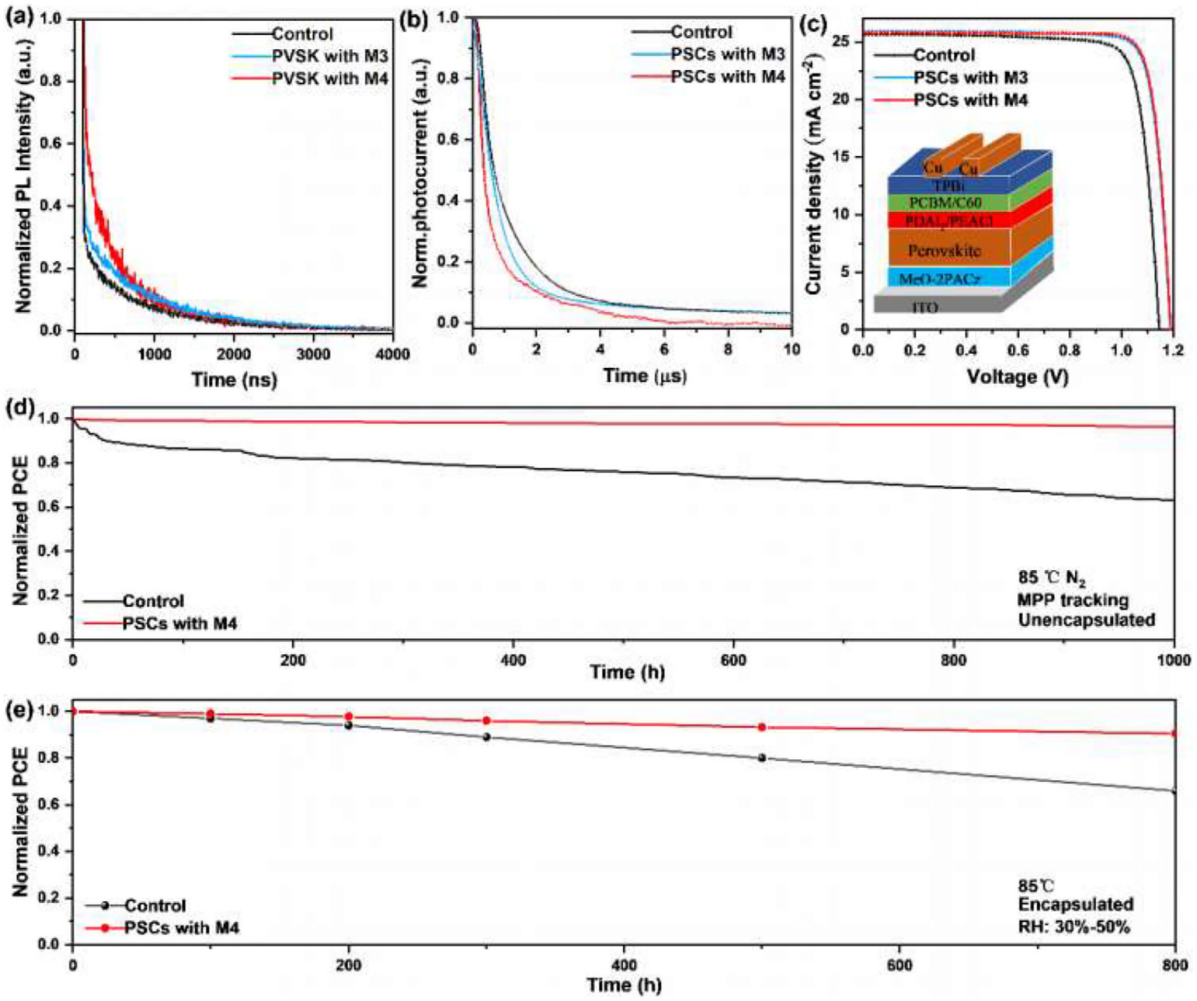
Device performance and stability. a) TRPL of control perovskite, perovskite with **M3** and perovskite with **M4**. b) TPC of control PSCs, PSCs with **M3**, and PSCs with **M4**. c) *J–V* curves of PSCs. d) MPP tracking of unencapsulated PSCs under continuous illumination at 85 °C. e) Humidity‐heat stability of encapsulated PSCs (85 °C, relative humidity≈30–50%).

When introducing **M3** and **M4** into perovskite, PSCs with **M4** showed better performance than that with **M3** (discussed in Figure 5). Therefore, **M4** was mainly used as a demo to investigate the effectiveness of macrocyclic iodine adsorbents in perovskite. As shown in **Figure** **4**a, an iodine escaping experiment was designed where perovskite films with **M4** were put into a sealed bottle and aged at 65 °C for 100 h. If iodine species indeed escape from perovskite during aging process and these escaped iodine species can be adsorbed by **M4**, the **M4** powder will contain iodine species, which will be detected by UV–vis absorption spectra after dissolving in dimethylformamide (DMF) solvent. Resulting solution from control perovskite showed obvious characteristic absorption peaks of iodine species at 327 nm (Figure 4a),^[^ ^49^, ^50^, ^51^ ^]^ indicating severe iodine loss and its adsorption by **M4**. While in perovskite with **M4**, almost no iodine absorption peak appeared, indicating the well‐inhibited iodine escaping. Besides, the disappearance of C═C characteristic FT‐IR peak in **M4** with control perovskite further indicated that the escaped iodine from perovskite could indeed be adsorbed by **M4** (Figure S20, Supporting Information). Generally, the iodine escaping from perovskite are accompanied by Pb^0^ formation,^[^ ^52^, ^53^ ^]^ which further induces deep defects and accelerates perovskite degradation. Control perovskite films showed obvious metallic Pb^0^ signal in X‐ray photoelectron spectroscopy (XPS) (Figure 4b) after aging for 100 h under light‐heat conditions, which could be further confirmed by X‐ray diffraction (XRD, Figure S21, Supporting Information). While in perovskite with **M4**, iodine escaping will be effectively inhibited due to the bonding between **M4** and iodine. Importantly, the iodine species captured by **M4** can also react with Pb^0^ to further inhibit the formation of Pb^0^ defects, which is beneficial to perovskite stability.^[^ ^54^ ^]^ As shown in Figure 4c, the gray metallic Pb^0^ film would convert to yellow PbI_2_ when putting I_2_⊂**M4** adsorption product and metallic Pb^0^ film together and heating at 65 °C. This result confirms that iodine adsorbed by **M4** can be released to inhibit the generation of Pb^0^ defects by converting Pb^0^ to PbI_2_. Cross‐sectional scanning electron microscopy with energy‐dispersive X‐ray (SEM‐EDX) was conducted to study the iodine escaping in real PSCs during long‐term aging (fresh perovskite morphology in Figure S22, Supporting Information). As shown in Figure 4d, iodine elements distribution had surpassed the regions of Pb elements, indicating inevitable iodine migration and escaping during device aging. Besides, the escaped iodine species from perovskite will diffuse toward and further react Cu metal electrode, inducing electrode corrosion and accelerating Cu element diffusion in control PSCs.^[^ ^30^, ^55^ ^]^ While in PSCs with **M4**, iodine escaping was effectively inhibited owing to **M4** adsorption or confining function, and almost no iodine or Cu diffusion was observed (Figure 4e).

Besides stability, perovskite with **M4** showed much longer carriers lifetime of 1017 ns, which was better than control perovskite (679 ns) and perovskite with **M3** (929 ns) as confirmed in time‐resolved photoluminescence (TRPL) (**Figure** **5**a; Fitting data in Table S3; Photoluminescence of PL in Figure S23, Supporting Information). PSCs with **M4** showed much shorter photocurrent decay lifetime of 0.51 µs than control (1.01 µs) and **M3**‐based devices (0.77 µs, transient photocurrent of TPC in Figure 5b), indicating faster carrier extraction. Additionally, PSCs with **M4** also showed obviously longer photovoltage decay lifetime (1.07 µs) than control (0.61 µs) and **M3**‐based devices (0.96 µs, transient photovoltage test in Figure S24, Supporting Information), indicating the inhibited trap‐assisted carrier recombination. These results indicate that the introduction of **M4** adsorbents can improve carriers transport in perovskite films, which is beneficial to finial device performance.

Inverted PSCs were fabricated with configuration of ITO/MeO‐2PACz/Perovskite/PDAI_2_/PEACl/PCBM/C60/TPBi/Cu (Figure 5c inset). As shown in Figure 5c, when introducing **M4** adsorbents, device efficiency would be greatly improved from initial 24.07% (control) to 26.13% (*J–V* hysteresis in Figure S25, concentration optimization in Figure S26, Supporting Information), which was even higher than **M3**‐based devices (25.88%). The improved performance was mainly due to the enhanced FF (81.84% vs 85.07%) and Voc (1.147 V vs 1.190 V). The stabilized power output of PSCs with **M4** reached 26.01% (Figure S27, Supporting Information). In addition, PSCs with **M4** also exhibited good reproducibility and the average PCE reached 26.02% among 10 separated devices (Figure S28, Supporting Information). The integrated current from external quantum efficiency (EQE) was 25.19 mA cm^−2^ in PSCs with **M4**, agreeing well with the *J–V* test (≈3% mismatch, Figure S29, Supporting Information).

Long‐term operational stability is also an important issue for PSCs due to its self‐degradation process.^[^ ^56^ ^]^ In general, perovskite tends to decompose and generates volatile I_2_ species under illumination. Once increasing working temperature, I_2_ species will escape from perovskite, destroying the decomposing equilibrium and thus accelerating perovskite degradation.^[^ ^57^ ^]^ While in PSCs with **M4**, I_2_ escaping from perovskite will be effectively inhibited through physical and chemical adsorption, thus suppressing perovskite decomposing and enhancing device stability. Resulting unencapsulated PSCs with **M4** retained 95.85% of initial efficiency after MPP tracking for 1000 h under continuous illumination at 85 °C with ISOS‐L‐2 protocol. While control PSCs degraded rapidly and retained 62.97% of initial efficiency under those same conditions (Figure 5d). In addition, PSCs with **M4** also exhibited excellent humidity‐heat stability, retaining 90.54% of initial efficiency at 85 °C for 800 h in air (30 to 50% relative humidity (RH), with encapsulation), whereas only 66.01% of the initial efficiency was retained in control PSCs (Figure 5e). Electrical impedance spectroscopy (EIS) is conducted to assess device's stability. As shown in Figure S30 (Supporting Information), after MPP tracking for 1000 h at 85 °C, the control PSCs shows increased resistance (*R_s_*_,_ 21 Ω vs 29  Ω) and reduced significantly recombination resistance (*R_rec_*, 4128 Ω vs 1931  Ω), indicating the control PSCs has undergone severe degradation. While in PSCs with **M4**, since **M4** demonstrates good adsorption‐desorption recyclability, allowing the release of captured iodine and promoting perovskite regeneration, the *R_s_* (18 Ω vs 22  Ω) and *R_rec_* (4387 Ω vs 3565  Ω) change slightly, which is consistent with the results of MPP tracking.

## 3 Conclusion

In summary, we have developed a new class of conjugated macrocycles, **M3** and **M4**, based on diazapentacene units, and demonstrated their potential for enhancing the stability of perovskite solar cells by capturing dynamically released iodine and mitigating iodide loss. The incorporation of electron‐rich, π‐conjugated diazapentacene moieties endows the macrocycles with redox activity and well‐defined cylindrical cavities, enabling both physical confinement and chemical adsorption of electron‐deficient iodine species. These dual‐mode adsorption capabilities make **M3** and **M4** effective iodine adsorbents. In particular, macrocycle **M4** efficiently suppresses iodine volatilization during perovskite degradation and promotes self‐healing of Pb^0^ defects via iodine recapture. As a result, PSCs incorporating **M4** exhibit high power conversion efficiency of 26.13% and outstanding operational stability, retaining ≈95.85% of their initial efficiency after 1000 h of MPP tracking at 85  °C under the ISOS‐L‐2 protocol. This study highlights the utility of supramolecular macrocycles in PSC stabilization and provides valuable insights for the broader design of functional macrocyclic and supramolecular systems for advanced energy materials.

## 4 Experimental Section

### Characterization and Measurements

All reagents and starting materials were obtained from commercial suppliers and used without further purification. All air‐sensitive reactions were carried out under inert N_2_ atmosphere. 6,13‐dihydro‐6,13‐diazapentacene **1** was prepared following the reported literature.^[^ ^58^ ^]^ The ^1^H NMR and ^13^C NMR spectra were obtained using the Bruker 400 MHz spectrometer. Moreover, tetramethylsilane (TMS) or solvent peaks were used as an internal standard. Multiplicities are denoted as follows: s = singlet, d = doublet, t = triplet, m = multiplet. The HR‐ESI‐MS mass spectra were performed on Q Exactive Focus (Thermo Scientific, USA). High‐resolution mass spectra (HRMS) were obtained on a GCT premier CAB048 mass spectrometer operating in a MALDI‐TOF mode. The single crystals were measured on Rigaku XtaLAB PRO MM003‐DS dual system with a Cu microfocus source (λ = 1.54184 Å) and BL17B beamline at Shanghai Synchrotron Radiation. UV–vis spectra were recorded in a quartz cell (light path 10 mm) on a Shimadzu UV2700 UV–vis spectrophotometer. Thermogravimetric analysis (TGA) was performed on a ZRT‐A analyzer over a temperature range of 30–820 °C with a heating rate of 10 °C min^−1^. Fourier transform infrared (FT‐IR) spectra were recorded on a Bruker Tensor 37. Spectrometer. Electron paramagnetic resonance (EPR) spectra for radicals were obtained on Bruker EMX instrument EMXPLUS‐10/12 spectrometer. Cyclic voltammetry (CV) was recorded on a Bio‐Logic SAS SP‐150 spectrometer in dichloromethane containing *n*‐Bu_4_NPF_6_ (0.1 m) as supporting electrolyte at a scan rate of 20 mV s^−1^ at room temperature. The CV cell has a glassy carbon electrode, a Pt wire counter electrode, and an Ag/Ag^+^ reference electrode. The potential was calibrated against the ferrocenium/ferrocene (Fc^+^/Fc) couple. Gel permeation chromatography was performed at room temperature using dichloromethane as solvent on a recycling preparative gel permeation chromatography (GPC): JAI LaboACE LC‐5060 series (Japan Analytical Industry Co., Ltd.) equipped with a pump (P‐LA60, flow rate 10 mL min^−1^), a UV detector (UV–VIS4ch LA, λ = 254, 300, 400 nm, and 500 nm), and two columns (JAIGEL 2HR and 2.5HR).

The *J–V* curves were obtained under a solar simulator (Enlitech) using a Keithley 2400 digital source meter under simulated AM 1.5G illumination (100 mW cm^−2^). The *J–V* curves were all measured at a scan rate of 0.015 V s^−1^ under a forward scan from 1.25 to −0.2 V in a glove box at room temperature. The EQE measurement was carried out in ambient air using a QE system (SOFN Instruments Co., ltd) with monochromatic light focused on a device pixel. X‐ray photoelectron spectroscopy (XPS) measurement was carried out using Kratos AXIS ULTRA DALD XPS system. Scanning electron microscope (SEM) images and EDX were recorded using field emission scanning electron microscope (Zeiss GeminiSEM450) with an accelerating voltage of 10 kV. Transient photovoltage (TPV) and transient photocurrents (TPC) were obtained on an all‐in‐one characterization platform paios developed by Fluxim AG, Switzerland in the laboratory of Zhipeng Kan at Guangxi University. The AM 1.5G illumination is achieved with a Xenon lamp. XRD spectra were recorded in an Empyrean Micro diffractometer with Cu Kα radiation. The PL and TRPL decay were conducted using a FLS 1000 fluorescence spectrometer. The films under investigation were prepared on glass substrates, with an excitation wavelength of 460 nm employed for these analyses.

### PSCs Materials

Lead (II) iodide (PbI_2_), lead (II) chloride (PbCl_2_), cesium iodide (CsI), methylammonium iodide (MAI), methylammonium chloride (MACl), formamidinium iodide (FAI), 1,3,5‐tris(1‐phenyl‐1H‐benzimidazol‐2‐yl)benzene (TPBi), 2‐phenylethylammonium chloride (PEACl), 6,6‐phenyl‐C61‐butyric acid methyl ester (PCBM) and C60 were purchased from Xi'an Polymer Light Technology Corp. Isopropanol (IPA), chlorobenzene (CB), dimethylformamide (DMF) and dimethyl sulfoxide (DMSO) were bought from Sigma‐Aldrich. [2‐(3,6‐Dimethyl‐9H‐carbazol‐9‐yl)butyl]phosphonic acid (MeO‐2PACz) and 1,3‐diaminopropane dihydroiodide (PDAI_2_) were bought from TCI.

### Device Fabrication

ITO substrates (20 mm × 20 mm) were cleaned with detergent, distilled water, acetone and isopropanol. The cleaned substrates were dried using N_2_ flow and treated with UV‐Ozone for 20 min in air. MeO‐2PACz solution (Ethanol, 2 mg mL^−1^) was deposited on the ITO substrate by spin‐coating at 3000 rpm for 30 s and then annealed at 100 °C for 10 min. 1.3 m Cs_0.05_FA_0.9_MA_0.05_PbI_3_ perovskite precursor solution in DMF: DMSO (4:1) solvent was spin‐coated on MeO‐2PACz at 2000 rpm for 10 s and 4000 rpm for the 20 s. During spin‐coating, 150 µL chlorobenzene (CB) was dropped on the substrate 12 s before the end of the second program of 4000 rpm for 20 s. For the preparation of modified films, 1 mg mL^−1^ **M3** and **M4** were added into perovskite precursor solution. All perovskite films are annealed at 120 °C for 20 min (N_2_ atmosphere). PDAI_2_ (0.5 mg mL^−1^) and PEACl (2 mg mL^−1^) were deposited separately layer by layer on the perovskite layer at 4000 rpm for 30 s and then annealed at 100 °C for 10 min. PCBM was deposited on the passivation layer at 2000 rpm for 45 s. The substrate was then transferred to a vacuum chamber. C60 (40 nm, 0.4–0.5 Ǻ s^−1^), TPBi (8 nm, 0.4–0.5 Ǻ s^−1^), and Cu (100 nm, 0.8–1.0 Ǻ s^−1^) were deposited sequentially by thermal evaporation. The device area is 0.09 cm^2^.

### Stability Device Fabrication

P3CT‐N solution (1 mg mL^−1^ in methanol) was deposited on the ITO substrate by spin‐coating at 4000 rpm for 30 s and then annealed at 100 °C for 10 min. 1.5 m Cs_0.05_FA_0.95_PbI_3_ perovskite precursor solution contained 15% MACl and 5% PbCl_2_ in DMF: DMSO (4:1) solvent was spin‐coated on P3CT‐N at 2000 rpm for 10 s and 4000 rpm for the 20 s. During spin‐coating, 150 µL CB was dropped on the substrate 10 s before the end of the second program of 4000 rpm for 20 s. For the preparation of modified films, **M4** was added into perovskite precursor solution. Then the substrate was annealed at 120 °C for 30 min. PDAI_2_ was deposited on the perovskite layer at 4000 rpm for 30 s and then annealed at 100 °C for 10 min. Subsequently, C60 (50 nm), ALD‐SnOx (250 cycles), and Cu (100 nm) were deposited. The device area is 0.09 cm^2^.

### Stability Testing

The unencapsulated device was tested in N_2_ atmosphere under continuous illumination (white LED) at 85 °C. *J–V* curves were automatically measured every 3 h. Operational stability was recorded with a 16‐channel MPP measuring system under continuous illumination (white LED) at 85 °C. During the MPP tracking, the light intensity could be automatically calibrated with a Si reference diode. To calibrate the initial illumination intensity to 100 mW cm^−2^, the PSC was first measured under the solar simulator (Enlitech, SS‐F5‐3A) with simulated AM 1.5G illumination to obtain a *J_sc_*. Then, the PSC was measured again under the white LED lamp to reach the same *J_sc_* through regulating the intensity of the LED lamp.

The X‐ray crystallographic coordinates for structures reported in this study have been deposited at the Cambridge Crystallographic Data Centre (CCDC), under deposition numbers CCDC 2463878 (**M3**), 2463874 (**M4**), 2465442 (**I_2_⊂M3**). These data can be obtained free of charge from The Cambridge Crystallographic Data Centre via www.ccdc.cam.ac.uk/data_request/cif. Source data are provided with this paper.

## Conflict of Interest

The authors declare no conflict of interest.

## Author Contributions

Y.W. and W.L. contributed equally to this work. X.S., X.L., J.F., X.‐L.Z., H.‐B.Y., Y.W., and W.L. conceived the project, analyzed the data, and wrote the manuscript. Y.W. and W.L. performed most of the experiments. S.L. helped with the sample preparation, functional experiments, and data collection. Y.‐F.N. and X.‐L.Z. conducted single crystal analyses. C.W. conducted mass spectrometry analysis. All authors discussed the results and commented on the manuscript.

## Supporting information



Supporting Information

Supplemental DataFile

## Data Availability

The data that support the findings of this study are available in the supplementary material of this article.
