# Supplementary material for: Supramolecular Macrocyclic Iodine Adsorbents Enable Photothermally Stable Perovskite Solar Cells
Source: Adv Sci (Weinh). 2025 Oct 30;13(4):e16964. doi: 10.1002/advs.202516964 (PMC12822466; doi:10.1002/advs.202516964)
Supplement: Supplementary file 1 — Supporting Information [file ADVS-13-e16964-s002.docx]

Supporting Information

Supramolecular Macrocyclic Iodine Adsorbents Enable Photothermally Stable Perovskite Solar Cells

Yue Wu^1,3^, Wen Li^2,3^, Shengzhong Li^1^, Yan-Fei Niu^1^, Cuihong Wang^1^, Hai-Bo Yang^1^, Xiao-Li Zhao^1*^, Xiaodong Li^2*^, Junfeng Fang^2*^, Xueliang Shi^1*^

^1^State Key Laboratory of Petroleum Molecular & Process Engineering, Shanghai Key Laboratory of Green Chemistry and Chemical Processes, School of Chemistry and Molecular Engineering, East China Normal University, Shanghai 200062, China.

^2^School of Physics and Electronic Science, Engineering Research Center of Nanophotonics & Advanced Instrument, Ministry of Education, East China Normal University, Shanghai 200062, China.

^3^These authors contributed equally to this work.

^*^E-mail: [xlzhao@chem.ecnu.edu.cn](mailto:xlzhao@chem.ecnu.edu.cn), [xdli@phy.ecnu.edu.cn](mailto:xdli@phy.ecnu.edu.cn), [jffang@phy.ecnu.edu.cn](mailto:jffang@phy.ecnu.edu.cn), [xlshi@chem.ecnu.edu.cn](mailto:xlshi@chem.ecnu.edu.cn)

## Synthesis


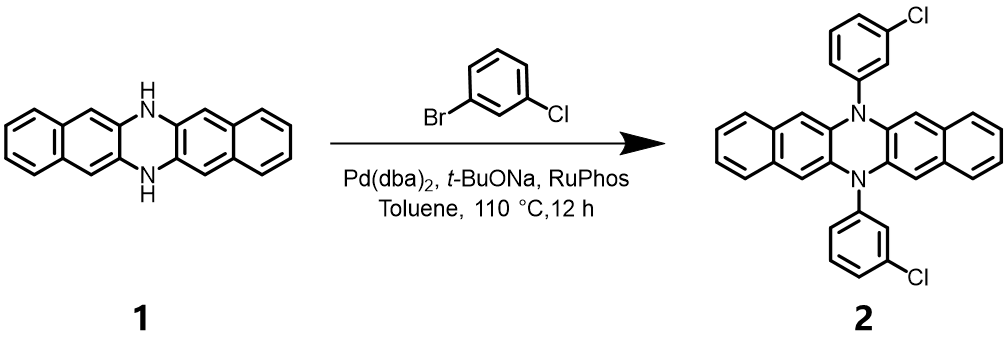


**Figure S1.** Synthesis of **2.**

In a 250 mL Schlenk flask, 4-Bromochlorobenzene (1.92 g, 10.02 mmol), *t-*BuONa (962.93 mg, 10.02 mmol), RuPhos (116.75 mg, 0.25 mmol), and Pd_2_(dba)_3_ (119.08 mg, 0.13 mmol) was mixed and dissolved in 40 mL toluene. Under a N_2_ atmosphere, 6,13-dihydro-6,13-diazapentacene **1** (707.62 mg, 2.51 mmol) were added. After stirring for 12 h at 110 °C, the mixture was cooled down to room temperature and 200 mL of dichloromethane were added. The mixture was washed three times with 200 mL of H_2_O and dried over Na_2_SO_4_. After filtration the mixture was adsorbed on silica and purified by column flash chromatography with a solvent mixture of petroleum ether and dichloromethane (4:1). Compound **2** (1.07 g, 2.13 mmol) was obtained as a yellow powder in 85% yield. ^1^H NMR (400 MHz, CDCl_3_, 298 K) δ (ppm) 7.71 (t, J = 8.0 Hz, 2H), 7.63 (d, J = 8.0 Hz, 2H), 7.53 (s, 2H), 7.43 (d, J = 8.0 Hz, 2H), 7.18 (dd, J = 6.0, 3.1 Hz, 4H), 7.04 (dd, J = 6.0, 3.1 Hz, 4H), 6.01 (s, 4H). ^13^C NMR (101 MHz, CDCl_3_, 298 K) δ (ppm) 140.9, 137.0, 134.6, 132.8, 131.4, 130.1, 129.6, 129.5, 125.9, 124.3, 108.3. HR-ESI-MS (m/z): M^•+^ calculated for C_32_H_20_N_2_Cl_2_ 502.1004, found 502.0988.

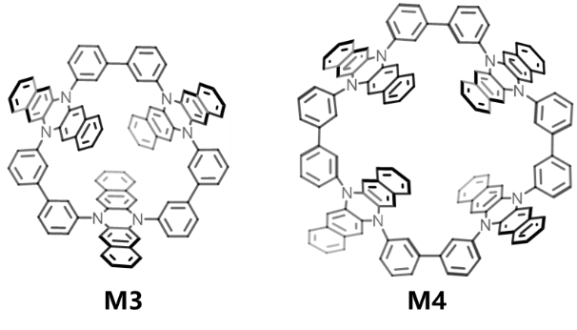


**Figure S2.** Synthesis of **M3**, **M4**.

Ni(cod)_2_ (1 g, 3.64 mmol), cod (630.04 mg, 5.82 mmol), 2, 2’-bipyridyl (726.96 mg, 4.66 mmol), and dry toluene (200 mL) were added to a 500 mL Schlenk flask filled with N_2_ and then the mixture was stirred at 85 °C for 1 h. After an hour, the mixture was cooled down to 65 °C. Then, compound **2** (735.01 mg, 1.46 mmol) dissolved in dry dimethylformamide (150 mL) was added into the Schlenk flask slowly and the mixture was stirred for another 24 h. After the reaction was quenched with H_2_O, the mixture was cooled down to room temperature and 500 mL of ethyl acetate were added. The mixture was washed three times with 200 mL of H_2_O and dried over Na_2_SO_4_. The crude product was purified by silica-gel column chromatography (petroleum ether and dichloromethane (2:1)) and gel permeation chromatography (dichloromethane) to give **M3** as a yellow solid (49.23 mg, 0.038 mmol, 18% yield) and **M4** as a yellow solid (54.71 mg, 0.032 mmol, 15% yield). **M3**: ^1^H NMR (400 MHz, CDCl_3_, 298 K) δ (ppm) 8.14 (d, *J* = 7.8 Hz, 2H), 7.88 (t, *J* = 7.8 Hz, 2H), 7.81 (s, 2H), 7.51 (d, *J* = 7.8 Hz, 2H), 7.03 (dd, *J* = 6.1, 3.2 Hz, 4H), 6.85 (dd, *J* = 6.1, 3.2 Hz, 4H), 6.03 (s, 4H). ^13^C NMR (125 MHz, CD_2_Cl_2_, 298K) δ (ppm) 144.0, 142.1, 136.6, 134, 132.2, 131.9, 130.2, 128.1, 127.4, 125.6, 109.6. HR-ESI-MS (m/z): [M+H]^+^ calculated for C_96_H_61_N_6_ 1297.4952, found 1297.4842. **M4**: ^1^H NMR (400 MHz, CDCl_3_, 298 K) δ (ppm) 7.89-7.87 (m, 2H), 7.82-7.80 (m, 2H), 7.73 (s, 2H), 7.55 (d, *J* = 7.1 Hz, 2H), 7.09 (dd, *J* = 6.0, 3.0 Hz, 4H), 6.92 (dd, *J* = 6.0, 3.0 Hz, 4H), 6.05 (s, 4H). HR-ESI-MS (m/z): [M+H]^+^ calculated for C_128_H_81_N_8_ 1729.6539, found 1729.6581.

## 2. Iodine adsorption study of the macrocycle

Time-dependent iodine vapor uptake experiment was carried out in the following procedure. The power of **M3** or **M4** (10 mg) was added in glass vessel (4 mL). And then the glass vessel containing power was transferred in bigger sealed glass vial (20 mL) containing iodine (500 mg). The sample was kept at room temperature, and the mass of the small glass vessel (4 mL) was measured at regular intervals using an analytical balance. The iodine uptake capacities were calculated by weight gains: C=(W_b_-W_a_)/W_b_×100 wt %, where C is the iodine uptake capacity and W_a_ and W_b_ are the mass weight of the power before and after iodine vapor adsorption^[1]^.

**
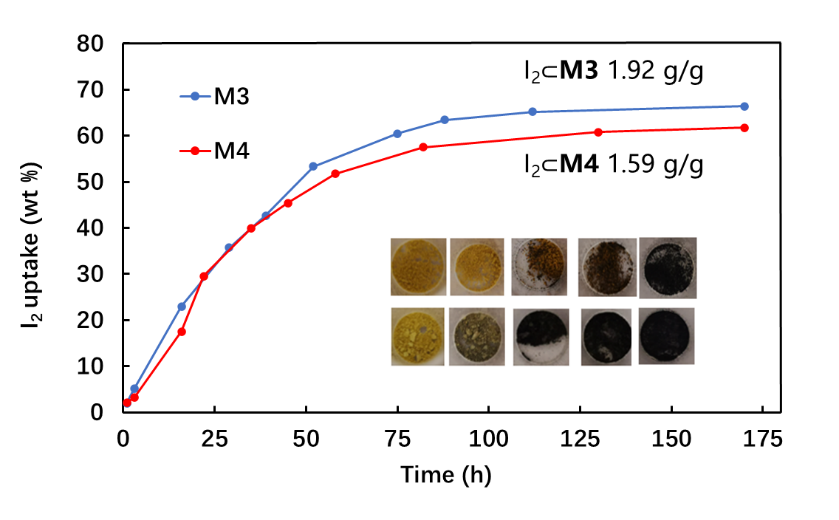
**

**Figure S3.** Time-dependent I_2_ vapor uptake profiles of **M3** and **M4** at room temperature respectively. Inserts are the photographs of I_2_ adsorption of **M3** (top) and **M4** (bottom) at room temperature respectively.

**Table S1.** The mass changes of **M3** under I_2_ vapor at room temperature.

| 25 °C **M3**: 10 mg **M3**+4 mL glass vessel: 4625.29 mg | | | |
| --- | --- | --- | --- |
| Time (h) | Total mass (mg) | Increased mass (mg) | C (wt %) |
| 1 | 4625.49 | +0.20 | 1.96 |
| 3 | 4625.83 | +0.54 | 5.12 |
| 16 | 4628.28 | +2.99 | 23.02 |
| 29 | 4630.84 | +5.55 | 35.69 |
| 39 | 4632.74 | +7.45 | 42.69 |
| 52 | 4636.73 | +11.44 | 53.36 |
| 75 | 4640.59 | +15.30 | 60.47 |
| 88 | 4642.62 | +17.33 | 63.41 |
| 112 | 4644.02 | +18.73 | 65.19 |
| 170 | 4645.08 | +19.79 | 66.43 |

**Table S2.** The mass changes of **M4** under I_2_ vapor at room temperature.

| 25 °C **M4**: 10 mg **M4**+4 mL glass vessel: 4761.35 mg | | | |
| --- | --- | --- | --- |
| Time (h) | Total mass (mg) | Increased mass (mg) | C (wt %) |
| 1 | 4761.57 | +0.22 | 2.15 |
| 3 | 4761.68 | +0.33 | 3.19 |
| 16 | 4763.48 | +2.13 | 17.56 |
| 22 | 4765.55 | +4.20 | 29.58 |
| 35 | 4768.00 | +6.65 | 39.94 |
| 45 | 4769.68 | +8.33 | 45.44 |
| 58 | 4772.07 | +10.72 | 51.74 |
| 82 | 4775.07 | +13.72 | 57.84 |
| 130 | 4776.85 | +15.50 | 60.78 |
| 170 | 4777.52 | +16.17 | 61.79 |

In order to monitor the iodine capture speed of **M3** and **M4** in aqueous solution, a time-dependent UV–vis measurement was carried out. The power of **M3** or **M4** (3.0 mg) was added in saturated aqueous I_2_ solution with shaking. The UV–vis absorption spectra of the solution were recorded over time^[1]^.


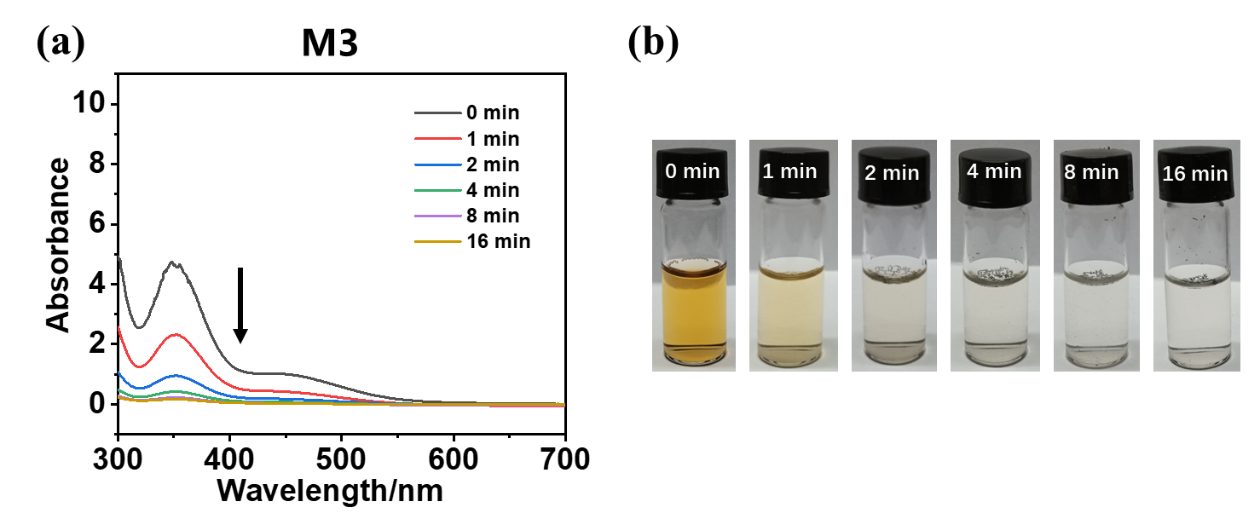


**Figure S4.** (a) Time-dependent UV–vis absorption spectra of solution of saturated aqueous I_2_ solution (2 mL) upon addition of **M3** powder (3 mg). (b) Color change of saturated aqueous I_2_ solution (2 mL) after the addition of **M3** powder.


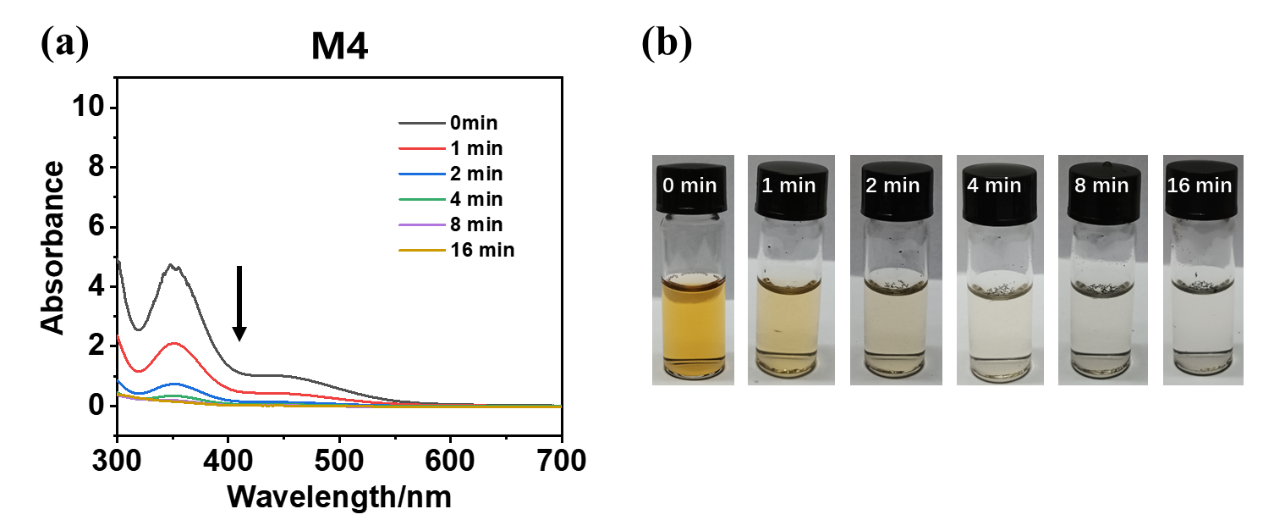


**Figure S5.** (a) Time-dependent UV–vis absorption spectra of solution of saturated aqueous I_2_ solution (2 mL) upon addition of **M4** powder (3 mg). (b) Color change of saturated aqueous I_2_ solution (2 mL) after the addition of **M4** powder.

In order to monitor the iodine release in a solvent, time-dependent UV–vis measurements were carried out in methanol. The power of I_2_⊂**M3** or I_2_⊂**M4** (2.0 mg) was added to methanol (5 mL). The UV–vis absorption spectra of the solution were recorded over time^[1]^.

**
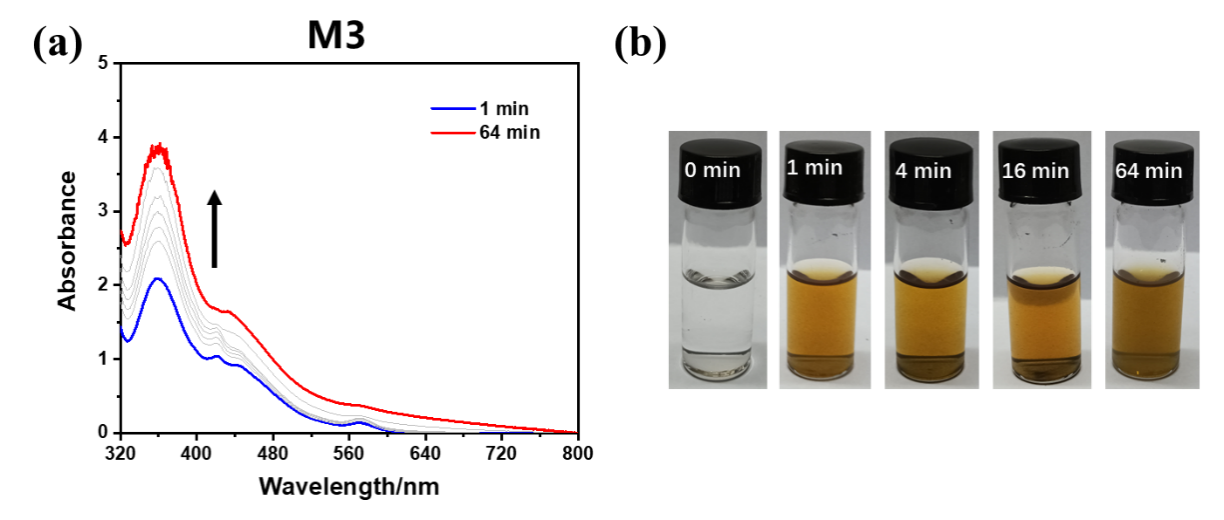
**

**Figure S6.** Time-dependent UV–vis absorption spectra of I_2_ desorption from I_2_⊂**M3** (2 mg) in methanol (5 mL). (b) Photographs of I_2_ desorption from I_2_⊂**M3** in methanol.

**
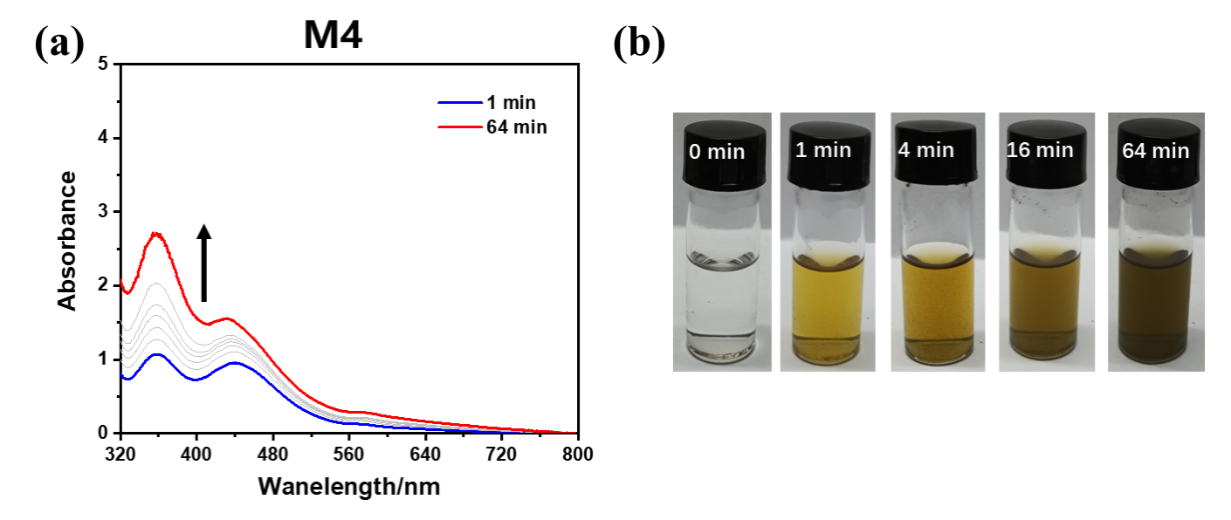
**

**Figure S7.** Time-dependent UV–vis absorption spectra of I_2_ desorption from I_2_⊂**M4** (2 mg) in methanol (5 mL). (b) Photographs of I_2_ desorption from I_2_⊂**M4** in methanol.

The iodine-loaded macrocyclic sample was treated with methanol containing a small amount of triethylamine and shaken until the methanol phase became colorless. After solvent removal, the washed powder was vacuum-dried for 10 h, with sample masses recorded both before and after the washing process^[2]^.

**
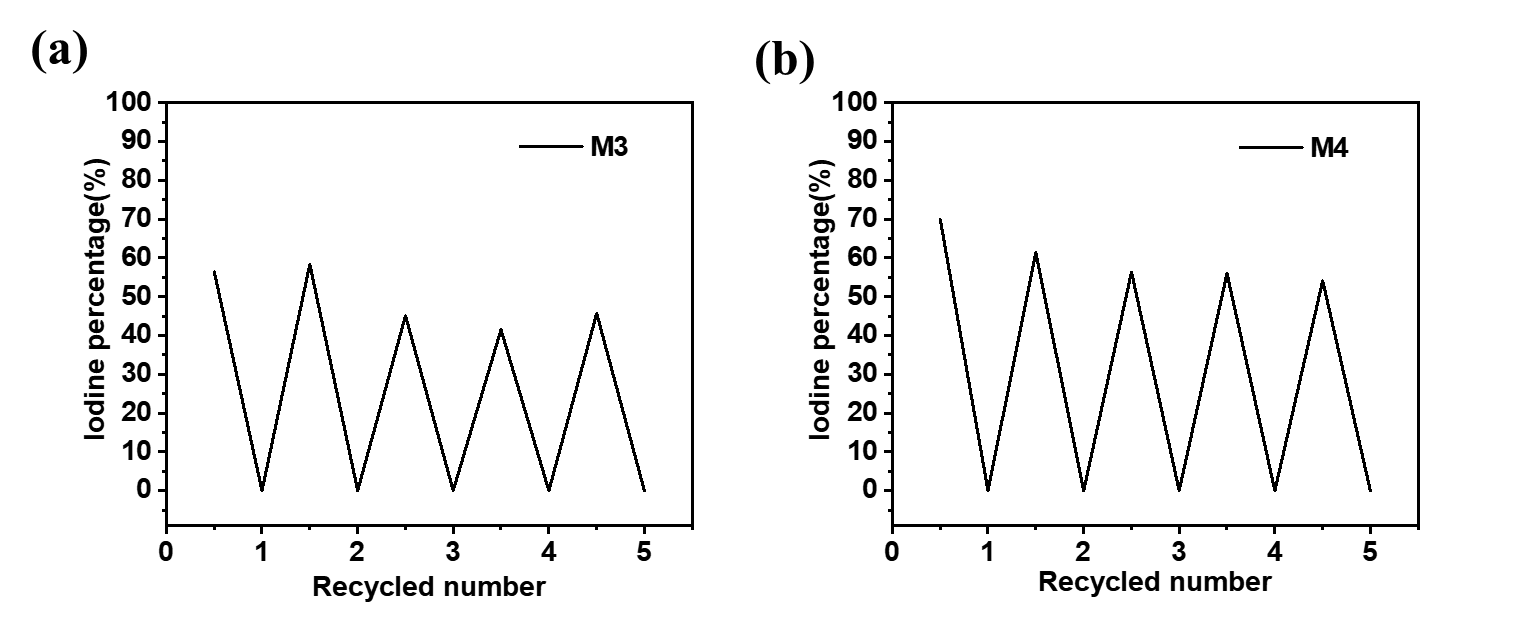
**

**Figure S8.** I_2_ adsorption/desorption cycles of (a) **M3** and (b) **M4**

**
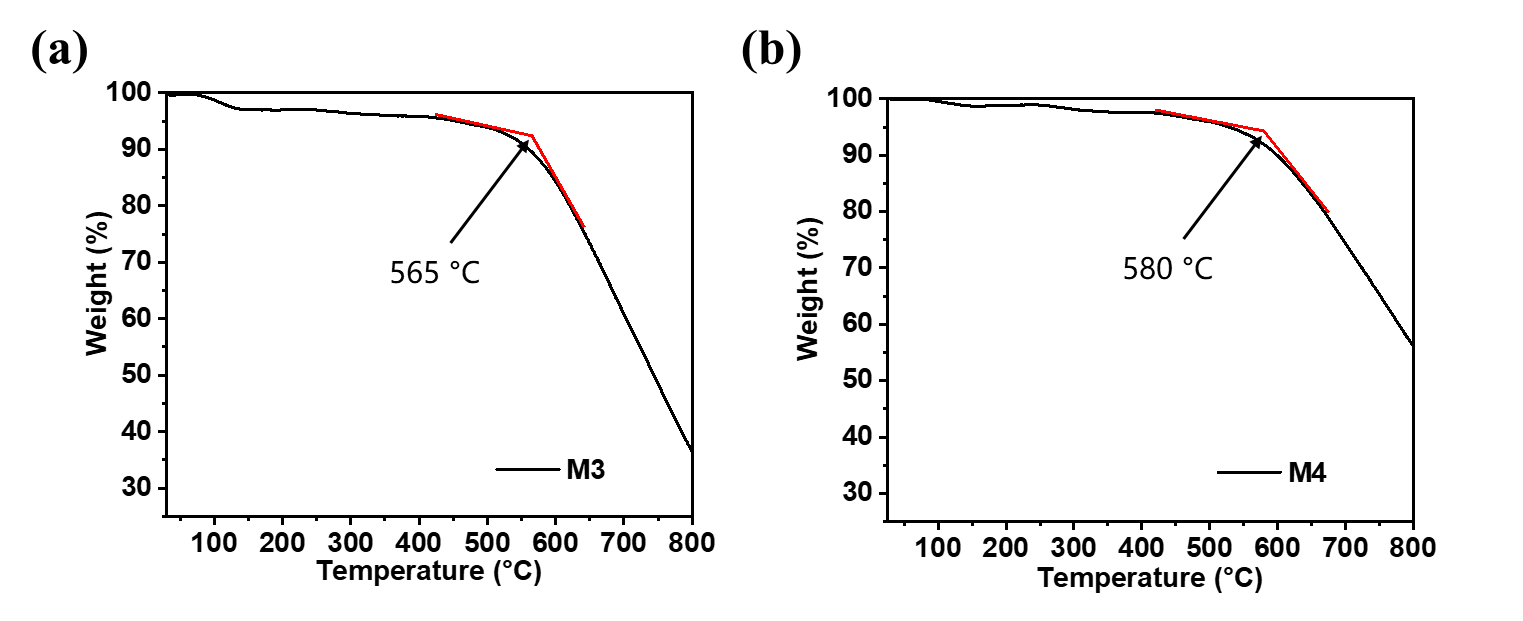
**

**Figure S9.** Thermogravimetric analysis of (a) **M3** and (b)**M4**.


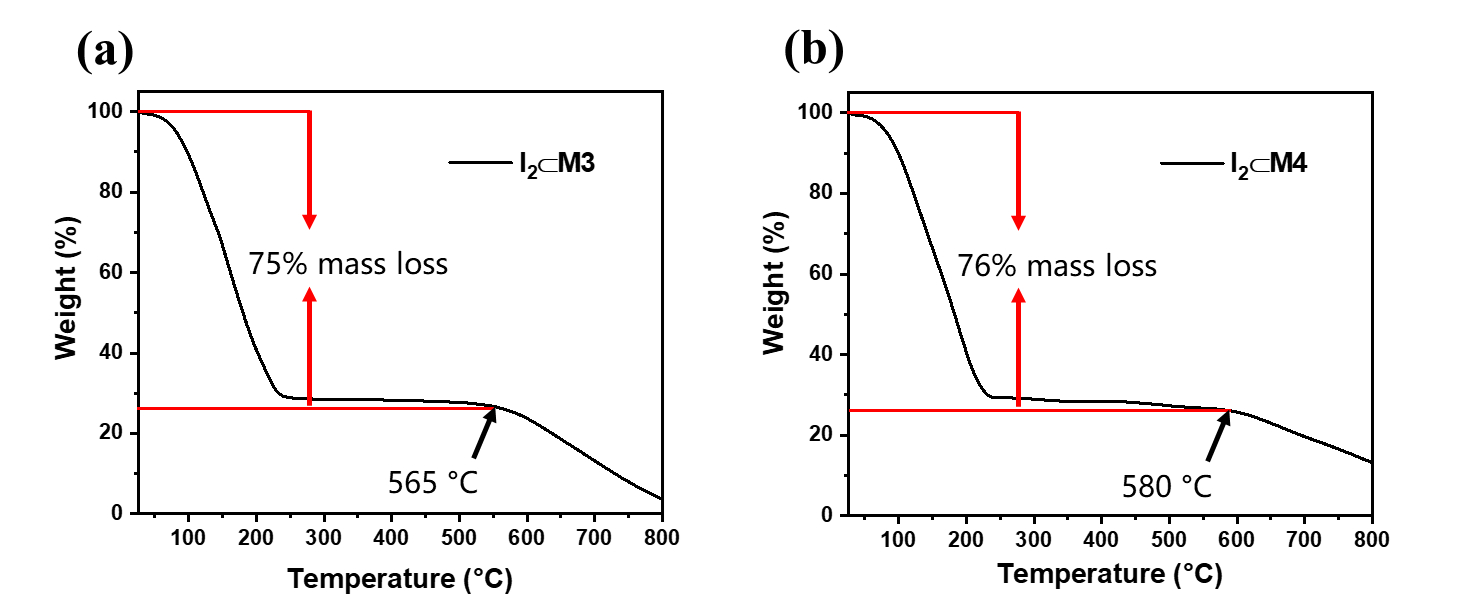


**Figure S10.** Thermogravimetric analysis of (a) I_2_⊂**M3** and (b) I_2_⊂**M4**.

**
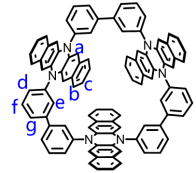
**

**
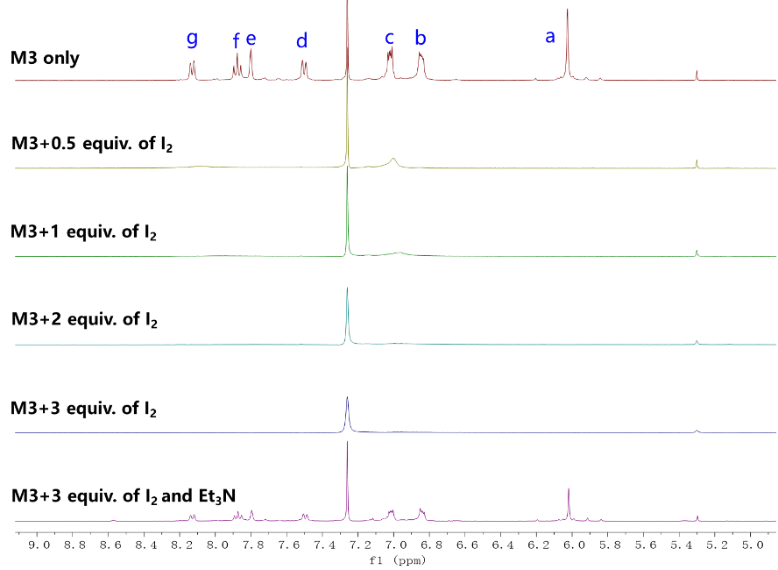
**

**Figure S11.** ^1^H NMR spectroscopic titrations (400 MHz, CDCl_3_, 298 K) of **M3** (3.45×10^-3^ _mM_, 0.5 µL) by incremental addition of aliquots (using a microsampler) of an I_2_ solution (0.13 _mM_) to the macrocycles’ solution and the iodine release by adding triethylamine.


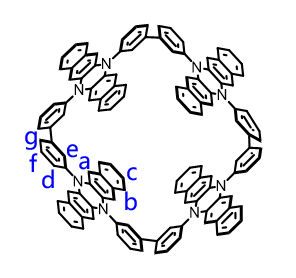


**
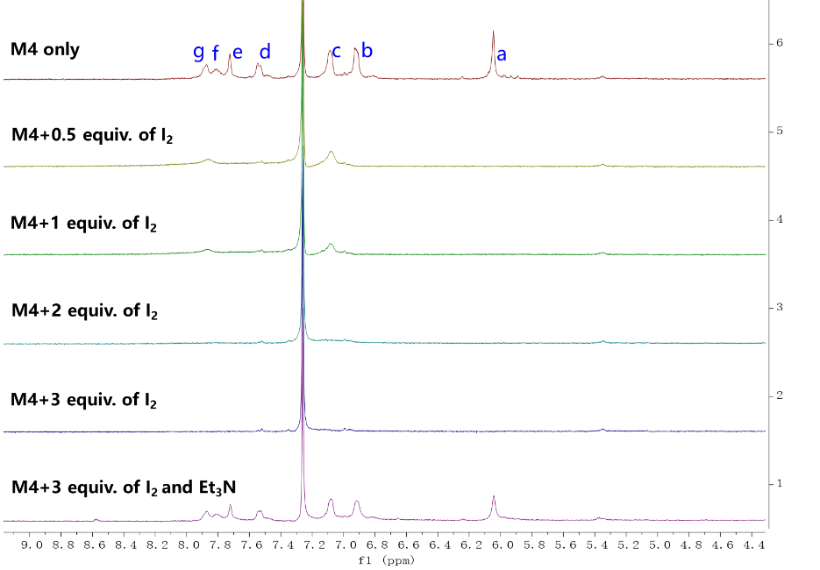
**

**Figure S12.** ^1^H NMR spectroscopic titrations (400 MHz, CDCl_3_, 298 K) of **M4** (3.45×10^-3^ _mM_, 0.5 µL) by incremental addition of aliquots (using a microsampler) of an I_2_ solution (0.13 _mM_) to the macrocycles’ solution and the iodine release by adding triethylamine.


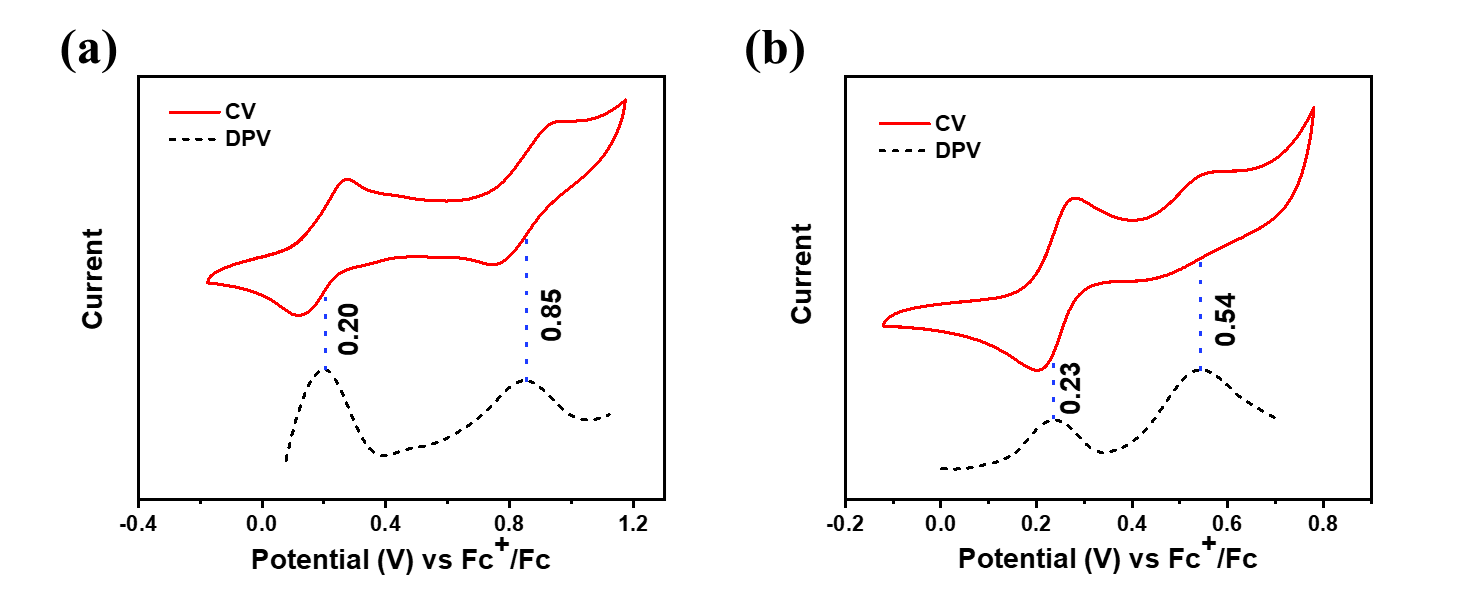


**Figure S13.** Cyclic voltammogram and differential pulse voltammetry of (a) **M3** and (b) **M4** in DCM with 0.1 _M_ *n*-Bu_4_NPF_6_ as the supporting electrolyte, Ag/AgCl as the reference electrode, and a Pt wire as the counter electrode and a scan rate at 20 mV s^-1^.


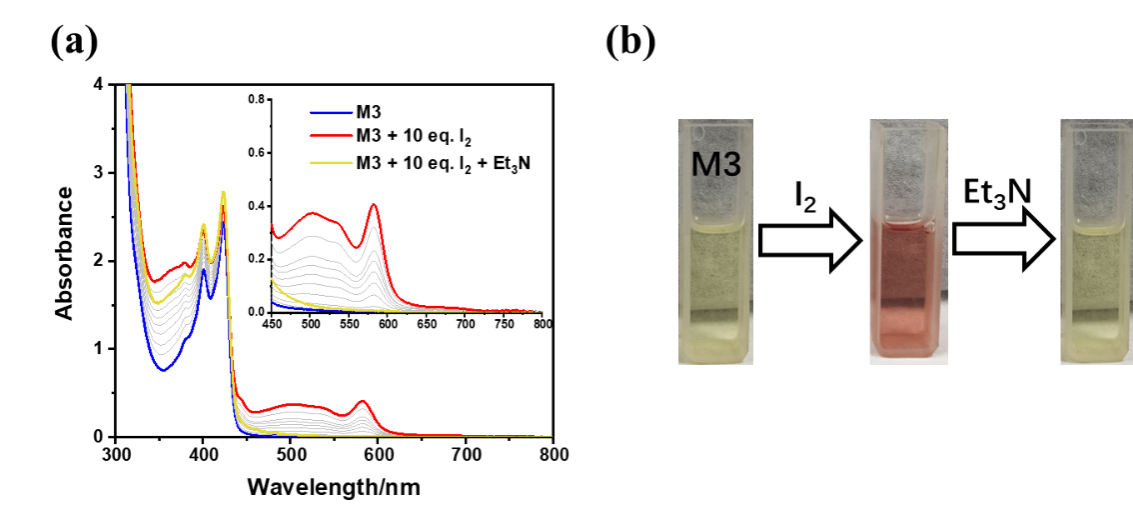


**Figure S14.** (a) UV–vis absorption spectra of **M3** (5×10^-5^ _M_ in dichloromethane) before and after adding different equivalents of iodine. (b) Photographs from left to right depict **M3**, **M3** after treatment with iodine, and following the addition of triethylamine in dichloromethane solution.


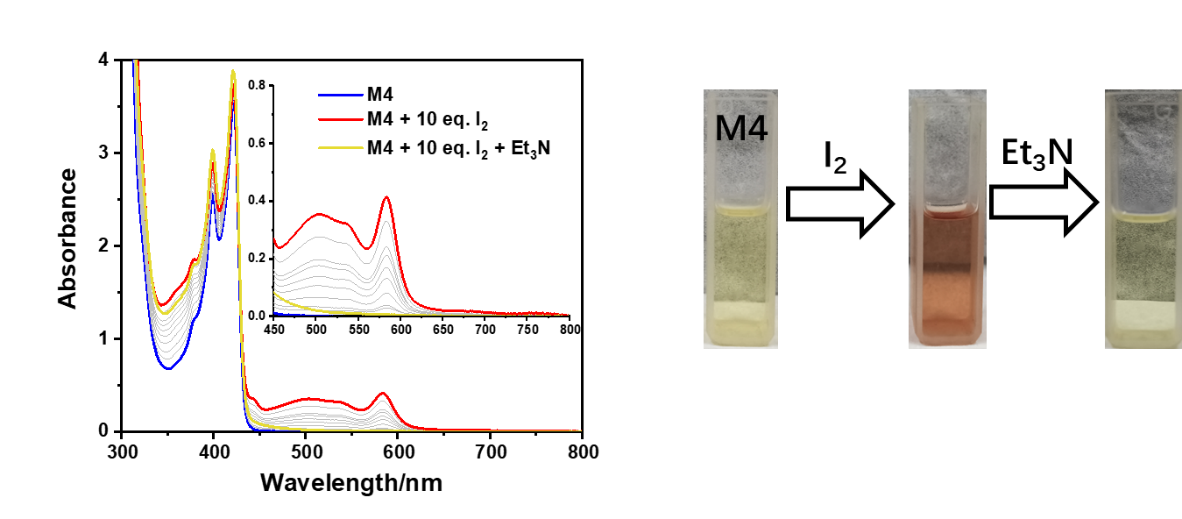


**Figure S15.** (a) UV–vis absorption spectra of **M4** (5×10^-5^ _M_ in dichloromethane) before and after adding different equivalents of iodine. (b) Photographs from left to right depict **M4**, **M4** after treatment with iodine, and following the addition of triethylamine in dichloromethane solution.


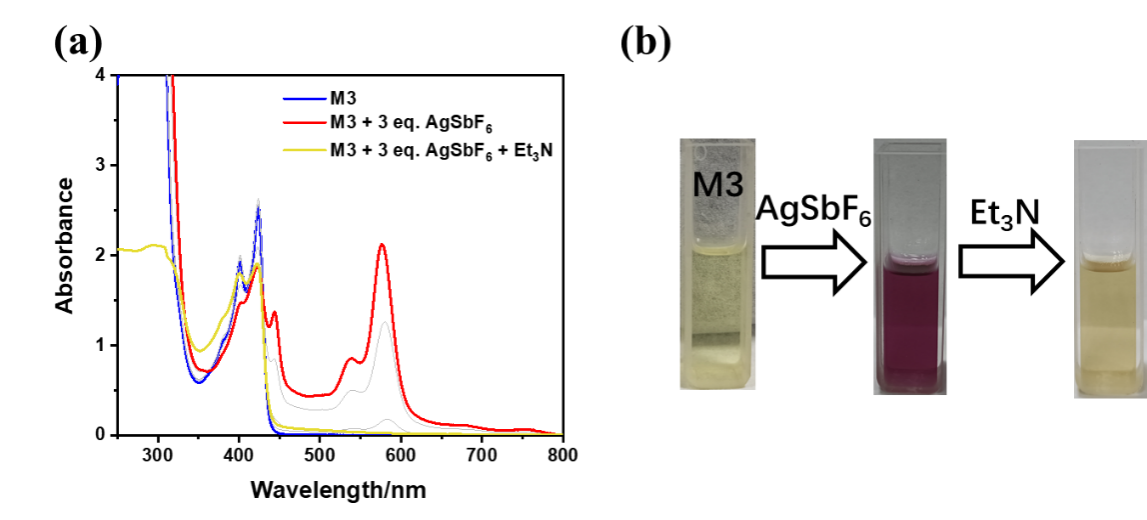


**Figure S16.** (a) UV–vis absorption spectra of **M3** (5×10^-5^ _M_ in dichloromethane) before and after adding different equivalents of AgSbF_6_. (b) Photographs from left to right depict **M3**, **M3** after treatment with AgSbF_6_, and following the addition of triethylamine in dichloromethane solution.


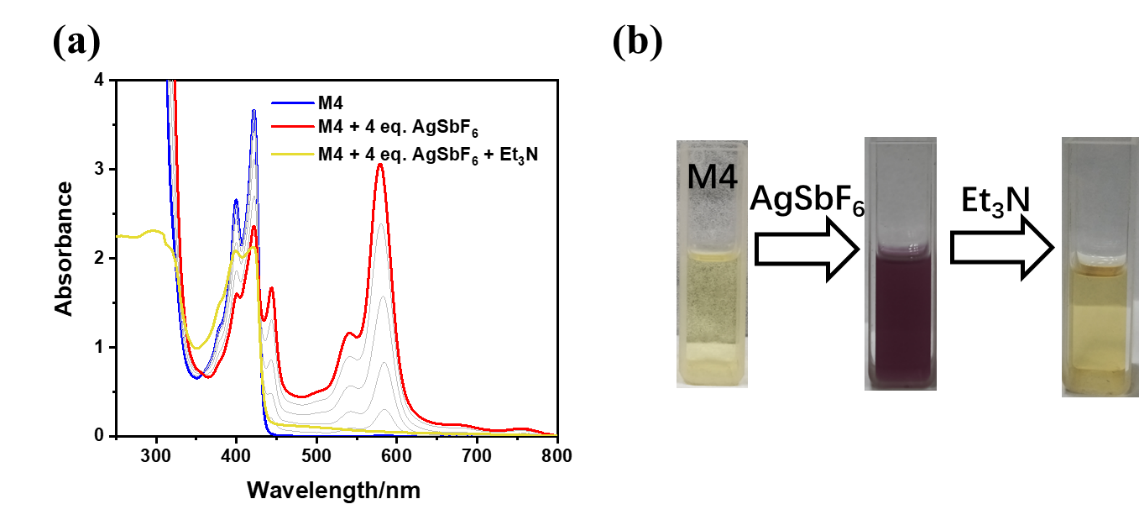


**Figure S17.** (a) UV–vis absorption spectra of **M4** (5×10^-5^ _M_ in dichloromethane) before and after adding different equivalents of AgSbF_6_. (b) Photographs from left to right depict **M4**, **M4** after treatment with AgSbF_6_, and following the addition of triethylamine in dichloromethane solution.


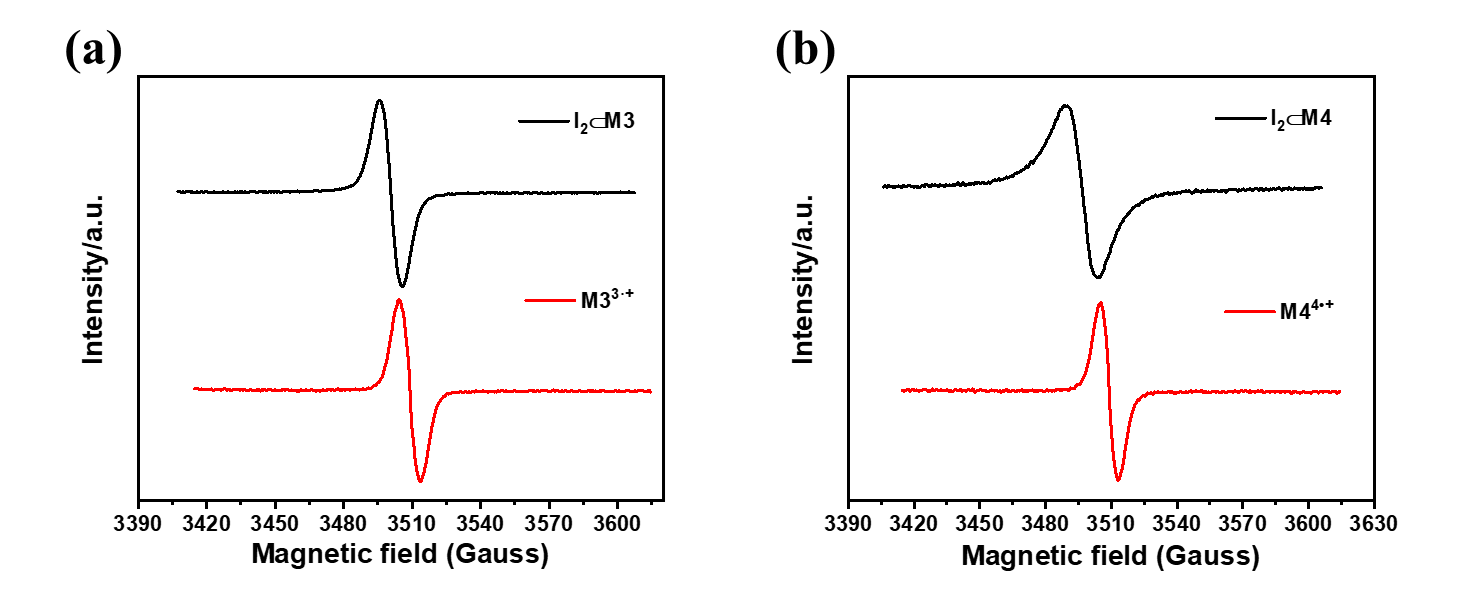


**Figure S18.** (a) EPR spectra of I_2_⊂**M3** and **M3** with 3 equiv. AgSbF_6_ in dichloromethane solution measured at room temperature. (b) EPR spectra of I_2_⊂**M4** and **M4** with 4 equiv. AgSbF_6_ in dichloromethane solution measured at room temperature.

**
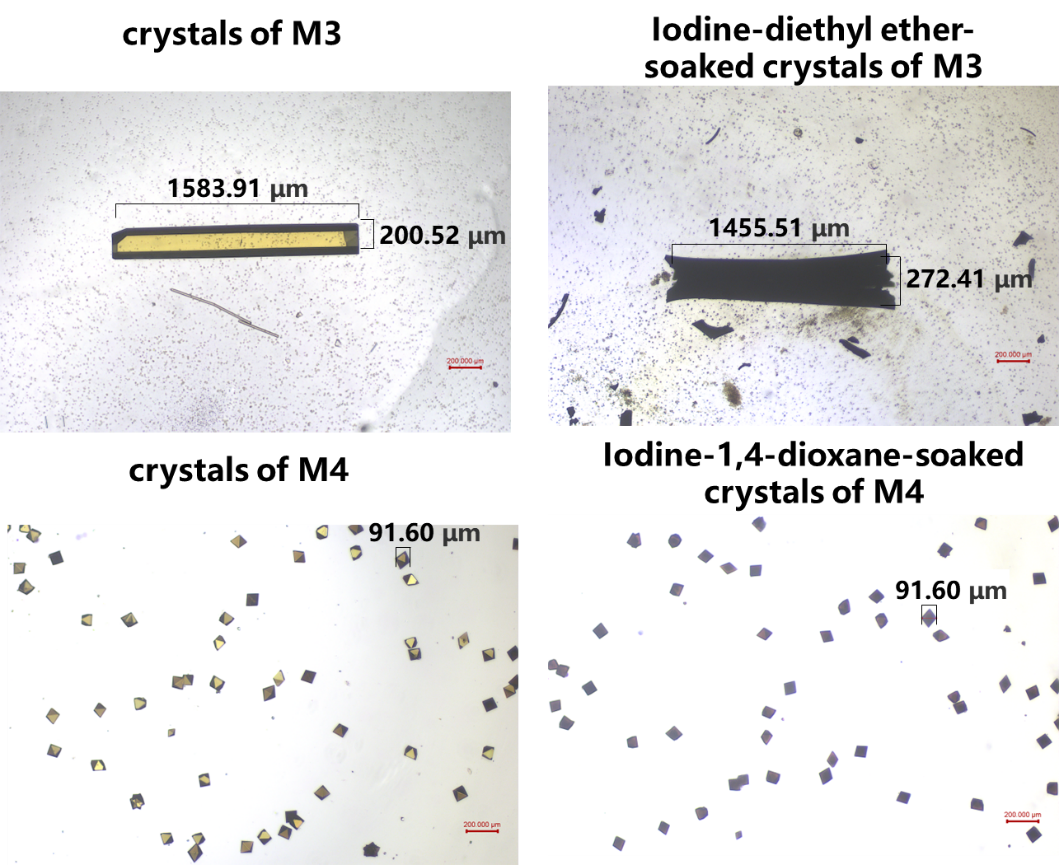
**

**Figure S19.** Schematic illustrations of **M3** and **M4** crystals before and after iodine solution soaking.

## 3. Device results


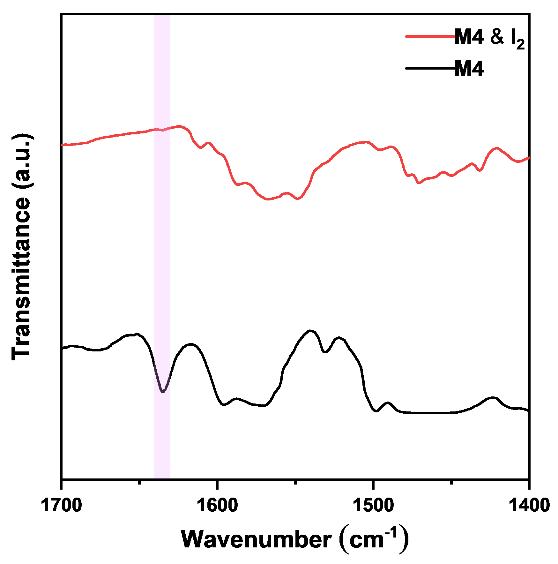


**Figure S20.** FT-IR spectrum of the **M4** and **M4** & I_2_, where **M4** powder and perovskite are put in sealed bottles and aged under light-heat conditions. Then the powder is taken out to obtain the **M4**&I_2_.


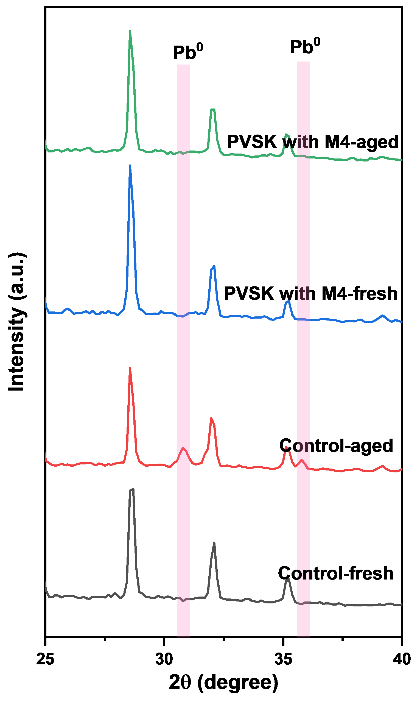


**Figure S21.** XRD of control perovskite and perovskite with **M4** before and after aging under light-heat aging.


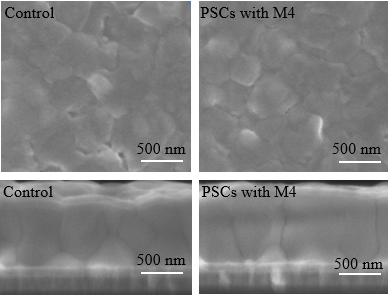


**Figure S22.** SEM of control perovskite and perovskite with **M4** before aging under light-heat aging.


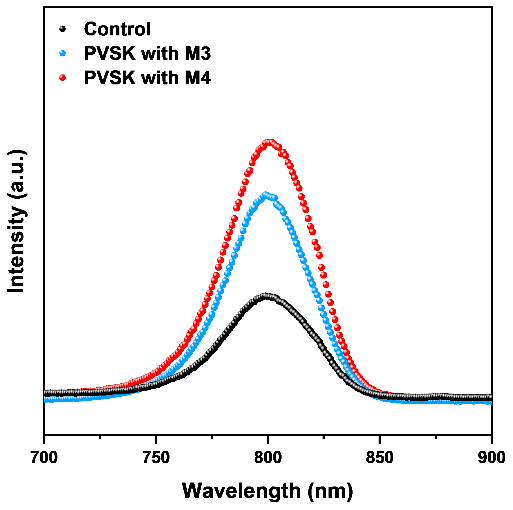


**Figure S23.** PL of control perovskite, perovskite with **M3** and perovskite with **M4.**


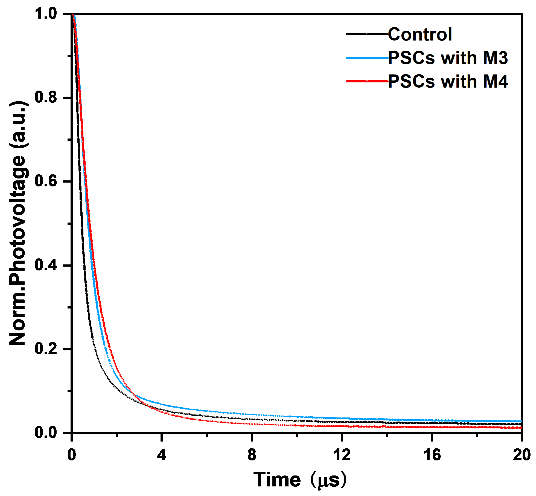


**Figure S24.** TPV of control PSCs, PSCs with **M3** and PSCs with **M4**.


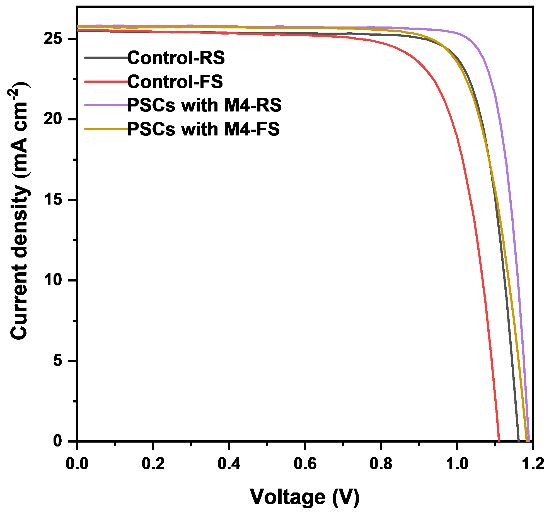


**Figure S25.** *J*–*V* curves of control and target PSCs measured by reverse and forward scans.


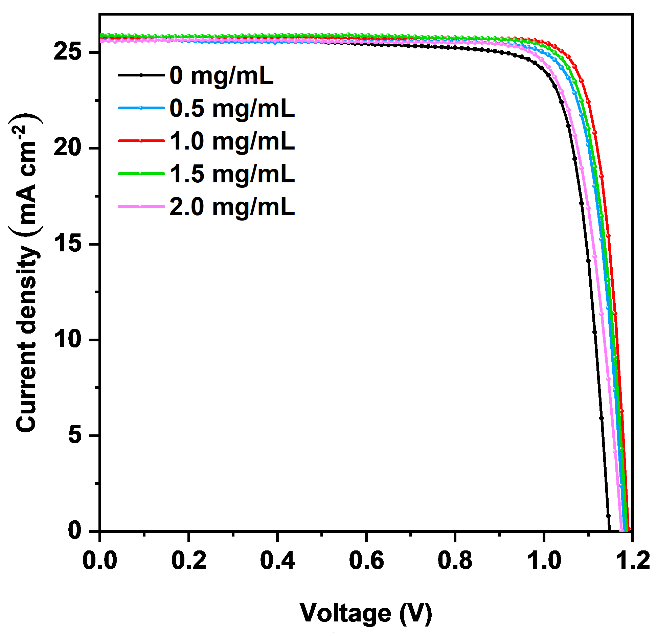


**Figure S26.** *J*–*V* curves of PSCs with different concentrations of **M4**.


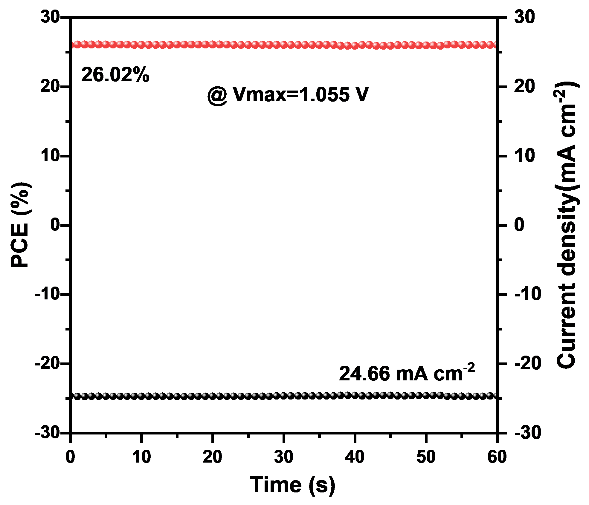


**Figure S27.** Stabilized maximum power point output of PSCs with **M4**.


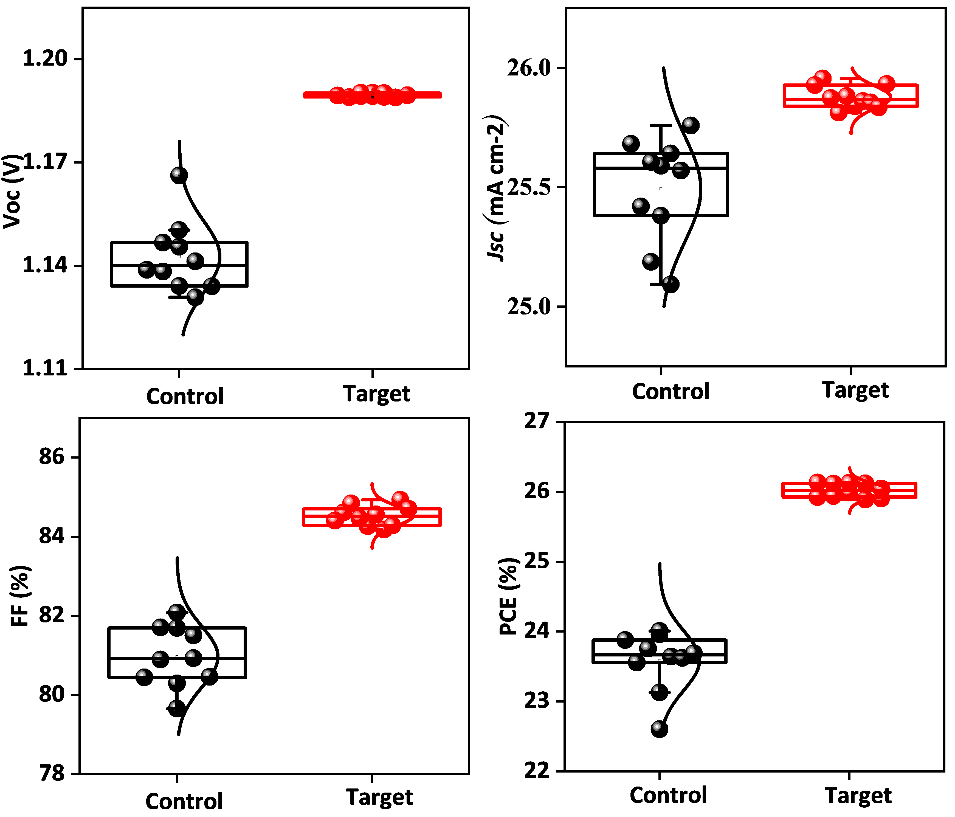


**Figure S28.** Boxplot of photovoltaic parameters based on 10 separated devices.


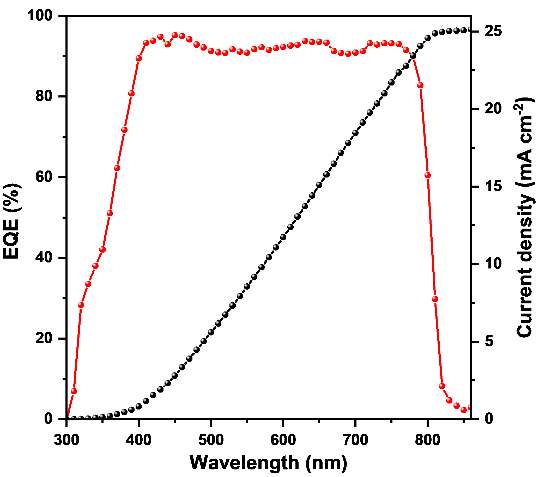


**Figure S29.** EQE spectrum of PSCs with **M4**.


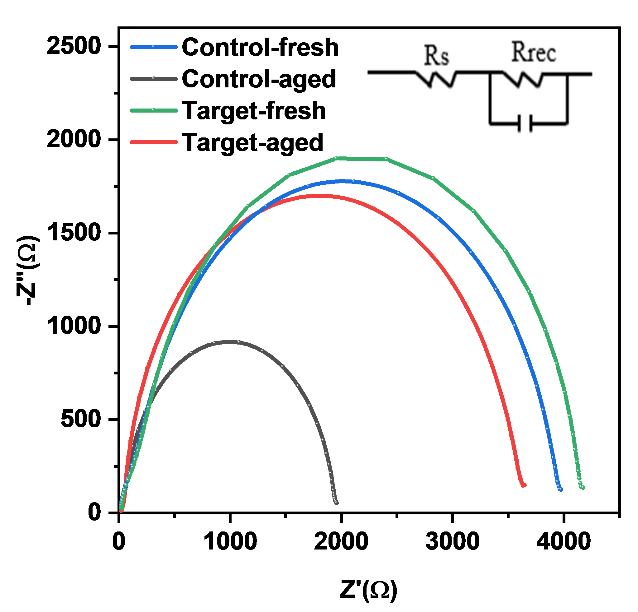


**Figure S30.** EIS of control and PSCs with **M4** before and after MPP tracking for 1000 h at 85 °C.

**Table S3.** TRPL parameters of devices with or without **M3/M4.**

| Device | τ_1_ (ns) | A_1_ | τ_2_ (ns) | A_2_ | τ_avg_ (ns) |
| --- | --- | --- | --- | --- | --- |
| Control | 340 | 0.57366 | 1042 | 0.17541 | 679 |
| PSCs with **M3** | 223 | 0.17161 | 1025 | 0.27607 | 929 |
| PSCs with **M4** | 408 | 0.13925 | 1149 | 0.22948 | 1017 |

## 4. X-ray crystallographic analysis

**
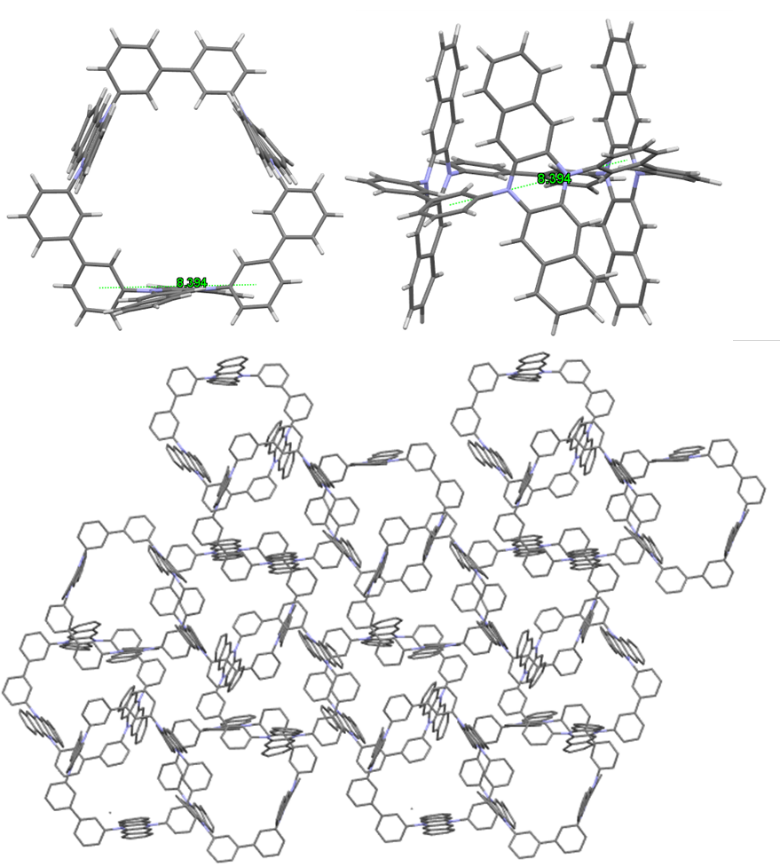
**

**Figure S31.** Single crystal structure and molecular packing of macrocycle **M3**. C, gray; H, white; N, blue. Hydrogen atoms and solvent are omitted for clarity.

**
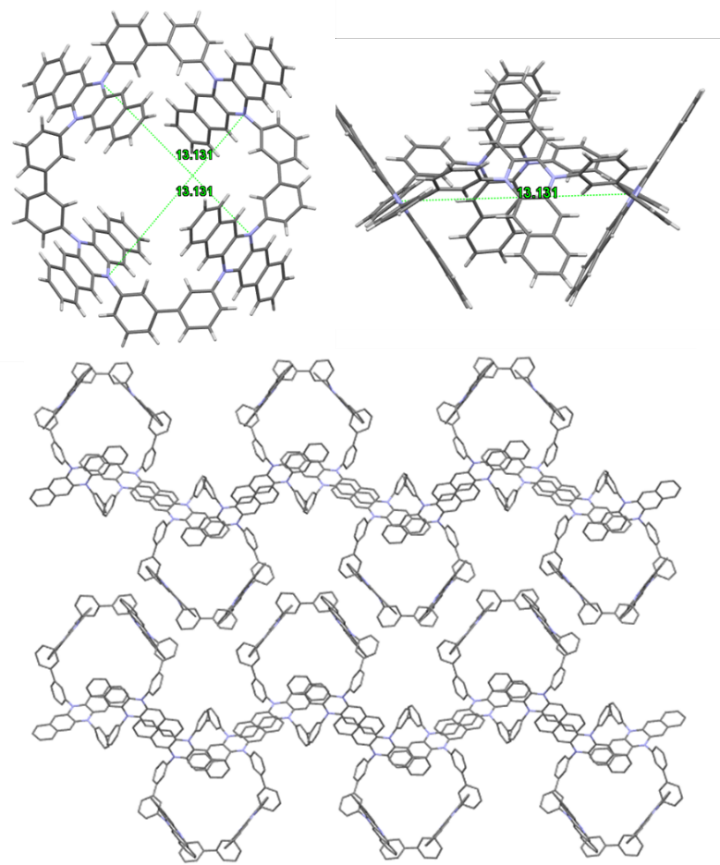
**

**Figure S32.** Single crystal structure and molecular packing of macrocycle **M4**. C, gray; H, white; N, blue. Hydrogen atoms and solvent are omitted for clarity.


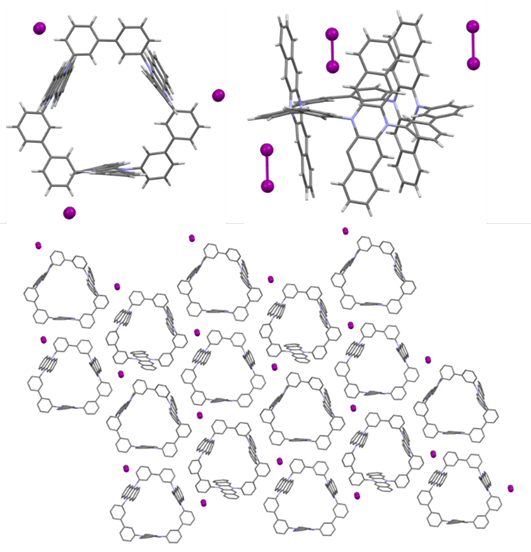


**Figure S33.** Single crystal structure and molecular packing of single crystal structures of I_2_⊂**M3**. C, gray; H, white; N, blue; I, purple. Hydrogen atoms and solvent are omitted for clarity.

**Table S4**. Crystal data and structure refinement for **M3** and **M4**.

| Compound | **M3** | **M4** |
| --- | --- | --- |
| Empirical formula | C_96_H_60_N_6_ | C_128_H_80_N_8_ |
| Formula weight | 1297.49 | 1729.65 |
| Temperature/K | 173.00(10) | 173.00(10) |
| Crystal system | triclinic | tetragonal |
| Space group | P-1 | I41/acd |
| a/Å | 19.3678(7) | 22.3314(3) |
| b/Å | 27.5928(13) | 22.3314(3) |
| c/Å | 27.6484(12) | 51.7257(12) |
| α/° | 60.330(5) | 90 |
| β/° | 89.675(3) | 90 |
| γ/° | 89.110(3) | 90 |
| Volume/Å^3^ | 12836.8(11) | 25795.2(9) |
| Z | 2 | 32 |
| ρcalcg/cm^3^ | 1.007 | 1.195 |
| μ/mm^‑1^ | 0.454 | 1.689 |
| F(000) | 4068.0 | 9696.0 |
| Crystal size/mm^3^ | 0.32 × 0.26 × 0.22 | 0.24 × 0.19 × 0.12 |
| Radiation | Cu Kα (λ = 1.54184) | Cu Kα (λ = 1.54184) |
| 2Θ range for data collection/° | 5.824 to 134.16 | 6.558 to 134.144 |
| Index ranges | -18 ≤ h ≤ 23,  -32 ≤ k ≤ 32,  -33 ≤ l ≤ 33 | -26 ≤ h ≤ 26,  -26 ≤ k ≤ 26,  -61 ≤ l ≤ 57 |
| Reflections collected | 158836 | 135251 |
| Independent reflections | 45595 [R_int_ = 0.1438,  R_sigma_ = 0.1575] | 5754 [R_int_ = 0.0978,  R_sigma_ = 0.0319] |
| Data/restraints/parameters | 45595/222/2786 | 5754/315/452 |
| Goodness-of-fit on F^2^ | 0.985 | 1.222 |
| Final R indexes [I>=2σ (I)] | R_1_ = 0.1534, wR_2_ = 0.3691 | R_1_ = 0.1423, wR_2_ = 0.3000 |
| Final R indexes [all data] | R_1_ = 0.2142, wR_2_ = 0.4027 | R_1_ = 0.1648, wR_2_ = 0.3114 |
| Largest diff. peak/hole / e Å^-3^ | 0.53/-0.39 | 0.79/-0.52 |
| CCDC | 2463878 | 2463874 |

**Table S5**. Crystal data and structure refinement for I_2_⊂**M3**.

| Compound | I_2_⊂**M3** |
| --- | --- |
| Empirical formula | C_96_H_60_N_6_I_2_ |
| Formula weight | 1551.38 |
| Temperature/K | 293(2) |
| Crystal system | trigonal |
| Space group | P-3 |
| a/Å | 27.530 |
| b/Å | 27.530 |
| c/Å | 19.305 |
| α/° | 90 |
| β/° | 90 |
| γ/° | 120 |
| Volume/Å^3^ | 12670.8 |
| Z | 1 |
| ρcalcg/cm^3^ | 1.120 |
| μ/mm^‑1^ | 0.173 |
| F(000) | 4462.0 |
| Crystal size/mm^3^ | 0.16 × 0.12 × 0.11 |
| Radiation | synchrotron radiation (λ = 0.68922) |
| 2Θ range for data collection/° | 2.87 to 48.62 |
| Index ranges | -32 ≤ h ≤ 32, -32 ≤ k ≤ 32, -23 ≤ l ≤ 23 |
| Reflections collected | 257528 |
| Independent reflections | 15010 [R_int_ = 0.0708, R_sigma_ = 0.0307] |
| Data/restraints/parameters | 15010/161/976 |
| Goodness-of-fit on F^2^ | 1.024 |
| Final R indexes [I>=2σ (I)] | R_1_ = 0.1778, wR_2_ = 0.4237 |
| Final R indexes [all data] | R_1_ = 0.1872, wR_2_ = 0.4293 |
| Largest diff. peak/hole / e Å^-3^ | 1.47/-1.11 |
| CCDC | 2465442 |

## 5. NMR and mass spectra

**
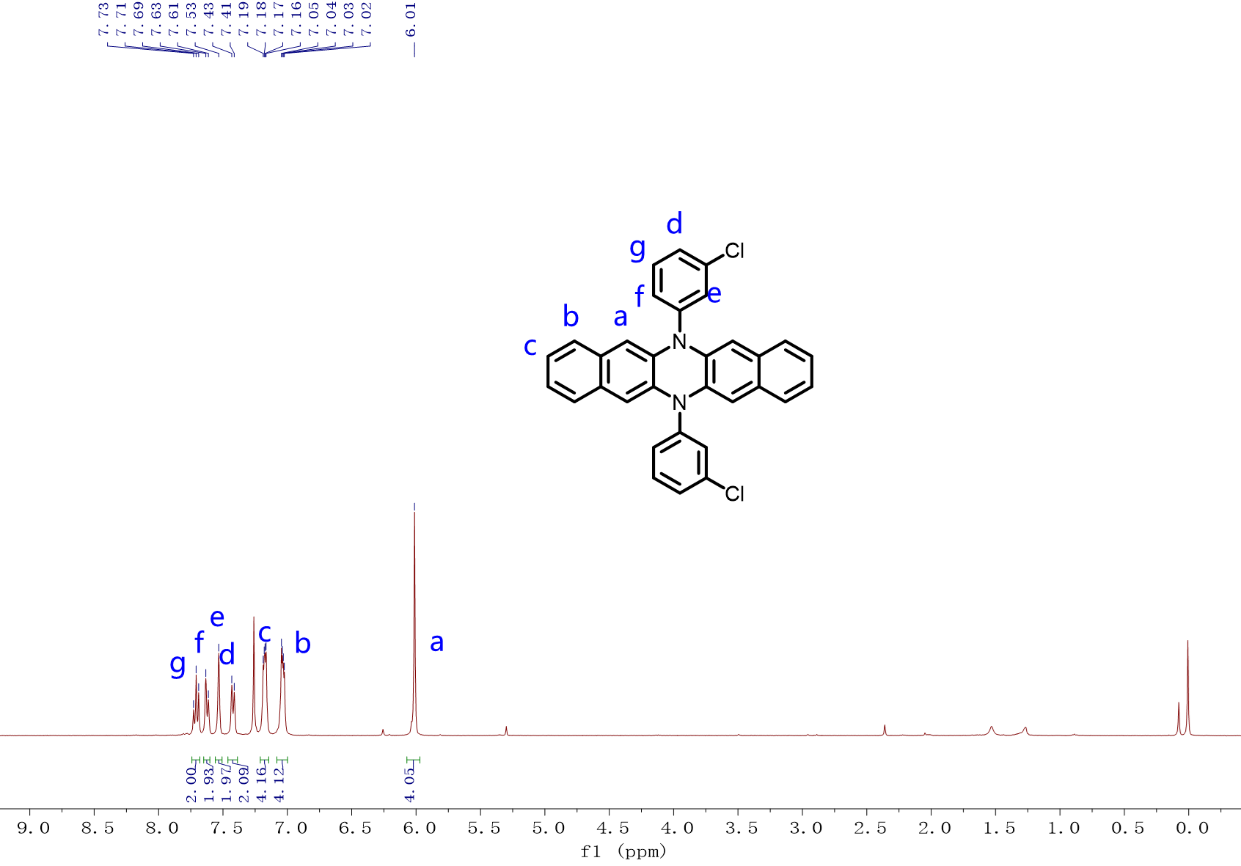
**

**Figure S34.** ^1^H NMR (400 MHz, CDCl_3_, 298 K) spectrum of compound **2**.

**
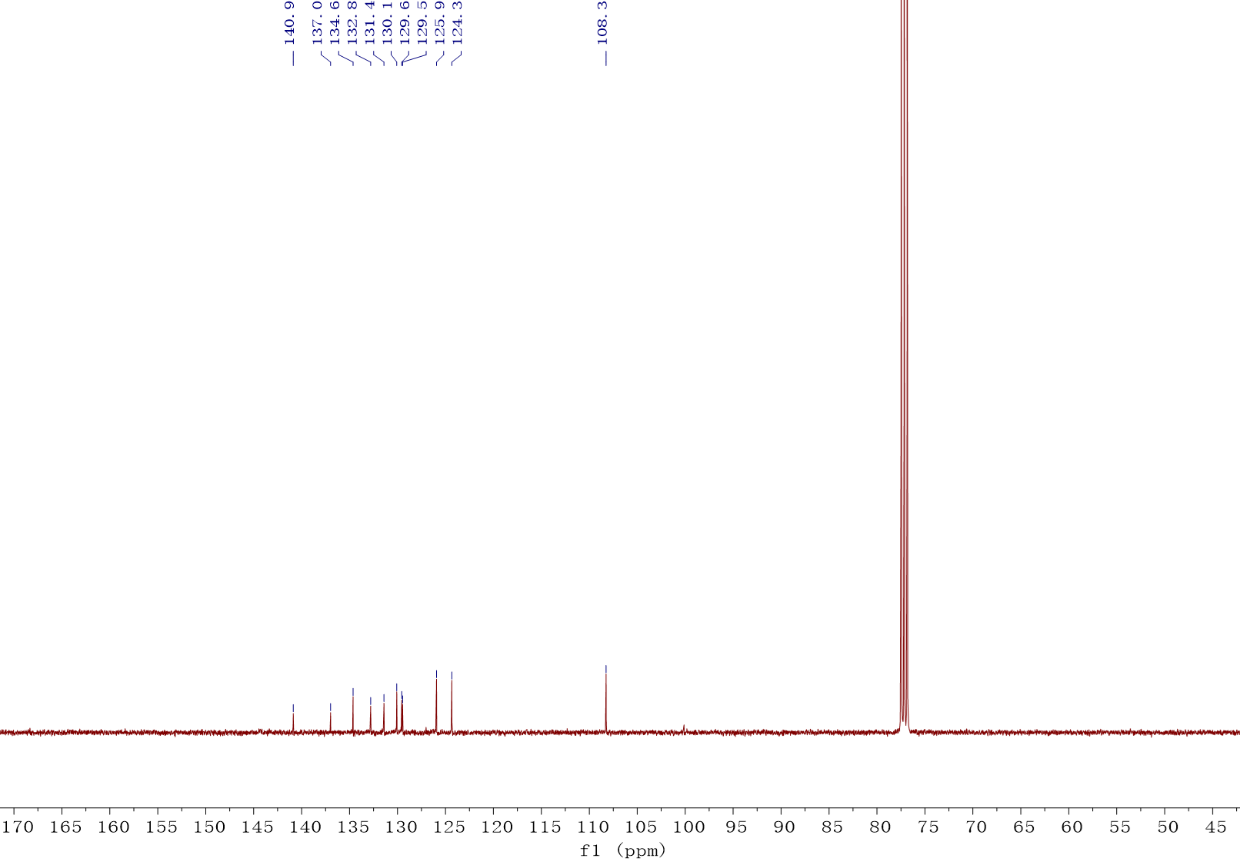
**

**Figure S35.** ^13^C NMR (101 MHz, CDCl_3_, 298 K) spectrum of compound **2**.


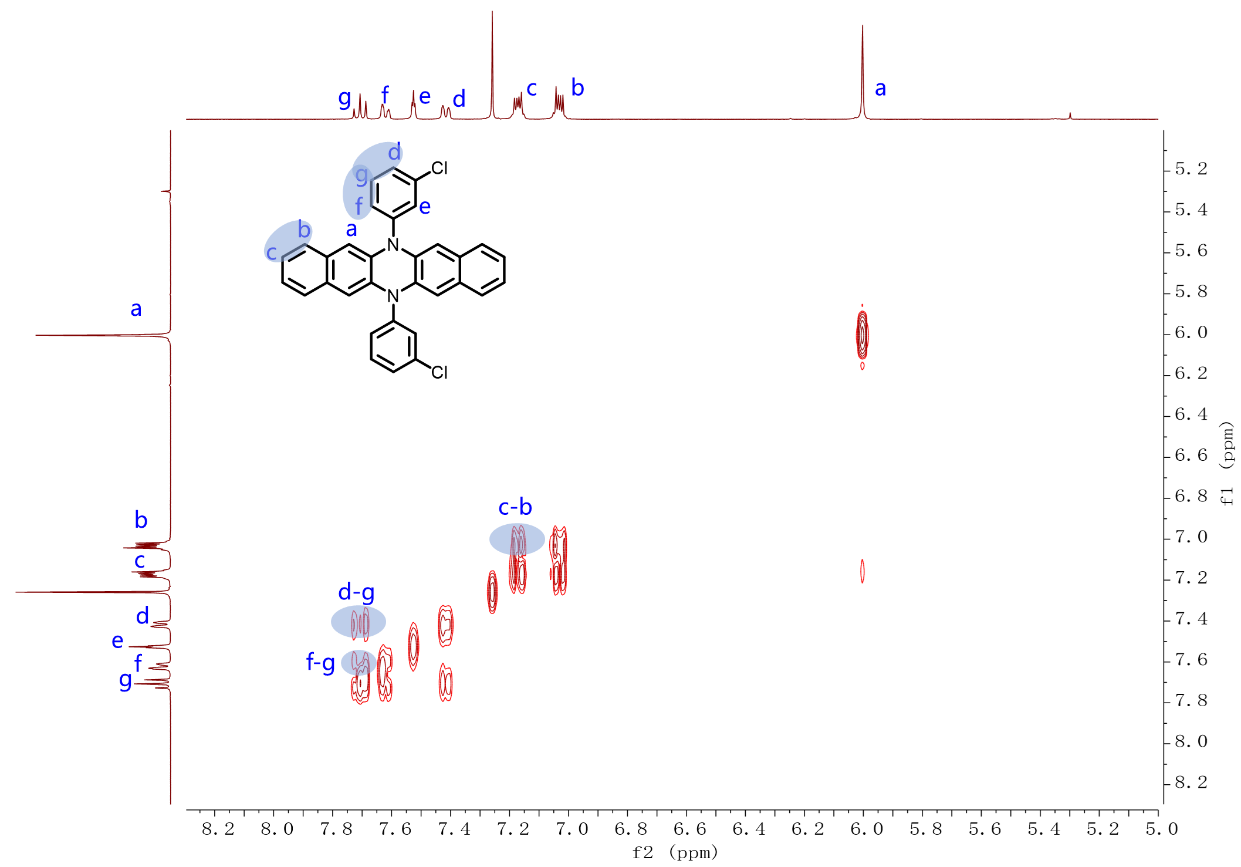


**Figure S36.** ^1^H-^1^H COSY NMR spectrum (400 MHz, 298 K, CDCl_3_) of compound **2**.

**
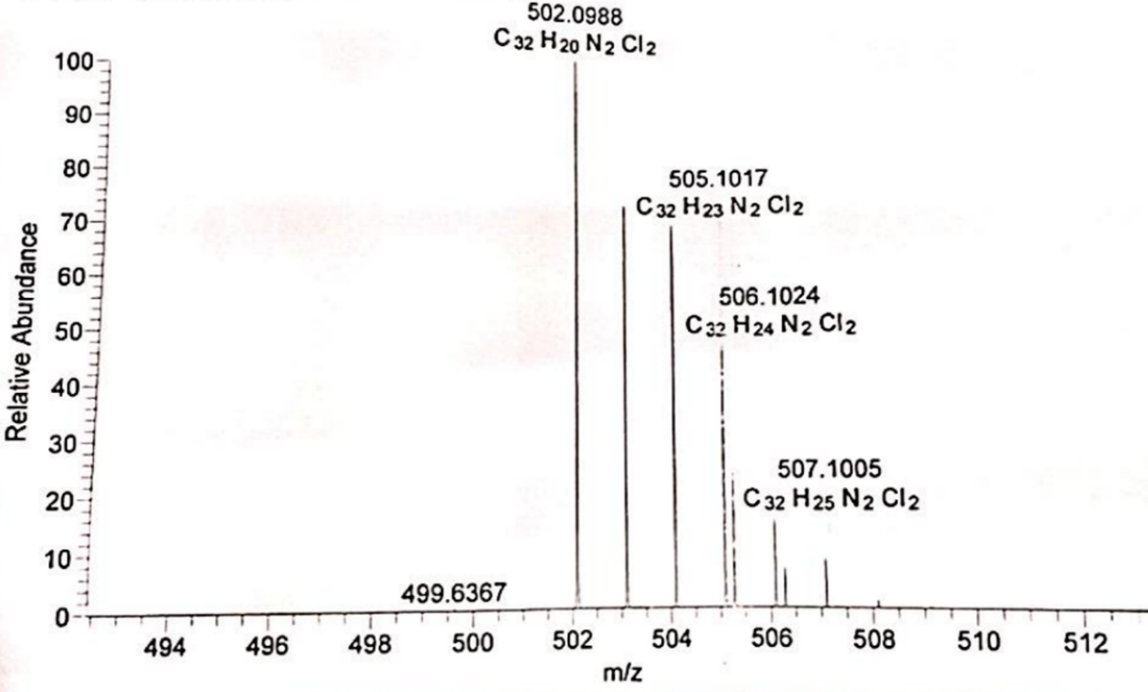
**

**Figure S37.** High-resolution ESI-MS spectrum of compound **2**.

**
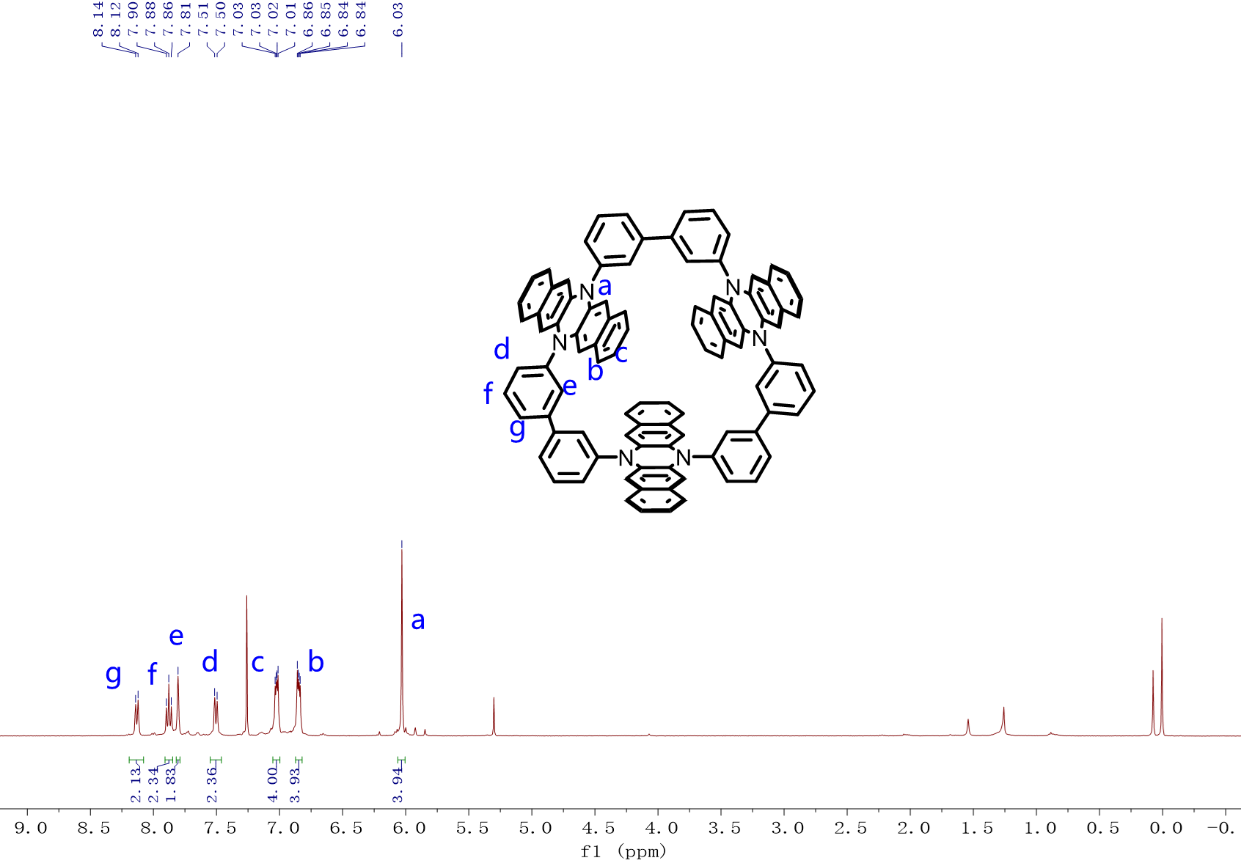
**

**Figure S38.** ^1^H NMR (400 MHz, CDCl_3_, 298 K) spectrum of macrocycle **M3**.


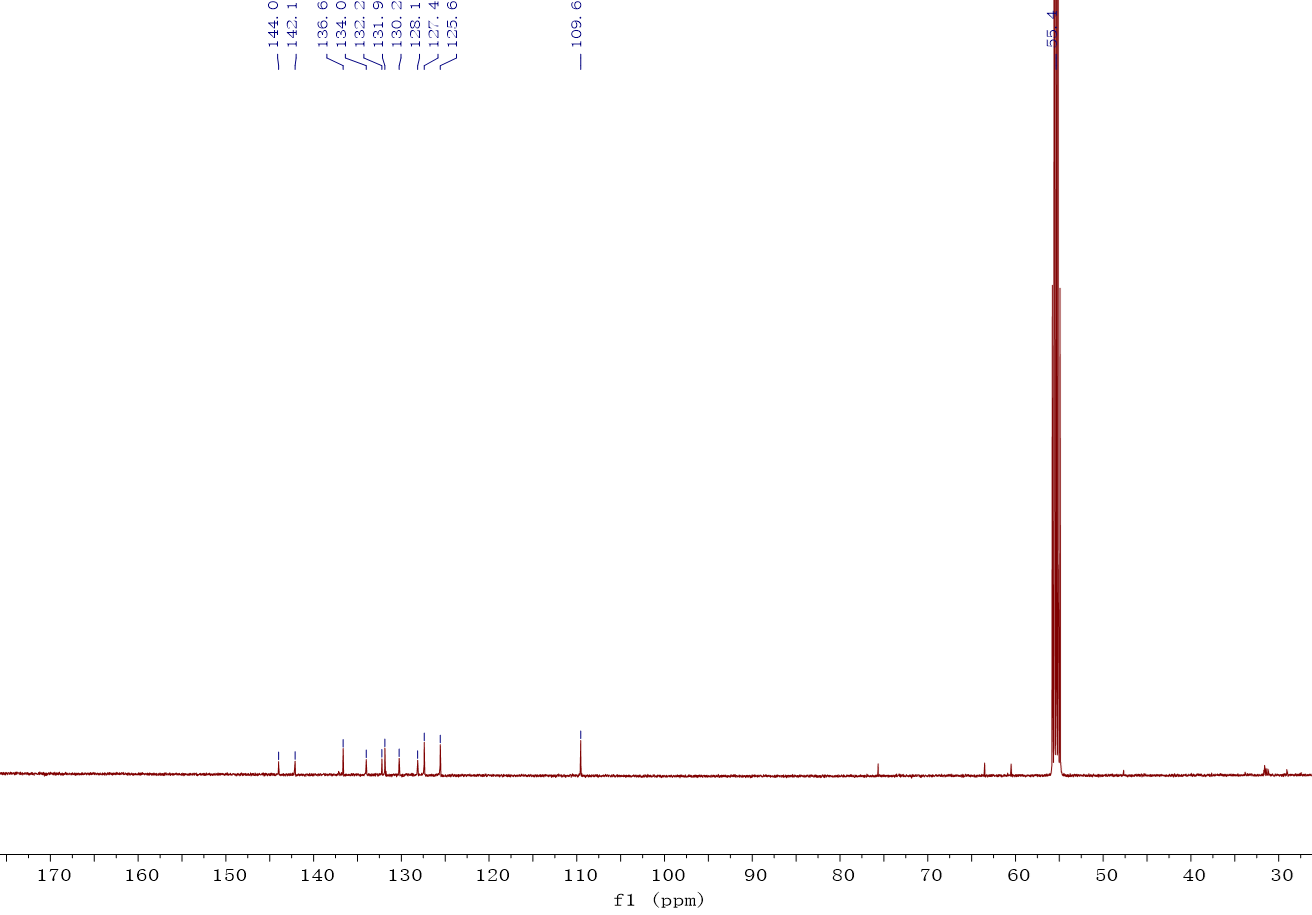


**Figure S39.** ^13^C NMR (125 MHz, CD_2_Cl_2_, 298 K) spectrum of **M3**. (Due to the poor solubility of **M4** in CDCl_3_ and CD_2_Cl_2_, we were unable to obtain its clear ^13^C NMR spectrum.)


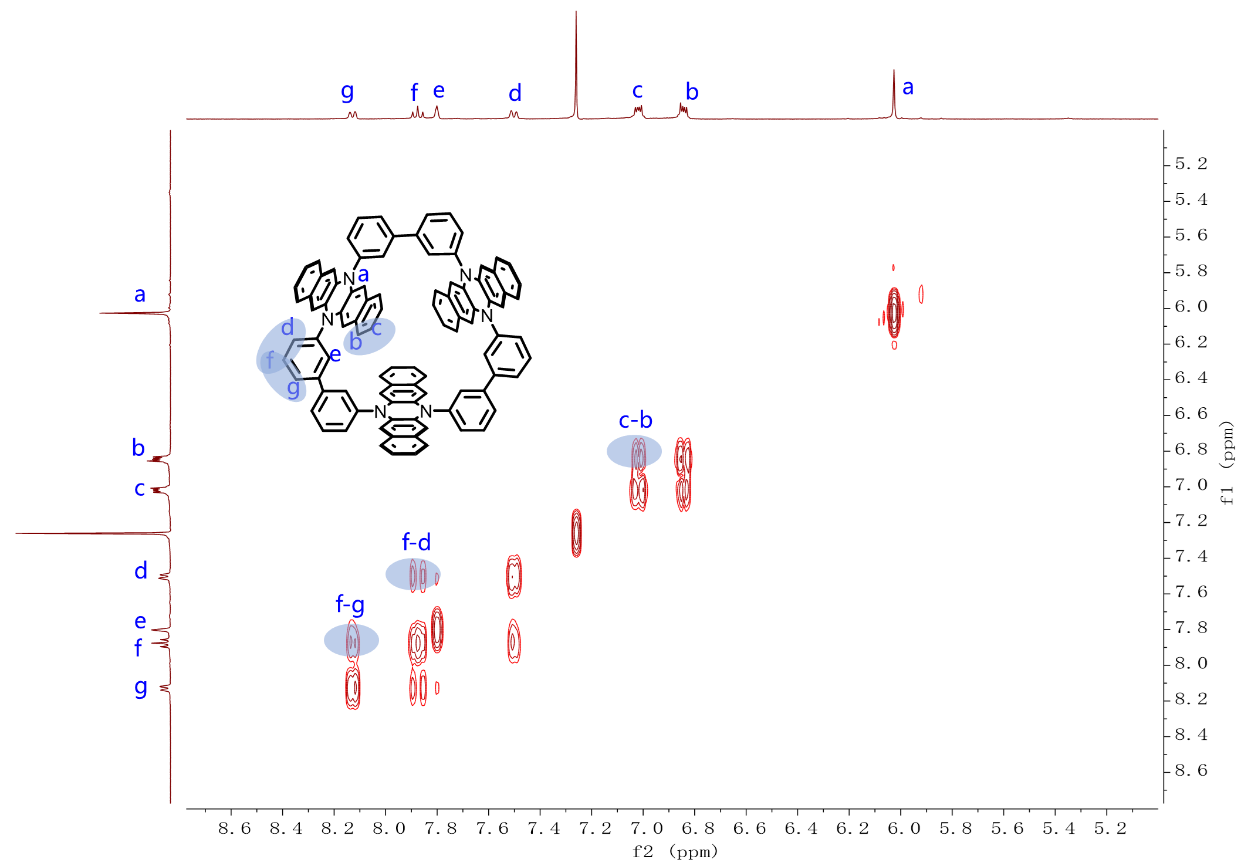


**Figure S40.** ^1^H-^1^H COSY NMR spectrum (400 MHz, 298 K, CDCl_3_) of macrocycle **M3**.

**
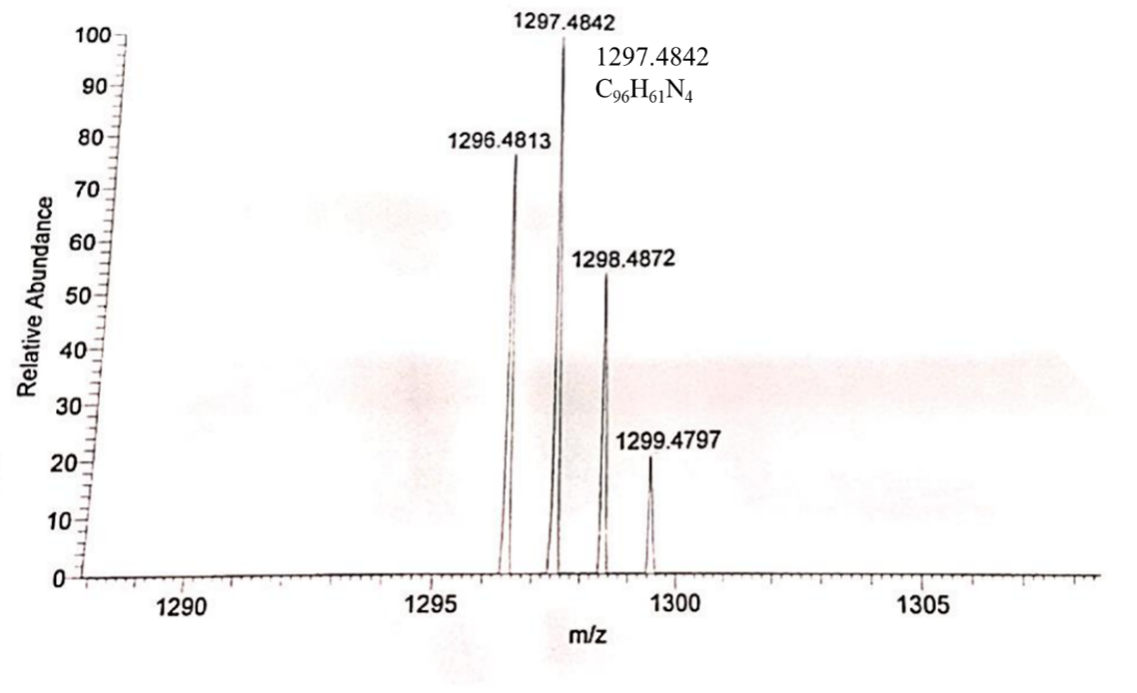
**

**Figure S41.** High-resolution ESI-MS spectrum of macrocycle **M3.**


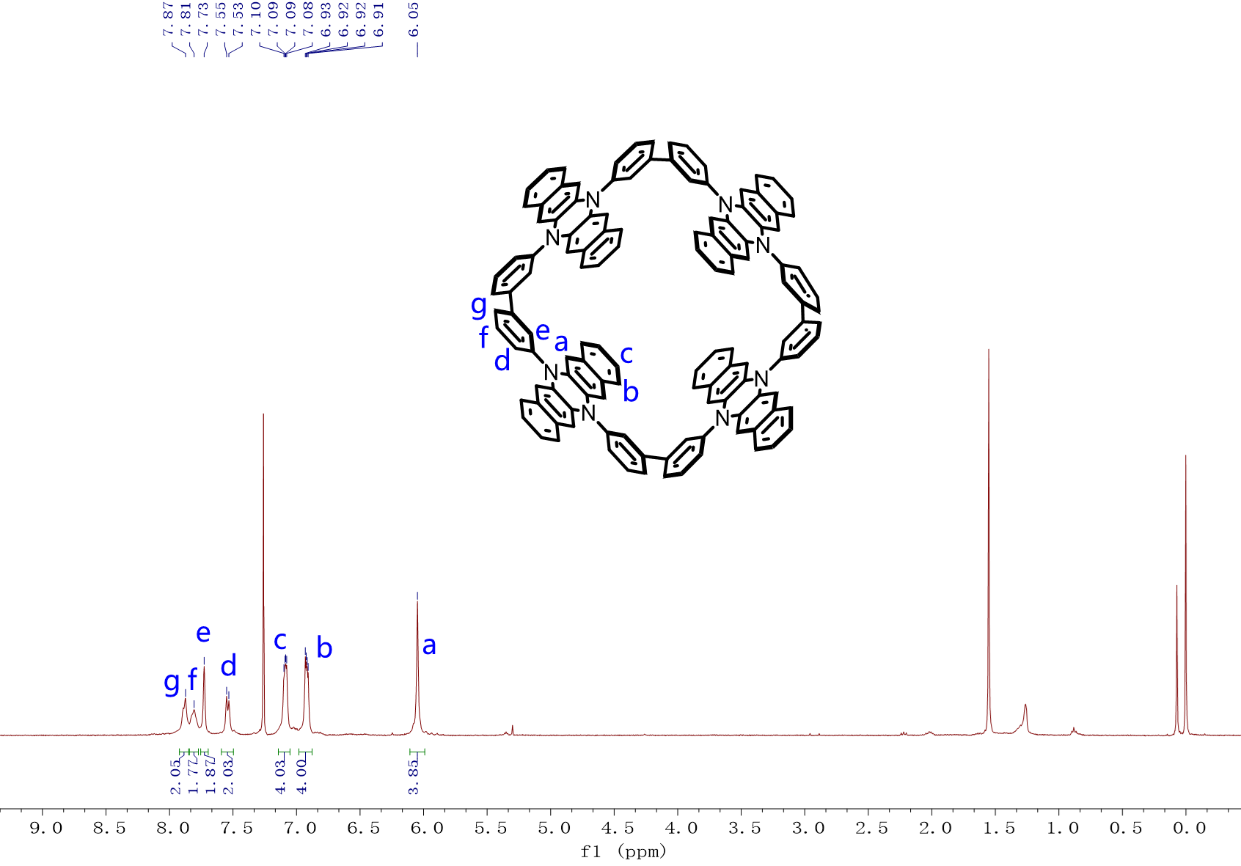


**Figure S42.** ^1^H NMR (400 MHz, CDCl_3_, 298 K) spectrum of macrocycle **M4**.


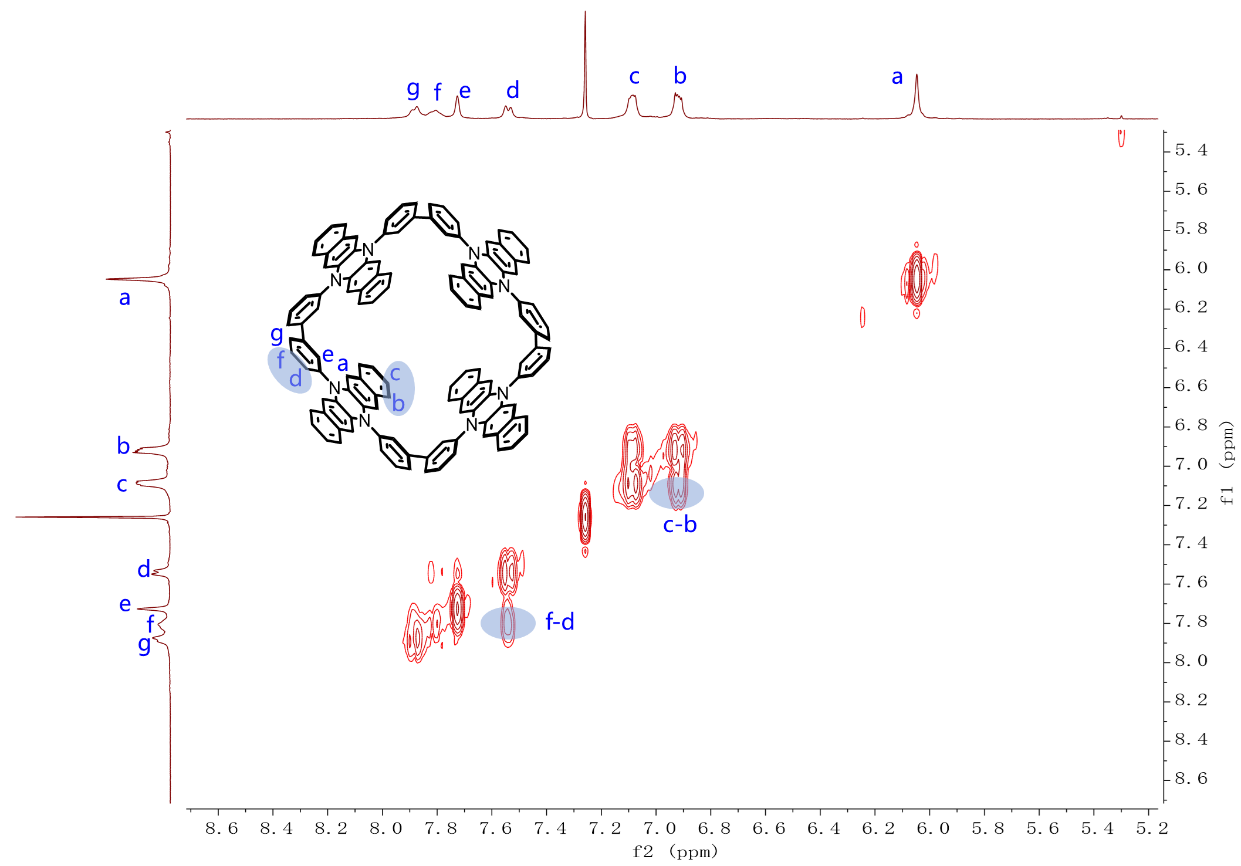


**Figure S43.** ^1^H-^1^H COSY NMR spectrum (400 MHz, 298 K, CDCl_3_) of macrocycle **M4**.

**
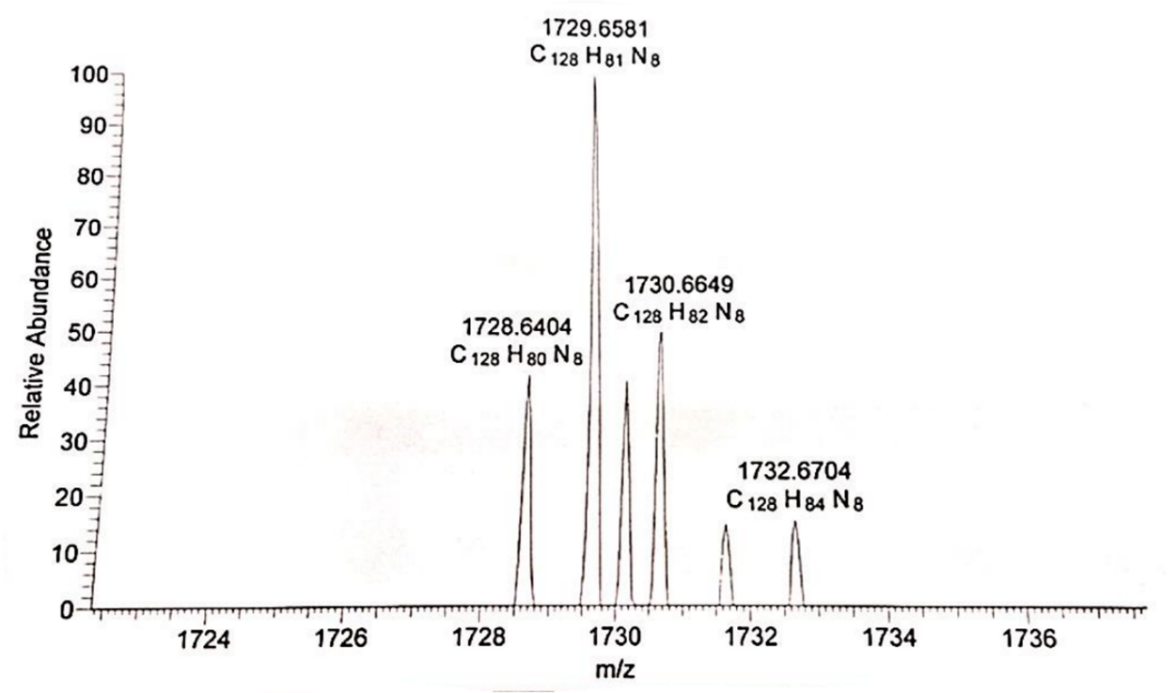
**

**Figure S44.** High-resolution ESI-MS spectrum of macrocycle **M4**.


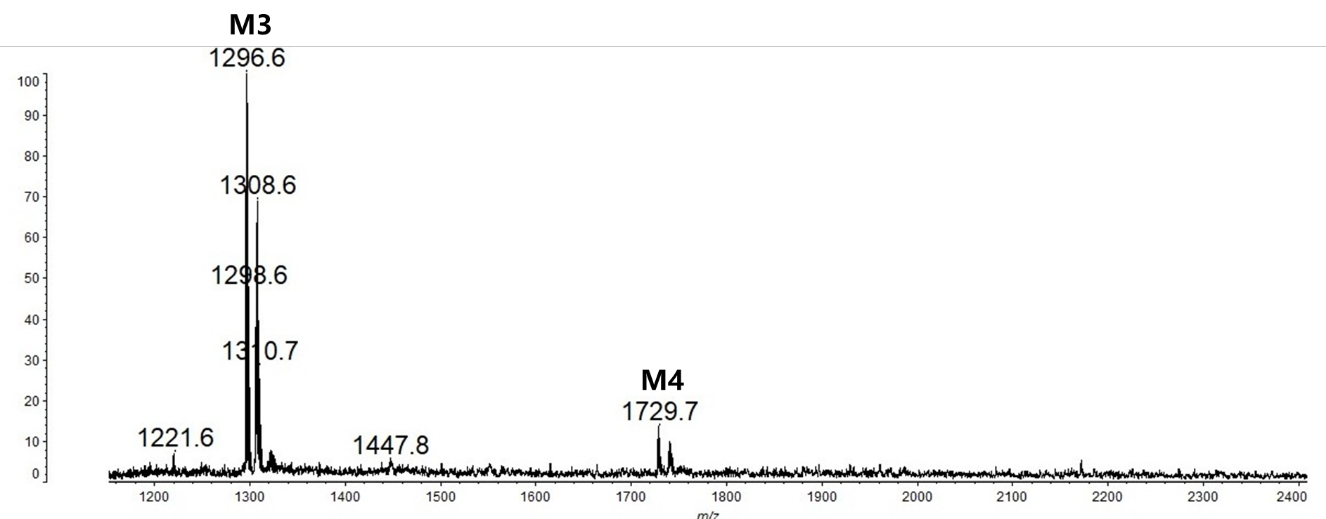


**Figure S45**. MALDI-TOF MS spectrum of the crude product.

# 6. Reference

1. B. Li, B. Wang, X. Huang, L. Dai, L. Cui, J. Li, X. Jia, C. Li, *Angew. Chem. Int. Ed.* **2019**, *58*, 3885.
2. J. Yang, S.-J. Hu, L.-X. Cai, L.-P. Zhou, Q.-F. Sun, *Nat. Commun.* **2023**, *14*, 6082.
