# Supplementary material for: Supramolecular Macrocyclic Iodine Adsorbents Enable Photothermally Stable Perovskite Solar Cells
Source: Adv Sci (Weinh). 2025 Oct 30;13(4):e16964. doi: 10.1002/advs.202516964 (PMC12822466; doi:10.1002/advs.202516964)
Supplement: Supplementary file 2 — Supplemental DataFile [file ADVS-13-e16964-s001.zip › M3-I2_checkCIF.pdf]

checkCIF (basic structural check) running

checkCIF/PLATON (basic structural check)

You have not supplied any structure factors. As a result the full set of tests cannot be run.

No syntax errors found. CIF dictionary
Please wait while processing .... Interpreting this report

Structure factor report

Datablock: wy222_0m

Bond precision:	C-C = 0.0138 A		Wavelength=0.68922
Cell:	a=27.53000	b=27.53000	c=19.30500
	alpha=90	beta=90	gamma=120
Temperature: 293 K			

	Calculated	Reported
Volume	12671.059	12671
Space group	P -3	P -3
Hall group	-P 3	-P 3
Moiety formula	6(C96 H60 N6), 6(C6 H12), I2 [+ solvent]	6(C96 H60 N6), 6(C6 H12), I2
Sum formula	C612 H432 I2 N36 [+ solvent]	C612 H432 I2 N36
Mr	8543.76	8543.71
Dx,g cm-3	1.120	1.120
Z	1	1
Mu (mm-1)	0.170	0.173
F000	4462.0	4462.0
F000'	4462.27	
h,k,lmax	32,32,23	32,32,23
Nref	15099	15010
Tmin,Tmax	0.975,0.981	0.464,0.745
Tmin'	0.973	

Correction method= # Reported T Limits: Tmin=0.464 Tmax=0.745 AbsCorr = MULTI-SCAN

Data completeness= 0.994 Theta(max)= 24.310

R(reflections)= 0.1778(12946) wR2(reflections)= 0.4293(15010)

S = 1.024 Npar= 976

The following ALERTS were generated. Each ALERT has the format
test-name_ALERT_alert-type_alert-level.
Click on the hyperlinks for more details of the test.

Alert level B

PLAT082_ALERT_2_B High R1 Value .... 0.18 Report
PLAT084_ALERT_3_B High wR2 Value (i.e. > 0.25) .... 0.43 Report
PLAT230_ALERT_2_B Hirshfeld Test Diff for C67 --C72 . 14.4 s.u.
PLAT260_ALERT_2_B Large Average Ueq of Residue Including I3 0.701 Check
PLAT411_ALERT_2_B Short Inter H...H Contact H10F ..H22 . 1.97 Ang.
x,y,z = 1_555 Check
PLAT411_ALERT_2_B Short Inter H...H Contact H10F ..H32 . 1.86 Ang.
x,y,z = 1_555 Check

Alert level C

PLAT141_ALERT_4_C s.u. on a - Axis Small or Missing .... 0.00000 Ang.
PLAT143_ALERT_4_C s.u. on c - Axis Small or Missing .... 0.00000 Ang.
PLAT151_ALERT_1_C No s.u. (esd) Given on Volume .... Please Do !
PLAT213_ALERT_2_C Atom C72 has ADP max/min Ratio ..... 3.2 prolat
PLAT213_ALERT_2_C Atom C93 has ADP max/min Ratio ..... 3.4 prolat
PLAT220_ALERT_2_C NonSolvent Resd 1 C Ueq(max)/Ueq(min) Range 4.9 Ratio
PLAT222_ALERT_3_C NonSolvent Resd 1 H Uiso(max)/Uiso(min) Range 4.6 Ratio
PLAT230_ALERT_2_C Hirshfeld Test Diff for N2 --C13 . 5.3 s.u.
PLAT230_ALERT_2_C Hirshfeld Test Diff for C66 --C67 . 6.0 s.u.
PLAT234_ALERT_4_C Large Hirshfeld Difference C4 --C5 . 0.18 Ang.
And 2 other PLAT234 Alerts
More ...

PLAT241_ALERT_2_C High 'MainMol' Ueq as Compared to Neighbors of C19 Check
And 6 other PLAT241 Alerts
More ...
PLAT242_ALERT_2_C Low 'MainMol' Ueq as Compared to Neighbors of C17 Check
And 3 other PLAT242 Alerts
More ...
PLAT243_ALERT_4_C High 'Solvent' Ueq as Compared to Neighbors of C98 Check
PLAT243_ALERT_4_C High 'Solvent' Ueq as Compared to Neighbors of C100 Check
PLAT244_ALERT_4_C Low 'Solvent' Ueq as Compared to Neighbors of C97 Check
And 2 other PLAT244 Alerts
More ...
PLAT250_ALERT_2_C Large U3/U1 Ratio for <U(i,j)> Tensor(Resd 2) 3.1 Note
PLAT260_ALERT_2_C Large Average Ueq of Residue Including C97 0.292 Check
PLAT334_ALERT_2_C Small <C-C> Benzene Dist. C59 -C64 . 1.37 Ang.
PLAT334_ALERT_2_C Small <C-C> Benzene Dist. C91 -C96 . 1.36 Ang.
PLAT342_ALERT_3_C Low Bond Precision on C-C Bonds .... 0.01382 Ang.
PLAT411_ALERT_2_C Short Inter H...H Contact H90 ..H98B . 2.07 Ang.
x,y,z = 1_555 Check
And 2 other PLAT411 Alerts
More ...

Alert level G

ABSMU01_ALERT_1_G Calculation of _exptl_absorpt_correction_mu
not performed for this radiation type.
PLAT002_ALERT_2_G Number of Distance or Angle Restraints on AtSite 6 Note
PLAT003_ALERT_2_G Number of Uiso or U(i,j) Restrained non-H-Atoms 12 Report
PLAT012_ALERT_1_G No _shelx_res_checksum Found in CIF ...... Please Check
PLAT072_ALERT_2_G SHELXL First Parameter in WGHT Unusually Large 0.16 Report
PLAT083_ALERT_2_G SHELXL Second Parameter in WGHT Unusually Large 76.84 Why ?
PLAT172_ALERT_4_G The CIF-Embedded .res File Contains DFIX Records 1 Report
PLAT173_ALERT_4_G The CIF-Embedded .res File Contains DANG Records 1 Report
PLAT176_ALERT_4_G The CIF-Embedded .res File Contains SADI Records 2 Report
PLAT177_ALERT_4_G The CIF-Embedded .res File Contains DELU Records 1 Report
PLAT178_ALERT_4_G The CIF-Embedded .res File Contains SIMU Records 2 Report
PLAT187_ALERT_4_G The CIF-Embedded .res File Contains RIGU Records 1 Report
PLAT188_ALERT_3_G A Non-default SIMU Restraint Value has been used 0.0100 Report
PLAT191_ALERT_3_G A Non-default SADI Restraint Value has been used 0.0400 Report
PLAT192_ALERT_3_G A Non-default DELU Restraint Value for SecondPar 0.0200 Report
PLAT199_ALERT_1_G Reported _cell_measurement_temperature ..... (K) 293 Check
PLAT200_ALERT_1_G Reported _diffrn_ambient_temperature ..... (K) 293 Check
PLAT606_ALERT_4_G Solvent Accessible VOID(S) in Structure .... ! Info
PLAT860_ALERT_3_G Number of Least-Squares Restraints .... 161 Note
PLAT868_ALERT_4_G ALERTS Due to the Use of _smtbx_masks Suppressed ! Info
PLAT933_ALERT_2_G Number of HKL-OMIT Records in Embedded .res File 37 Note
-7 8 0, -5 7 0, -4 3 1, -4 3 2, -4 5 1, -3 2 2,
-3 4 1, -3 5 2, -3 6 2, -2 2 1, -2 2 2, -2 2 4,
-2 3 1, -2 4 2, -2 4 3, -2 4 5, -2 6 0, -1 1 1,
-1 1 2, -1 2 1, -1 2 2, -1 2 3, -1 2 4, -1 3 0,
-1 4 0, -1 4 2, 0 0 2, 0 0 4, 0 2 0, 0 2 2,
0 3 0, 0 6 1, 1 1 2, 1 1 3, 1 2 2, 1 4 1,
2 2 2,
PLAT984_ALERT_1_G The I-f'= -0.4617 Deviates from the B&C-Value -0.4875 Check
PLAT985_ALERT_1_G The I-f"= 1.7363 Deviates from the B&C-Value 1.7244 Check

---

0 **ALERT level A** = Most likely a serious problem - resolve or explain
6 **ALERT level B** = A potentially serious problem, consider carefully
36 **ALERT level C** = Check. Ensure it is not caused by an omission or oversight
23 **ALERT level G** = General information/check it is not something unexpected

7 ALERT type 1 CIF construction/syntax error, inconsistent or missing data
33 ALERT type 2 Indicator that the structure model may be wrong or deficient
7 ALERT type 3 Indicator that the structure quality may be low
18 ALERT type 4 Improvement, methodology, query or suggestion
0 ALERT type 5 Informative message, check

It is advisable to attempt to resolve as many as possible of the alerts in all categories. Often the minor alerts point to easily fixed oversights, errors and omissions in your CIF or refinement strategy, so attention to these fine details can be worthwhile. In order to resolve some of the more serious problems it may be necessary to carry out additional measurements or structure refinements. However, the purpose of your study may justify the reported deviations and the more serious of these should normally be commented upon in the discussion or experimental section of a paper or in the "special_details" fields of the CIF. checkCIF was carefully designed to identify outliers and unusual parameters, but every test has its limitations and alerts that are not important in a particular case may appear. Conversely, the absence of alerts does not guarantee there are no

aspects of the results needing attention. It is up to the individual to critically assess their own results and, if necessary, seek expert advice.

Publication of your CIF in IUCr journals

A basic structural check has been run on your CIF. These basic checks will be run on all CIFs submitted for publication in IUCr journals (*Acta Crystallographica*, *Journal of Applied Crystallography*, *Journal of Synchrotron Radiation*); however, if you intend to submit to *Acta Crystallographica Section C* or *E* or *IUCrData*, you should make sure that full publication checks are run on the final version of your CIF prior to submission.

Publication of your CIF in other journals

Please refer to the *Notes for Authors* of the relevant journal for any special instructions relating to CIF submission.

PLATON version of 02/02/2025; check.def file version of 02/02/2025

Datablock wy222_0m - ellipsoid plot

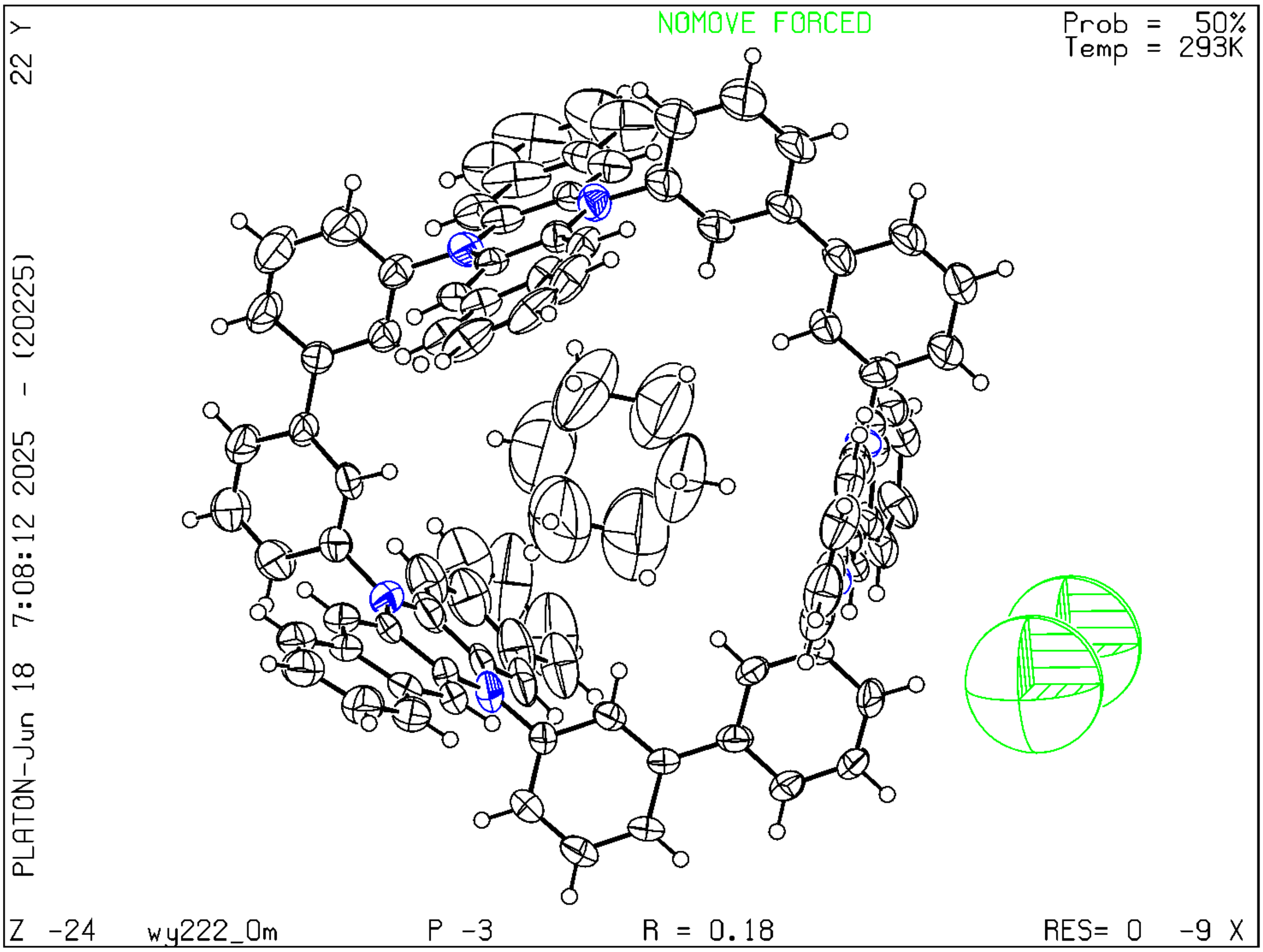

Download CIF editor (publCIF) from the IUCr
Download CIF editor (enCIFer) from the CCDC
Test a new CIF entry
