# Supplementary material for: Supramolecular Macrocyclic Iodine Adsorbents Enable Photothermally Stable Perovskite Solar Cells
Source: Adv Sci (Weinh). 2025 Oct 30;13(4):e16964. doi: 10.1002/advs.202516964 (PMC12822466; doi:10.1002/advs.202516964)

checkCIF/PLATON report

Structure factors have been supplied for datablock(s) exp_4348_auto

THIS REPORT IS FOR GUIDANCE ONLY. IF USED AS PART OF A REVIEW PROCEDURE FOR PUBLICATION, IT SHOULD NOT REPLACE THE EXPERTISE OF AN EXPERIENCED CRYSTALLOGRAPHIC REFEREE.

No syntax errors found.   CIF dictionary   Interpreting this report

Datablock: exp_4348_auto

Bond precision: C-C = 0.0136 A   Wavelength=1.54184

Cell:	a=19.3678(7)	b=27.5928(13)	c=27.6484(12)
	alpha=60.330(5)	beta=89.675(3)	gamma=89.110(3)
Temperature:	173 K		

	Calculated	Reported
Volume	12836.8(11)	12836.8(11)
Space group	P -1	P -1
Hall group	-P 1	-P 1
Moiety formula	C96 H60 N6 [+ solvent]	3(C96 H60 N6)
Sum formula	C96 H60 N6 [+ solvent]	C288 H180 N18
Mr	1297.50	3892.49
Dx,g cm-3	1.007	1.007
Z	6	2
Mu (mm-1)	0.454	0.454
F000	4068.0	4068.0
F000’	4078.88	
h,k,lmax	23,32,33	23,32,33
Nref	45823	45595
Tmin,Tmax	0.868,0.905	0.775,1.000
Tmin’	0.865	

Correction method= # Reported T Limits: Tmin=0.775 Tmax=1.000
AbsCorr = MULTI-SCAN

Data completeness= 0.995   Theta(max)= 67.080

R(reflections)= 0.1534(21164)   wR2(reflections)= 0.4027(45595)

S = 0.985   Npar= 2786

The following ALERTS were generated. Each ALERT has the format
test-name_ALERT_alert-type_alert-level.
Click on the hyperlinks for more details of the test.

Alert level B

```
PLAT084_ALERT_3_B High wR2 Value (i.e. > 0.25) ...................       0.40 Report
PLAT230_ALERT_2_B Hirshfeld Test Diff for    C26       --C27      .      11.5 s.u.
PLAT230_ALERT_2_B Hirshfeld Test Diff for    C31       --C36      .       9.8 s.u.
PLAT230_ALERT_2_B Hirshfeld Test Diff for    N12       --C113     .       7.8 s.u.
PLAT230_ALERT_2_B Hirshfeld Test Diff for    C121      --C126     .       7.6 s.u.
PLAT230_ALERT_2_B Hirshfeld Test Diff for    C123      --C124     .       7.8 s.u.
PLAT230_ALERT_2_B Hirshfeld Test Diff for    C141      --C146     .       8.0 s.u.
PLAT230_ALERT_2_B Hirshfeld Test Diff for    C151      --C152     .       9.1 s.u.
PLAT230_ALERT_2_B Hirshfeld Test Diff for    C201      --C206     .       8.3 s.u.
PLAT230_ALERT_2_B Hirshfeld Test Diff for    C203      --C204     .      16.1 s.u.
PLAT230_ALERT_2_B Hirshfeld Test Diff for    C230      --C231     .       7.9 s.u.
PLAT230_ALERT_2_B Hirshfeld Test Diff for    C246      --C247     .       8.5 s.u.
PLAT230_ALERT_2_B Hirshfeld Test Diff for    C251      --C256     .      11.6 s.u.
PLAT230_ALERT_2_B Hirshfeld Test Diff for    C253      --C254     .       9.4 s.u.
PLAT230_ALERT_2_B Hirshfeld Test Diff for    C269      --C270     .       7.1 s.u.
PLAT241_ALERT_2_B High   'MainMol' Ueq as Compared to Neighbors of       C204 Check
PLAT340_ALERT_3_B Low Bond Precision on  C-C Bonds ..............    0.01362 Ang.
```

Alert level C

```
DIFMX02_ALERT_1_C  The maximum difference density is > 0.1*ZMAX*0.75
            The relevant atom site should be identified.
RINTA01_ALERT_3_C  The value of Rint is greater than 0.12
            Rint given   0.144
PLAT020_ALERT_3_C The Value of Rint is Greater Than 0.12 .........      0.144 Report
PLAT026_ALERT_3_C Ratio Observed / Unique Reflections (too) Low ..        46% Check
PLAT082_ALERT_2_C High R1 Value ..................................       0.15 Report
PLAT213_ALERT_2_C Atom C204            has ADP max/min Ratio .....        3.4 prolat
PLAT220_ALERT_2_C NonSolvent   Resd 3  C   Ueq(max)/Ueq(min) Range        3.5 Ratio
PLAT230_ALERT_2_C Hirshfeld Test Diff for    C1        --C6       .       5.3 s.u.
PLAT230_ALERT_2_C Hirshfeld Test Diff for    C8        --C9       .       5.4 s.u.
PLAT230_ALERT_2_C Hirshfeld Test Diff for    C16       --C17      .       6.9 s.u.
PLAT230_ALERT_2_C Hirshfeld Test Diff for    C21       --C26      .       5.1 s.u.
PLAT230_ALERT_2_C Hirshfeld Test Diff for    C28       --C29      .       5.4 s.u.
PLAT230_ALERT_2_C Hirshfeld Test Diff for    C42       --C43      .       5.2 s.u.
PLAT230_ALERT_2_C Hirshfeld Test Diff for    C43       --C44      .       6.9 s.u.
PLAT230_ALERT_2_C Hirshfeld Test Diff for    C46       --C47      .       5.7 s.u.
PLAT230_ALERT_2_C Hirshfeld Test Diff for    C56       --C57      .       5.8 s.u.
PLAT230_ALERT_2_C Hirshfeld Test Diff for    C117      --C118     .       5.1 s.u.
PLAT230_ALERT_2_C Hirshfeld Test Diff for    C147      --C148     .       5.5 s.u.
PLAT230_ALERT_2_C Hirshfeld Test Diff for    C161      --C167     .       5.3 s.u.
PLAT230_ALERT_2_C Hirshfeld Test Diff for    C217      --C218     .       5.5 s.u.
PLAT230_ALERT_2_C Hirshfeld Test Diff for    C219      --C228     .       5.2 s.u.
PLAT230_ALERT_2_C Hirshfeld Test Diff for    C225      --C226     .       6.1 s.u.
PLAT230_ALERT_2_C Hirshfeld Test Diff for    C247      --C248     .       5.4 s.u.
PLAT230_ALERT_2_C Hirshfeld Test Diff for    C259      --C265     .       5.4 s.u.
PLAT230_ALERT_2_C Hirshfeld Test Diff for    C272      --C273     .       6.0 s.u.
PLAT230_ALERT_2_C Hirshfeld Test Diff for    C274      --C275     .       5.7 s.u.
PLAT234_ALERT_4_C Large Hirshfeld Difference N2        --C12      .      0.17 Ang.
PLAT234_ALERT_4_C Large Hirshfeld Difference N3        --C29      .      0.16 Ang.
```

```
PLAT234_ALERT_4_C Large Hirshfeld Difference N4       --C28       .       0.18 Ang.
PLAT234_ALERT_4_C Large Hirshfeld Difference C3       --C4        .       0.17 Ang.
PLAT234_ALERT_4_C Large Hirshfeld Difference C9       --C10       .       0.16 Ang.
PLAT234_ALERT_4_C Large Hirshfeld Difference C15      --C16       .       0.17 Ang.
PLAT234_ALERT_4_C Large Hirshfeld Difference C17      --C18       .       0.21 Ang.
PLAT234_ALERT_4_C Large Hirshfeld Difference C19      --C20       .       0.16 Ang.
PLAT234_ALERT_4_C Large Hirshfeld Difference C36      --C37       .       0.23 Ang.
PLAT234_ALERT_4_C Large Hirshfeld Difference C37      --C38       .       0.19 Ang.
PLAT234_ALERT_4_C Large Hirshfeld Difference C38      --C39       .       0.23 Ang.
PLAT234_ALERT_4_C Large Hirshfeld Difference C39      --C40       .       0.19 Ang.
PLAT234_ALERT_4_C Large Hirshfeld Difference C52      --C53       .       0.18 Ang.
PLAT234_ALERT_4_C Large Hirshfeld Difference C54      --C59       .       0.19 Ang.
PLAT234_ALERT_4_C Large Hirshfeld Difference C73      --C74       .       0.17 Ang.
PLAT234_ALERT_4_C Large Hirshfeld Difference C75      --C76       .       0.20 Ang.
PLAT234_ALERT_4_C Large Hirshfeld Difference C80      --C81       .       0.17 Ang.
PLAT234_ALERT_4_C Large Hirshfeld Difference C81      --C82       .       0.20 Ang.
PLAT234_ALERT_4_C Large Hirshfeld Difference C87      --C88       .       0.19 Ang.
PLAT234_ALERT_4_C Large Hirshfeld Difference C87      --C91       .       0.16 Ang.
PLAT234_ALERT_4_C Large Hirshfeld Difference C88      --C89       .       0.20 Ang.
PLAT234_ALERT_4_C Large Hirshfeld Difference C92      --C93       .       0.16 Ang.
PLAT234_ALERT_4_C Large Hirshfeld Difference C94      --C95       .       0.16 Ang.
PLAT234_ALERT_4_C Large Hirshfeld Difference N9       --C171      .       0.17 Ang.
PLAT234_ALERT_4_C Large Hirshfeld Difference C104     --C105      .       0.16 Ang.
PLAT234_ALERT_4_C Large Hirshfeld Difference C107     --C108      .       0.16 Ang.
PLAT234_ALERT_4_C Large Hirshfeld Difference C111     --C120      .       0.17 Ang.
PLAT234_ALERT_4_C Large Hirshfeld Difference C115     --C120      .       0.16 Ang.
PLAT234_ALERT_4_C Large Hirshfeld Difference C118     --C119      .       0.18 Ang.
PLAT234_ALERT_4_C Large Hirshfeld Difference C121     --C122      .       0.22 Ang.
PLAT234_ALERT_4_C Large Hirshfeld Difference C124     --C125      .       0.21 Ang.
PLAT234_ALERT_4_C Large Hirshfeld Difference C125     --C126      .       0.17 Ang.
PLAT234_ALERT_4_C Large Hirshfeld Difference C131     --C140      .       0.18 Ang.
PLAT234_ALERT_4_C Large Hirshfeld Difference C133     --C134      .       0.16 Ang.
PLAT234_ALERT_4_C Large Hirshfeld Difference C134     --C135      .       0.20 Ang.
PLAT234_ALERT_4_C Large Hirshfeld Difference C173     --C174      .       0.16 Ang.
PLAT234_ALERT_4_C Large Hirshfeld Difference C175     --C176      .       0.20 Ang.
PLAT234_ALERT_4_C Large Hirshfeld Difference C176     --C177      .       0.18 Ang.
PLAT234_ALERT_4_C Large Hirshfeld Difference C177     --C178      .       0.20 Ang.
PLAT234_ALERT_4_C Large Hirshfeld Difference C180     --C181      .       0.16 Ang.
PLAT234_ALERT_4_C Large Hirshfeld Difference C193     --C194      .       0.18 Ang.
PLAT234_ALERT_4_C Large Hirshfeld Difference N18      --C227      .       0.16 Ang.
PLAT234_ALERT_4_C Large Hirshfeld Difference C201     --C202      .       0.18 Ang.
PLAT234_ALERT_4_C Large Hirshfeld Difference C202     --C203      .       0.18 Ang.
PLAT234_ALERT_4_C Large Hirshfeld Difference C204     --C205      .       0.24 Ang.
PLAT234_ALERT_4_C Large Hirshfeld Difference C209     --C283      .       0.16 Ang.
PLAT234_ALERT_4_C Large Hirshfeld Difference C218     --C296      .       0.18 Ang.
PLAT234_ALERT_4_C Large Hirshfeld Difference C219     --C220      .       0.18 Ang.
PLAT234_ALERT_4_C Large Hirshfeld Difference C223     --C224      .       0.19 Ang.
PLAT234_ALERT_4_C Large Hirshfeld Difference C229     --C230      .       0.16 Ang.
PLAT234_ALERT_4_C Large Hirshfeld Difference C231     --C232      .       0.19 Ang.
PLAT234_ALERT_4_C Large Hirshfeld Difference C232     --C233      .       0.17 Ang.
PLAT234_ALERT_4_C Large Hirshfeld Difference C233     --C234      .       0.16 Ang.
PLAT234_ALERT_4_C Large Hirshfeld Difference C234     --C235      .       0.19 Ang.
PLAT234_ALERT_4_C Large Hirshfeld Difference C239     --C248      .       0.17 Ang.
PLAT234_ALERT_4_C Large Hirshfeld Difference C241     --C242      .       0.23 Ang.
PLAT234_ALERT_4_C Large Hirshfeld Difference C250     --C251      .       0.18 Ang.
PLAT234_ALERT_4_C Large Hirshfeld Difference C252     --C253      .       0.19 Ang.
PLAT234_ALERT_4_C Large Hirshfeld Difference C254     --C255      .       0.21 Ang.
```

```
PLAT234_ALERT_4_C Large Hirshfeld Difference C259     --C260     .        0.17 Ang.
PLAT234_ALERT_4_C Large Hirshfeld Difference C259     --C264     .        0.17 Ang.
PLAT234_ALERT_4_C Large Hirshfeld Difference C260     --C261     .        0.21 Ang.
PLAT234_ALERT_4_C Large Hirshfeld Difference C262     --C263     .        0.20 Ang.
PLAT234_ALERT_4_C Large Hirshfeld Difference C263     --C264     .        0.16 Ang.
PLAT234_ALERT_4_C Large Hirshfeld Difference C265     --C270     .        0.16 Ang.
PLAT234_ALERT_4_C Large Hirshfeld Difference C279     --C280     .        0.18 Ang.
PLAT234_ALERT_4_C Large Hirshfeld Difference C284     --C285     .        0.18 Ang.
PLAT234_ALERT_4_C Large Hirshfeld Difference C292     --C293     .        0.16 Ang.
PLAT241_ALERT_2_C High   'MainMol' Ueq as Compared to Neighbors of          C3 Check
PLAT241_ALERT_2_C High   'MainMol' Ueq as Compared to Neighbors of         C17 Check
PLAT241_ALERT_2_C High   'MainMol' Ueq as Compared to Neighbors of         C27 Check
PLAT241_ALERT_2_C High   'MainMol' Ueq as Compared to Neighbors of         C37 Check
PLAT241_ALERT_2_C High   'MainMol' Ueq as Compared to Neighbors of         C43 Check
PLAT241_ALERT_2_C High   'MainMol' Ueq as Compared to Neighbors of         C56 Check
PLAT241_ALERT_2_C High   'MainMol' Ueq as Compared to Neighbors of         C69 Check
PLAT241_ALERT_2_C High   'MainMol' Ueq as Compared to Neighbors of         C70 Check
PLAT241_ALERT_2_C High   'MainMol' Ueq as Compared to Neighbors of         C82 Check
PLAT241_ALERT_2_C High   'MainMol' Ueq as Compared to Neighbors of        C118 Check
PLAT241_ALERT_2_C High   'MainMol' Ueq as Compared to Neighbors of        C124 Check
PLAT241_ALERT_2_C High   'MainMol' Ueq as Compared to Neighbors of        C143 Check
PLAT241_ALERT_2_C High   'MainMol' Ueq as Compared to Neighbors of        C157 Check
PLAT241_ALERT_2_C High   'MainMol' Ueq as Compared to Neighbors of        C159 Check
PLAT241_ALERT_2_C High   'MainMol' Ueq as Compared to Neighbors of        C165 Check
PLAT241_ALERT_2_C High   'MainMol' Ueq as Compared to Neighbors of        C170 Check
PLAT241_ALERT_2_C High   'MainMol' Ueq as Compared to Neighbors of        C176 Check
PLAT241_ALERT_2_C High   'MainMol' Ueq as Compared to Neighbors of        C188 Check
PLAT241_ALERT_2_C High   'MainMol' Ueq as Compared to Neighbors of        C190 Check
PLAT241_ALERT_2_C High   'MainMol' Ueq as Compared to Neighbors of        C192 Check
PLAT241_ALERT_2_C High   'MainMol' Ueq as Compared to Neighbors of        C194 Check
PLAT241_ALERT_2_C High   'MainMol' Ueq as Compared to Neighbors of        C218 Check
PLAT241_ALERT_2_C High   'MainMol' Ueq as Compared to Neighbors of        C241 Check
PLAT241_ALERT_2_C High   'MainMol' Ueq as Compared to Neighbors of        C255 Check
PLAT241_ALERT_2_C High   'MainMol' Ueq as Compared to Neighbors of        C261 Check
PLAT241_ALERT_2_C High   'MainMol' Ueq as Compared to Neighbors of        C269 Check
PLAT241_ALERT_2_C High   'MainMol' Ueq as Compared to Neighbors of        C280 Check
PLAT241_ALERT_2_C High   'MainMol' Ueq as Compared to Neighbors of        C281 Check
PLAT241_ALERT_2_C High   'MainMol' Ueq as Compared to Neighbors of        C295 Check
PLAT242_ALERT_2_C Low    'MainMol' Ueq as Compared to Neighbors of         C26 Check
PLAT242_ALERT_2_C Low    'MainMol' Ueq as Compared to Neighbors of         C42 Check
PLAT242_ALERT_2_C Low    'MainMol' Ueq as Compared to Neighbors of         C84 Check
PLAT242_ALERT_2_C Low    'MainMol' Ueq as Compared to Neighbors of        C160 Check
PLAT242_ALERT_2_C Low    'MainMol' Ueq as Compared to Neighbors of        C181 Check
PLAT242_ALERT_2_C Low    'MainMol' Ueq as Compared to Neighbors of        C195 Check
PLAT242_ALERT_2_C Low    'MainMol' Ueq as Compared to Neighbors of        C203 Check
PLAT242_ALERT_2_C Low    'MainMol' Ueq as Compared to Neighbors of        C205 Check
PLAT242_ALERT_2_C Low    'MainMol' Ueq as Compared to Neighbors of        C224 Check
PLAT242_ALERT_2_C Low    'MainMol' Ueq as Compared to Neighbors of        C236 Check
PLAT242_ALERT_2_C Low    'MainMol' Ueq as Compared to Neighbors of        C240 Check
PLAT242_ALERT_2_C Low    'MainMol' Ueq as Compared to Neighbors of        C254 Check
PLAT242_ALERT_2_C Low    'MainMol' Ueq as Compared to Neighbors of        C264 Check
PLAT242_ALERT_2_C Low    'MainMol' Ueq as Compared to Neighbors of        C277 Check
PLAT242_ALERT_2_C Low    'MainMol' Ueq as Compared to Neighbors of        C279 Check
PLAT242_ALERT_2_C Low    'MainMol' Ueq as Compared to Neighbors of        C290 Check
PLAT334_ALERT_2_C Small <C-C> Benzene Dist.   C67      -C72      .        1.37 Ang.
PLAT334_ALERT_2_C Small <C-C> Benzene Dist.   C161     -C166     .        1.36 Ang.
PLAT334_ALERT_2_C Small <C-C> Benzene Dist.   C167     -C172     .        1.37 Ang.
```

```
PLAT334_ALERT_2_C Small <C-C> Benzene Dist.   C259     -C264     .       1.37 Ang.
PLAT334_ALERT_2_C Small <C-C> Benzene Dist.   C277     -C282     .       1.35 Ang.
PLAT369_ALERT_2_C Long   C(sp2)-C(sp2) Bond  C217     - C218     .       1.53 Ang.
PLAT369_ALERT_2_C Long   C(sp2)-C(sp2) Bond  C259     - C265     .       1.53 Ang.
PLAT790_ALERT_4_C Centre of Gravity not Within Unit Cell: Resd.  #          1 Note
             C96 H60 N6
PLAT905_ALERT_3_C Negative K value in the Analysis of Variance ...     -1.384 Report
PLAT906_ALERT_3_C Large K Value in the Analysis of Variance ......      2.522 Check
PLAT906_ALERT_3_C Large K Value in the Analysis of Variance ......      2.184 Check
PLAT911_ALERT_3_C Missing FCF Refl Between Thmin & STh/L=    0.597        221 Report
                2  0  0,   4  0  0,  -2  1  0,   0  2  0,   0  3  0,  22  8  0,
               22  9  0, -22 -8  1,  -4 -4  1,  -1 -2  1,   0 -2  1,  -3 -1  1,
               -1 -1  1,   0 -1  1,   2 -1  1,   4 -1  1,  -1  0  1,  -2  1  1,
               -1  1  1,   1  2  1,  -1  3  1,   2  3  1,  22  8  1,  22  9  1,
                6 -5  2,   0 -2  2,   0 -1  2,   1 -1  2,   1  0  2,  -1  1  2,
                0  1  2,   1  1  2,  -1  2  2,   0  2  2,   1  2  2,  -1  3  2,
                1  3  2,  23  4  2,   4  6  2,  22  7  2,  22  8  2,  22  9  2,
                2 10  2,  22 10  2, -16 21  2,   0 -3  3,   0  0  3,  -1  1  3,
                1  1  3,   2  2  3,   0  3  3,  23  3  3,   1  5  3,  22  6  3,
               22  7  3,  22  8  3,  21  9  3,  22  9  3,  21 10  3,  22 10  3,
                1 11  3,  21 11  3,   2 -1  4,  -1  1  4,   2  3  4,  22  5  4,
                1  6  4,  22  6  4,  21  7  4,  22  7  4,  21  8  4,  22  8  4,
               21  9  4,  22  9  4,  21 10  4,  22 10  4,  21 11  4,  21 12  4,
               21 13  4, -15 23  4,  22  5  5,  22  6  5,  21  7  5,  22  7  5,
               20  8  5,  21  8  5,  22  8  5,  20  9  5,  21  9  5,  22  9  5,
               20 10  5,  21 10  5,  22 10  5,  21 11  5,  21 12  5,  21 13  5,
```

Alert level G

```
PLAT002_ALERT_2_G Number of Distance or Angle Restraints on AtSite         14 Note
PLAT003_ALERT_2_G Number of Uiso or U(i,j) Restrained non-H Atoms          12 Report
PLAT042_ALERT_1_G Calc. and Reported MoietyFormula Strings  Differ     Please Check
             Calc: C96 H60 N6
             Rep.: 3(C96 H60 N6)
PLAT045_ALERT_1_G Calculated and Reported Z Differ by a Factor ...          3 Check
PLAT083_ALERT_2_G SHELXL Second Parameter in WGHT  Unusually Large      45.78 Why ?
PLAT176_ALERT_4_G The CIF-Embedded .res File Contains SADI Records         18 Report
PLAT177_ALERT_4_G The CIF-Embedded .res File Contains DELU Records          1 Report
PLAT178_ALERT_4_G The CIF-Embedded .res File Contains SIMU Records          1 Report
PLAT188_ALERT_3_G A Non-default SIMU Restraint Value has been used     0.0100 Report
PLAT191_ALERT_3_G A Non-default SADI Restraint Value has been used     0.0400 Report
PLAT191_ALERT_3_G A Non-default SADI Restraint Value has been used     0.0400 Report
PLAT191_ALERT_3_G A Non-default SADI Restraint Value has been used     0.0400 Report
PLAT191_ALERT_3_G A Non-default SADI Restraint Value has been used     0.0400 Report
PLAT191_ALERT_3_G A Non-default SADI Restraint Value has been used     0.0400 Report
PLAT191_ALERT_3_G A Non-default SADI Restraint Value has been used     0.0400 Report
PLAT191_ALERT_3_G A Non-default SADI Restraint Value has been used     0.0400 Report
PLAT191_ALERT_3_G A Non-default SADI Restraint Value has been used     0.0400 Report
PLAT191_ALERT_3_G A Non-default SADI Restraint Value has been used     0.0400 Report
PLAT191_ALERT_3_G A Non-default SADI Restraint Value has been used     0.0400 Report
PLAT192_ALERT_3_G A Non-default DELU Restraint Value for SecondPar     0.0200 Report
PLAT301_ALERT_3_G Main Residue  Disorder .............(Resd    1)          6% Note
PLAT335_ALERT_2_G Check Large C6 Ring C-C Range C1       -C6             0.15 Ang.
PLAT335_ALERT_2_G Check Large C6 Ring C-C Range C1       -C10            0.18 Ang.
PLAT335_ALERT_2_G Check Large C6 Ring C-C Range C11      -C20            0.17 Ang.
PLAT335_ALERT_2_G Check Large C6 Ring C-C Range C14      -C19            0.19 Ang.
PLAT335_ALERT_2_G Check Large C6 Ring C-C Range C21      -C30            0.21 Ang.
```

```
PLAT335_ALERT_2_G Check Large C6 Ring C-C Range C31      -C40           0.18 Ang.
PLAT335_ALERT_2_G Check Large C6 Ring C-C Range C41      -C50           0.15 Ang.
PLAT335_ALERT_2_G Check Large C6 Ring C-C Range C54      -C59           0.19 Ang.
PLAT335_ALERT_2_G Check Large C6 Ring C-C Range C101     -C106          0.19 Ang.
PLAT335_ALERT_2_G Check Large C6 Ring C-C Range C111     -C120          0.22 Ang.
PLAT335_ALERT_2_G Check Large C6 Ring C-C Range C141     -C150          0.18 Ang.
PLAT335_ALERT_2_G Check Large C6 Ring C-C Range C155     -C160          0.16 Ang.
PLAT335_ALERT_2_G Check Large C6 Ring C-C Range C201     -C206          0.22 Ang.
PLAT335_ALERT_2_G Check Large C6 Ring C-C Range C201     -C283          0.21 Ang.
PLAT335_ALERT_2_G Check Large C6 Ring C-C Range C210     -C296          0.19 Ang.
PLAT335_ALERT_2_G Check Large C6 Ring C-C Range C239     -C244          0.18 Ang.
PLAT335_ALERT_2_G Check Large C6 Ring C-C Range C239     -C248          0.15 Ang.
PLAT335_ALERT_2_G Check Large C6 Ring C-C Range C249     -C258          0.15 Ang.
PLAT335_ALERT_2_G Check Large C6 Ring C-C Range C277     -C282          0.21 Ang.
PLAT410_ALERT_2_G Short Intra H...H Contact  H62      ..H72      .       2.05 Ang.
                                                    x,y,z  =     1_555 Check
PLAT410_ALERT_2_G Short Intra H...H Contact  H66      ..H68      .       1.94 Ang.
                                                    x,y,z  =     1_555 Check
PLAT410_ALERT_2_G Short Intra H...H Contact  H68      ..H66A     .       2.12 Ang.
                                                    x,y,z  =     1_555 Check
PLAT410_ALERT_2_G Short Intra H...H Contact  H72      ..H62A     .       2.06 Ang.
                                                    x,y,z  =     1_555 Check
PLAT411_ALERT_2_G Short Inter H...H Contact  H280     ..H66A     .       1.83 Ang.
                                               1+x,y,-1+z  =     1_654 Check
PLAT432_ALERT_2_G Short Inter X...Y Contact  C280     ..C66A     .       3.11 Ang.
                                               1+x,y,-1+z  =     1_654 Check
PLAT606_ALERT_4_G Solvent Accessible VOID(S) in Structure ........          ! Info
PLAT790_ALERT_4_G Centre of Gravity not Within Unit Cell: Resd.  #          3 Note
              C96 H60 N6
PLAT860_ALERT_3_G Number of Least-Squares Restraints ............         222 Note
PLAT868_ALERT_4_G ALERTS Due to the Use of _smtbx_masks Suppressed          ! Info
PLAT910_ALERT_3_G Missing # of FCF Reflection(s) Below Theta(Min).          4 Note
                1  0  0,   0  1  0,   0  0  1,   0  1  1,
PLAT913_ALERT_3_G Missing # of Very Strong Reflections in FCF ....          3 Note
                0  3  0,   0  0  3,   0  3  3,
PLAT931_ALERT_5_G CIFcalcFCF  Twin Law ( 0 0 1)         Est.d BASF       0.08 Check
PLAT933_ALERT_2_G Number of HKL-OMIT Records in Embedded .res File         39 Note
               -5  4  6,  -5  4  9,  -4 -4  1,  -3 -1  1,  -2  1  0,  -2  1  1,
               -2 11 11,  -1 -2  1,  -1 -1  1,  -1  1  2,  -1  1  3,  -1  1  4,
               -1  2  2,  -1  3  1,  -1  3  2,   0 -3  3,   0 -2  1,   0 -2  2,
                0 -1  1,   0 -1  2,   0  0  3,   0  1  2,   0  2  2,   0  3  0,
                0  3  3,   1 -1  2,   1  0  2,   1  1  2,   1  1  3,   1  2  2,
                2 -1  1,   2 -1  4,   2  0  0,   2  2  3,   2  3  1,   2  3  4,
                4 -1  1,   4  0  0,   5 -2  6,
PLAT941_ALERT_3_G Average HKL Measurement Multiplicity ..........         3.5 Low
PLAT969_ALERT_5_G The 'Henn et al.' R-Factor-gap value ..........       2.121 Note
              Predicted wR2: Based on SigI**2 18.99 or SHELX Weight 40.88
PLAT978_ALERT_2_G Number C-C Bonds with Positive Residual Density.          0 Info
```

```
   0 ALERT level A = Most likely a serious problem - resolve or explain
  17 ALERT level B = A potentially serious problem, consider carefully
 151 ALERT level C = Check. Ensure it is not caused by an omission or oversight
  57 ALERT level G = General information/check it is not something unexpected

   3 ALERT type 1 CIF construction/syntax error, inconsistent or missing data
```

```
119 ALERT type 2 Indicator that the structure model may be wrong or deficient
 26 ALERT type 3 Indicator that the structure quality may be low
 75 ALERT type 4 Improvement, methodology, query or suggestion
  2 ALERT type 5 Informative message, check
```

It is advisable to attempt to resolve as many as possible of the alerts in all categories. Often the minor alerts point to easily fixed oversights, errors and omissions in your CIF or refinement strategy, so attention to these fine details can be worthwhile. In order to resolve some of the more serious problems it may be necessary to carry out additional measurements or structure refinements. However, the purpose of your study may justify the reported deviations and the more serious of these should normally be commented upon in the discussion or experimental section of a paper or in the "special_details" fields of the CIF. checkCIF was carefully designed to identify outliers and unusual parameters, but every test has its limitations and alerts that are not important in a particular case may appear. Conversely, the absence of alerts does not guarantee there are no aspects of the results needing attention. It is up to the individual to critically assess their own results and, if necessary, seek expert advice.

Publication of your CIF in IUCr journals

A basic structural check has been run on your CIF. These basic checks will be run on all CIFs submitted for publication in IUCr journals (*Acta Crystallographica*, *Journal of Applied Crystallography*, *Journal of Synchrotron Radiation*); however, if you intend to submit to *Acta Crystallographica Section C* or *E* or *IUCrData*, you should make sure that full publication checks are run on the final version of your CIF prior to submission.

Publication of your CIF in other journals

Please refer to the *Notes for Authors* of the relevant journal for any special instructions relating to CIF submission.

PLATON version of 13/05/2024; check.def file version of 04/05/2024

Datablock exp_4348_auto - ellipsoid plot

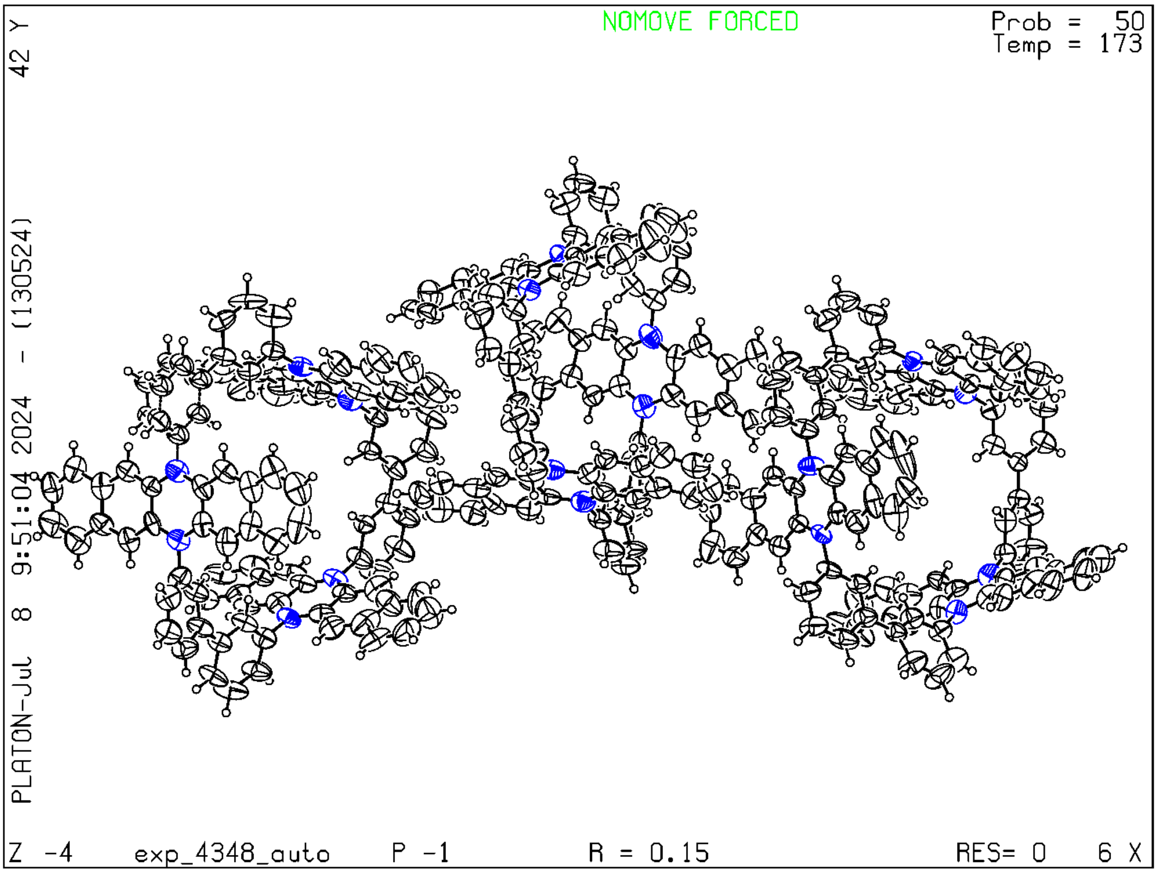

Supplement: Supplementary file 2 — Supplemental DataFile [file ADVS-13-e16964-s001.zip › M3_cifreport.pdf]
