# Supplementary material for: Supramolecular Macrocyclic Iodine Adsorbents Enable Photothermally Stable Perovskite Solar Cells
Source: Adv Sci (Weinh). 2025 Oct 30;13(4):e16964. doi: 10.1002/advs.202516964 (PMC12822466; doi:10.1002/advs.202516964)

checkCIF/PLATON report

Structure factors have been supplied for datablock(s) exp_4305_auto

THIS REPORT IS FOR GUIDANCE ONLY. IF USED AS PART OF A REVIEW PROCEDURE FOR PUBLICATION, IT SHOULD NOT REPLACE THE EXPERTISE OF AN EXPERIENCED CRYSTALLOGRAPHIC REFEREE.

No syntax errors found. CIF dictionary Interpreting this report

Datablock: exp_4305_auto

Bond precision:	C-C = 0.0088 A	Wavelength=1.54184	
Cell:	a=22.3314(3)	b=22.3314(3)	c=51.7257(12)
	alpha=90	beta=90	gamma=90
Temperature:	173 K		

	Calculated	Reported
Volume	25795.2(9)	25795.2(9)
Space group	I 41/a c d	I 41/a c d
Hall group	-I 4bd 2c	-I 4bd 2c
Moiety formula	C128 H80 N8, 4(C4 H8 O2), 2(C H Cl3) [+ solvent]	0.5(C H Cl3), C4 H8 O2, C32 H20 N2
Sum formula	C146 H114 Cl6 N8 O8 [+ solvent]	C36.50 H28.50 Cl1.50 N2 O2
Mr	2321.15	580.29
Dx,g cm-3	1.195	1.195
Z	8	32
Mu (mm-1)	1.689	1.689
F000	9696.0	9696.0
F000'	9738.53	
h,k,lmax	26,26,61	26,26,61
Nref	5773	5754
Tmin,Tmax	0.714,0.817	0.533,1.000
Tmin'	0.635	

Correction method= # Reported T Limits: Tmin=0.533 Tmax=1.000
AbsCorr = MULTI-SCAN

Data completeness= 0.997 Theta(max)= 67.072

R(reflections)= 0.1423(4346)   wR2(reflections)= 0.3114(5754)

S = 1.222   Npar= 452

The following ALERTS were generated. Each ALERT has the format
test-name_ALERT_alert-type_alert-level.
Click on the hyperlinks for more details of the test.

Alert level C

```
PLAT082_ALERT_2_C High R1 Value .................................       0.14 Report
PLAT084_ALERT_3_C High wR2 Value (i.e. > 0.25) ..................       0.31 Report
PLAT241_ALERT_2_C High   'MainMol' Ueq as Compared to Neighbors of       C25 Check
PLAT260_ALERT_2_C Large Average Ueq of Residue Including        O1      0.154 Check
PLAT260_ALERT_2_C Large Average Ueq of Residue Including       O1A      0.154 Check
PLAT260_ALERT_2_C Large Average Ueq of Residue Including       Cl1      0.219 Check
PLAT336_ALERT_2_C Long Bond Distance for ..... C37      -Cl2            1.910 Ang.
PLAT340_ALERT_3_C Low Bond Precision on  C-C Bonds ..............     0.00881 Ang.
PLAT906_ALERT_3_C Large K Value in the Analysis of Variance ......     29.199 Check
PLAT906_ALERT_3_C Large K Value in the Analysis of Variance ......      5.976 Check
PLAT906_ALERT_3_C Large K Value in the Analysis of Variance ......      2.888 Check
PLAT911_ALERT_3_C Missing FCF Refl Between Thmin & STh/L=    0.597        19 Report
                0  2  0,   2  4  0,   1  2  1,   2  3  1,   1  4  1,   0  2  2,
                1  3  2,   3  3  2,   0  4  2,   1  2  3,   0  0  4,   0  2  4,
                1  3  4,   1  4  5,   1  1  6,   0  2  6,   3  3  6,   0  4  6,
                0  0 12,
PLAT918_ALERT_3_C Reflection(s) with I(obs) much Smaller I(calc) .         2 Check
PLAT934_ALERT_3_C Number of (Iobs-Icalc)/Sigma(W) > 10 Outliers ..         1 Check
                2  3  5,
```

Alert level G

```
PLAT002_ALERT_2_G Number of Distance or Angle Restraints on AtSite        12 Note
PLAT003_ALERT_2_G Number of Uiso or U(i,j) Restrained non-H Atoms         12 Report
PLAT042_ALERT_1_G Calc. and Reported MoietyFormula Strings  Differ     Please Check
              Calc: C128 H80 N8, 4(C4 H8 O2), 2(C H Cl3)
              Rep.: 0.5(C H Cl3), C4 H8 O2, C32 H20 N2
PLAT045_ALERT_1_G Calculated and Reported Z Differ by a Factor ...      0.250 Check
PLAT083_ALERT_2_G SHELXL Second Parameter in WGHT  Unusually Large     105.00 Why ?
PLAT172_ALERT_4_G The CIF-Embedded .res File Contains DFIX Records         2 Report
PLAT173_ALERT_4_G The CIF-Embedded .res File Contains DANG Records         2 Report
PLAT176_ALERT_4_G The CIF-Embedded .res File Contains SADI Records        16 Report
PLAT177_ALERT_4_G The CIF-Embedded .res File Contains DELU Records         1 Report
PLAT178_ALERT_4_G The CIF-Embedded .res File Contains SIMU Records         2 Report
PLAT187_ALERT_4_G The CIF-Embedded .res File Contains RIGU Records         1 Report
PLAT188_ALERT_3_G A Non-default SIMU Restraint Value has been used     0.0100 Report
PLAT191_ALERT_3_G A Non-default SADI Restraint Value has been used     0.0400 Report
PLAT191_ALERT_3_G A Non-default SADI Restraint Value has been used     0.0400 Report
PLAT191_ALERT_3_G A Non-default SADI Restraint Value has been used     0.0400 Report
PLAT191_ALERT_3_G A Non-default SADI Restraint Value has been used     0.0400 Report
PLAT191_ALERT_3_G A Non-default SADI Restraint Value has been used     0.0400 Report
PLAT191_ALERT_3_G A Non-default SADI Restraint Value has been used     0.0400 Report
PLAT191_ALERT_3_G A Non-default SADI Restraint Value has been used     0.0400 Report
PLAT191_ALERT_3_G A Non-default SADI Restraint Value has been used     0.0400 Report
PLAT192_ALERT_3_G A Non-default DELU Restraint Value for SecondPar     0.0200 Report
```

```
PLAT300_ALERT_4_G Atom Site Occupancy of Cl1       Constrained at        0.5 Check
PLAT300_ALERT_4_G Atom Site Occupancy of Cl2       Constrained at        0.5 Check
PLAT300_ALERT_4_G Atom Site Occupancy of Cl3       Constrained at        0.5 Check
PLAT300_ALERT_4_G Atom Site Occupancy of C37       Constrained at        0.5 Check
PLAT300_ALERT_4_G Atom Site Occupancy of H37       Constrained at        0.5 Check
PLAT302_ALERT_4_G Anion/Solvent/Minor-Residue Disorder (Resd    2)       100% Note
PLAT302_ALERT_4_G Anion/Solvent/Minor-Residue Disorder (Resd    3)       100% Note
PLAT302_ALERT_4_G Anion/Solvent/Minor-Residue Disorder (Resd    4)       100% Note
PLAT304_ALERT_4_G Non-Integer Number of Atoms in ..... (Resd    2)       9.62 Check
PLAT304_ALERT_4_G Non-Integer Number of Atoms in ..... (Resd    3)       4.38 Check
PLAT304_ALERT_4_G Non-Integer Number of Atoms in ..... (Resd    4)       2.50 Check
PLAT411_ALERT_2_G Short Inter H...H Contact  H5       ..H33D     .       2.05 Ang.
                                          5/4-y,5/4-x,5/4-z  =      8_666 Check
PLAT411_ALERT_2_G Short Inter H...H Contact  H24      ..H36D     .       1.56 Ang.
                                                x,1/2+y,1-z  =     26_556 Check
PLAT605_ALERT_4_G Largest Solvent Accessible VOID in the Structure        18 A**3
PLAT860_ALERT_3_G Number of Least-Squares Restraints ............        315 Note
PLAT868_ALERT_4_G ALERTS Due to the Use of _smtbx_masks Suppressed          ! Info
PLAT909_ALERT_3_G Percentage of I>2sig(I) Data at Theta(Max) Still        53% Note
PLAT913_ALERT_3_G Missing # of Very Strong Reflections in FCF ....          2 Note
                0  4  6,   0  0 12,
PLAT933_ALERT_2_G Number of HKL-OMIT Records in Embedded .res File         18 Note
                0  0 12,   0  2  0,   0  2  2,   0  2  4,   0  2  6,   0  4  2,
                0  4  6,   1  1  6,   1  2  1,   1  2  3,   1  3  2,   1  3  4,
                1  4  1,   1  4  5,   2  3  1,   2  4  0,   3  3  2,   3  3  6,
PLAT969_ALERT_5_G The 'Henn et al.' R-Factor-gap value ..........      6.997 Note
              Predicted wR2: Based on SigI**2  4.45 or SHELX Weight 25.47
PLAT978_ALERT_2_G Number C-C Bonds with Positive Residual Density.          0 Info
```

---

 0 **ALERT level A** = Most likely a serious problem - resolve or explain
 0 **ALERT level B** = A potentially serious problem, consider carefully
14 **ALERT level C** = Check. Ensure it is not caused by an omission or oversight
42 **ALERT level G** = General information/check it is not something unexpected

```
 2 ALERT type 1 CIF construction/syntax error, inconsistent or missing data
13 ALERT type 2 Indicator that the structure model may be wrong or deficient
21 ALERT type 3 Indicator that the structure quality may be low
19 ALERT type 4 Improvement, methodology, query or suggestion
 1 ALERT type 5 Informative message, check
```

It is advisable to attempt to resolve as many as possible of the alerts in all categories. Often the minor alerts point to easily fixed oversights, errors and omissions in your CIF or refinement strategy, so attention to these fine details can be worthwhile. In order to resolve some of the more serious problems it may be necessary to carry out additional measurements or structure refinements. However, the purpose of your study may justify the reported deviations and the more serious of these should normally be commented upon in the discussion or experimental section of a paper or in the "special_details" fields of the CIF. checkCIF was carefully designed to identify outliers and unusual parameters, but every test has its limitations and alerts that are not important in a particular case may appear. Conversely, the absence of alerts does not guarantee there are no aspects of the results needing attention. It is up to the individual to critically assess their own results and, if necessary, seek expert advice.

Publication of your CIF in IUCr journals

A basic structural check has been run on your CIF. These basic checks will be run on all CIFs submitted for publication in IUCr journals (*Acta Crystallographica*, *Journal of Applied Crystallography*, *Journal of Synchrotron Radiation*); however, if you intend to submit to *Acta Crystallographica Section C* or *E* or *IUCrData*, you should make sure that full publication checks are run on the final version of your CIF prior to submission.

Publication of your CIF in other journals

Please refer to the *Notes for Authors* of the relevant journal for any special instructions relating to CIF submission.

PLATON version of 13/05/2024; check.def file version of 04/05/2024

Datablock exp_4305_auto - ellipsoid plot

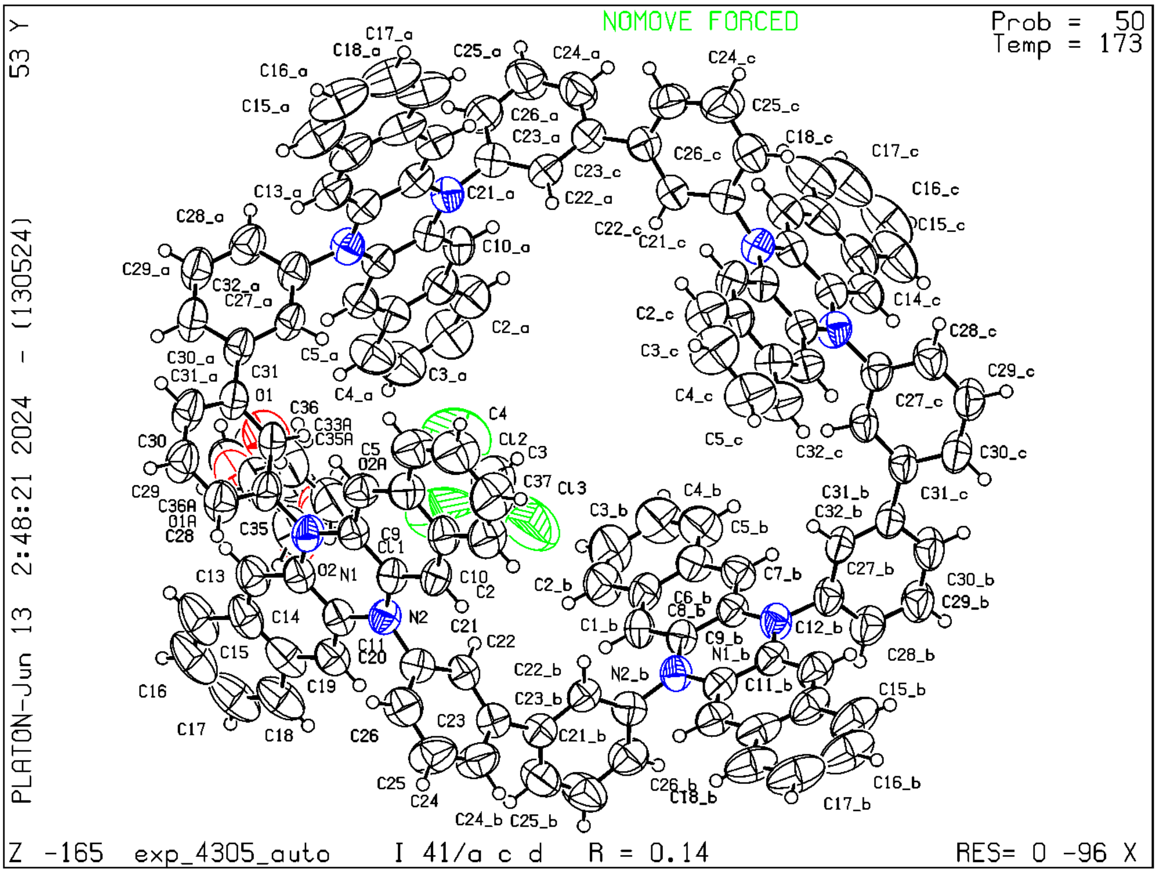

Supplement: Supplementary file 2 — Supplemental DataFile [file ADVS-13-e16964-s001.zip › M4_cifreport.pdf]
